# Facile Functionalization of Ambipolar, Nitrogen-Doped PAHs toward Highly Efficient TADF OLED Emitters

**DOI:** 10.1021/acsami.3c07552

**Published:** 2023-07-28

**Authors:** Jakub Wagner, Dharmendra Kumar, Michał Andrzej Kochman, Tomasz Gryber, Magdalena Grzelak, Adam Kubas, Przemysław Data, Marcin Lindner

**Affiliations:** † Institute of Organic Chemistry, Polish Academy of Sciences, Kasprzaka 44/52, 01-224 Warsaw, Poland; ‡ Institute of Physical Chemistry, Polish Academy of Sciences, Kasprzaka 44/52, 01-224 Warsaw, Poland; § Department of Chemistry, Łódź University of Technology, Stefana Żeromskiego 114, 90-543 Łódź, Poland

**Keywords:** PAHs, N-doping, dyes, D−A−D, functional aromatic materials, TADF, OLEDs

## Abstract

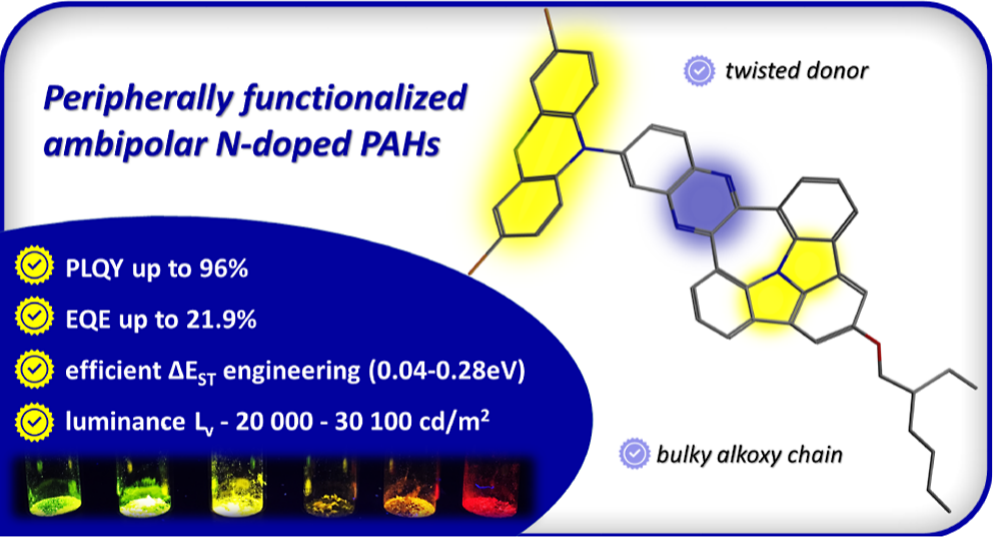

Despite promising optoelectronic features of N-doped polycyclic aromatic hydrocarbons (PAHs), their use as functional materials remains underdeveloped due to their limited post-functionalization. Facing this challenge, a novel design of N-doped PAHs with D–A–D electronic structure for thermally activated delayed fluorescence (TADF) emitters was performed. Implementing a set of auxiliary donors at the meta position of the protruding phenyl ring of quinoxaline triggers an increase in the charge-transfer property simultaneously decreasing the delayed fluorescence lifetime. This, in turn, contributes to a narrow (0.04–0.28 eV) singlet–triplet exchange energy split (Δ*E*_ST_) and promotes a reverse intersystem crossing transition that is pivotal for an efficient TADF process. Boosting the electron-donating ability of our N-PAH scaffold leads to excellent photoluminescence quantum yield that was found in a solid-state matrix up to 96% (for phenoxazine-substituted derivatives, under air) with yellow or orange-red emission, depending on the specific compound. Organic light-emitting diodes (OLEDs) utilizing six, (D–A)–D, N-PAH emitters demonstrate a significant throughput with a maximum external quantum efficiency of 21.9% which is accompanied by remarkable luminance values which were found for all investigated devices in the range of 20,000–30,100 cd/m^2^ which is the highest reported to date for N-doped PAHs investigated in the OLED domain.

## 1 Introduction

**Figure 1:**
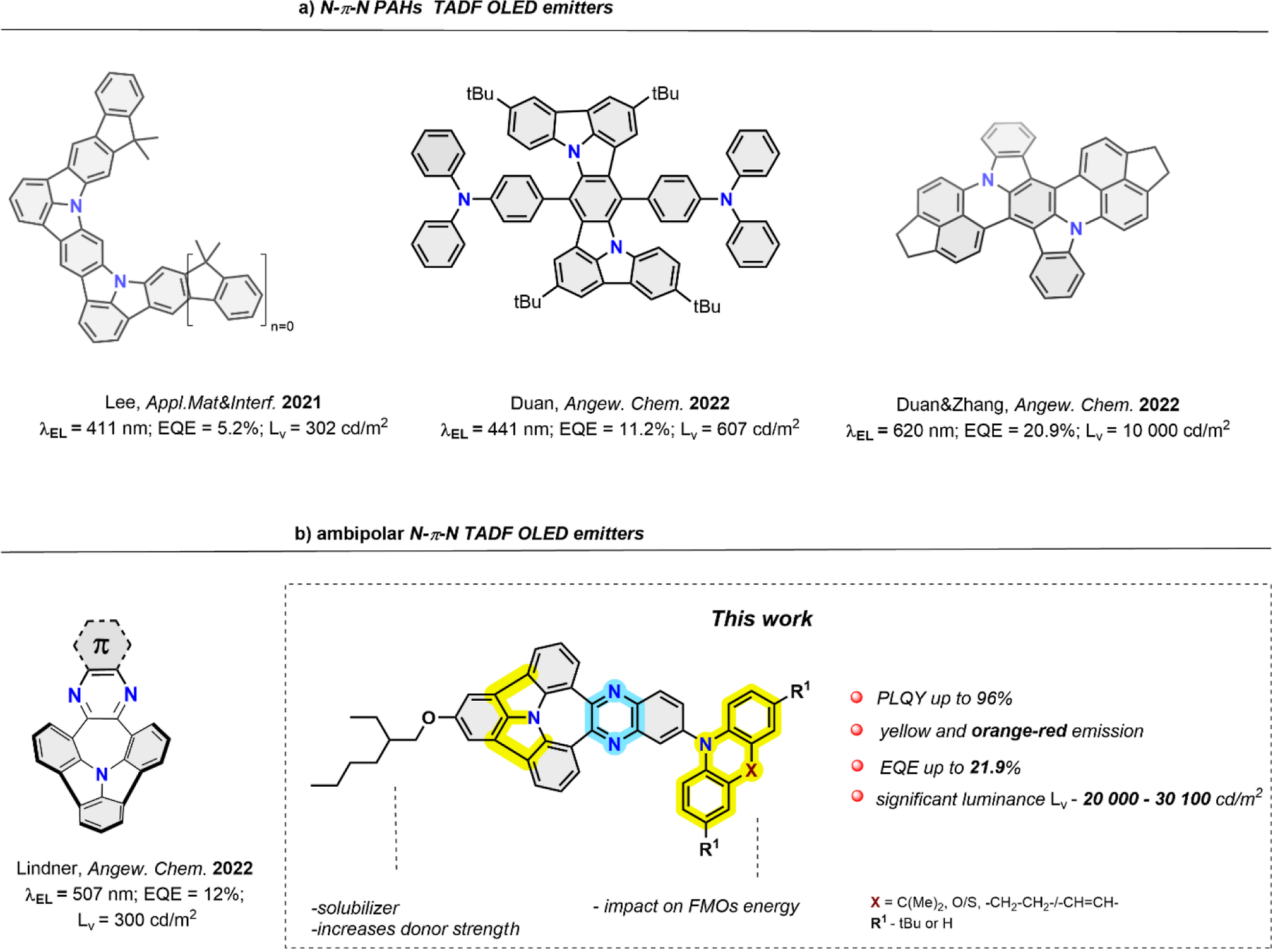
(a) Current development of N−π–N PAH-based TADF OLED emitters; (b) new approach toward N−π–N PAHs with ambipolar structures; λ_EL_—electroluminescence max.; EQE—external quantum emission; and *L*_v_—luminance.

π-Conjugated polycyclic aromatic hydrocarbons (PAHs)^1−3^ comprising precisely arranged heteroatoms have recently caused a growing interest due to their prospective application in organic electronics. In this context, much attention has been paid to PAH architectures bearing nitrogen atom(s) positioned at the central part (hub position) of aromatic scaffolds (N-PAHs).^4−14^ The synthetic integration of an N-dopant within three rings (varying from pentagons to heptagons) is anticipated to not only produce a steric constraint that would largely influence the geometry and stability of N-doped PAHs but primarily to affect energy, localization, and spatial extent of a molecular orbital (HOMO) energy level, leading to their strong electron-rich character.^15,16^ The latter features constitute the development of an innovative class of semiconducting materials that have found utility as organic field effect transistors,^17^ sensing,^16^ p-type transporting layers in perovskite solar cells,^18^ and importantly thermally activated delayed fluorescence (TADF)^19^ for organic light-emitting diodes (OLEDs). In this regard, researchers demonstrated nitrogen-embedded PAHs as eligible to cause the multiple resonance (MR) effect in which MOs are alternately distributed likewise to the chess board triggering a short-range charge-transfer (CT) TADF emission. Along this line, the synthesis of the first rationally designed emitters of this type was disclosed by Lee,^20,21^ who reported an N−π–N fused inolo[3,2,1- *jk*]carbazole (Icz) with the synergic effect of meta-positioned nitrogen atoms (Figure 1a) to delocalize excited states. Further development with N-centered atoms, surrounded by three mutually fused aromatic rings, was pursued with the para-positioned Icz and dibenzo[2,3;5,6]-pyrrolizino[1,7;*bc*]indolo[1,2,3;*lm*]carbazole, respectively (Figure 1a).^22−24^ Importantly, their non-donor–acceptor type of structure and significant contribution of N−π–N electron-rich fused moieties to the enhancement of HOMO energy result mostly in a large energy gap (*E*_g_) and pure violet and deep blue light-emitting systems. Moreover, their inherent non-bonding orbital characters as MR emitters reduce possible π-extension as a viable scenario to control the emission color. Up to date, solely one molecular design based on naphthalene and acenaphthene-decorated N−π–N PAHs demonstrated by the group of Zhang^25^ deals with orange-to-red-shifted emission. These, however, possess planar or only slightly bent structures. Accordingly, there is a large risk of an unwanted aggregation-caused, quenching effect with a consequently low and moderate roll-off process. Therefore, seeking for a more modular approach to tune the desired geometry of N−π–N PAHs simultaneously with their optical features and significant stability under operating voltage has still remained a formidable challenge.

Tackling this issue, we recently showed dibenzoazepine to be exploited as a fundamental component to assemble a curved structure because of the non-hexagonal rings and the incorporation of nitrogen. Profiting from its readily oxidizable antiaromatic ring, we were able to obtain a set of phenazine-decorated, D–A-fused N−π–N PAH architectures (Figure 1b). These bright curved dyes were, for the first time, used as yellow to orange TADF/RTP OLED with substantial photoluminescence quantum yields (PLQYs) and external quantum efficiency (EQE) as high as 12%, along with satisfactory operational stability and a low roll-off process.^26^

Building on that we envisioned a straightforward approach to enhance the CT properties of our ambipolar N-PAHs through its peripheral functionalization with electron-rich substituents (Figure 1b). Bearing an array of pending and twisted donors which are directly attached to the electron-accepting phenazine moieties, one can expect to enlarge the intermolecular distances between neighboring molecules and thus reduce the risk of π–π stacking. Consequently, the minimized highest occupied molecular orbital/lowest unoccupied molecular orbital (HOMO/LUMO) overlap causes more efficient reverse intersystem crossing (rISC) triplet exciton transitions, which is reflected in the shortened delayed fluorescence (DF) lifetime, along with remarkable PLQY (up to 95.9%). Implementation of N-PAH-based assemblies embedded with a D–A–D electronic structure allows us to successfully fabricate a series of TADF OLED devices, with electroluminescent emission color changed from yellow to orange-red and a significant boost of EQE up to 21.9% under considerable luminance response (20,000–30,100 cd/m^2^) which shed light on the high stability of the designed emitters.

## 2 Results and Discussion

### 2.1 Synthesis

Based on our last discovery,^26^ we put forward the design strategy for a peripheral extension of basic N-PAH scaffolds to form new D–A–D systems, as displayed in Figure 1a. The rationale for this modification is to induce a twist of the bulky electron-donating substituent with respect to the N-PAH moiety, which is expected to reduce spatial overlap between the HOMO and the LUMO, and thereby to reduce the Δ*E*_ST_ value. In turn, engineering a low Δ*E*_ST_ value facilitates population transfer from triplet to singlet excitons through the rISC process. The relationship between the type of donor implemented and the emissive properties are supposed to be affected with respect to the pristine structure shown in Figure 1.^26^

**Scheme 1:**
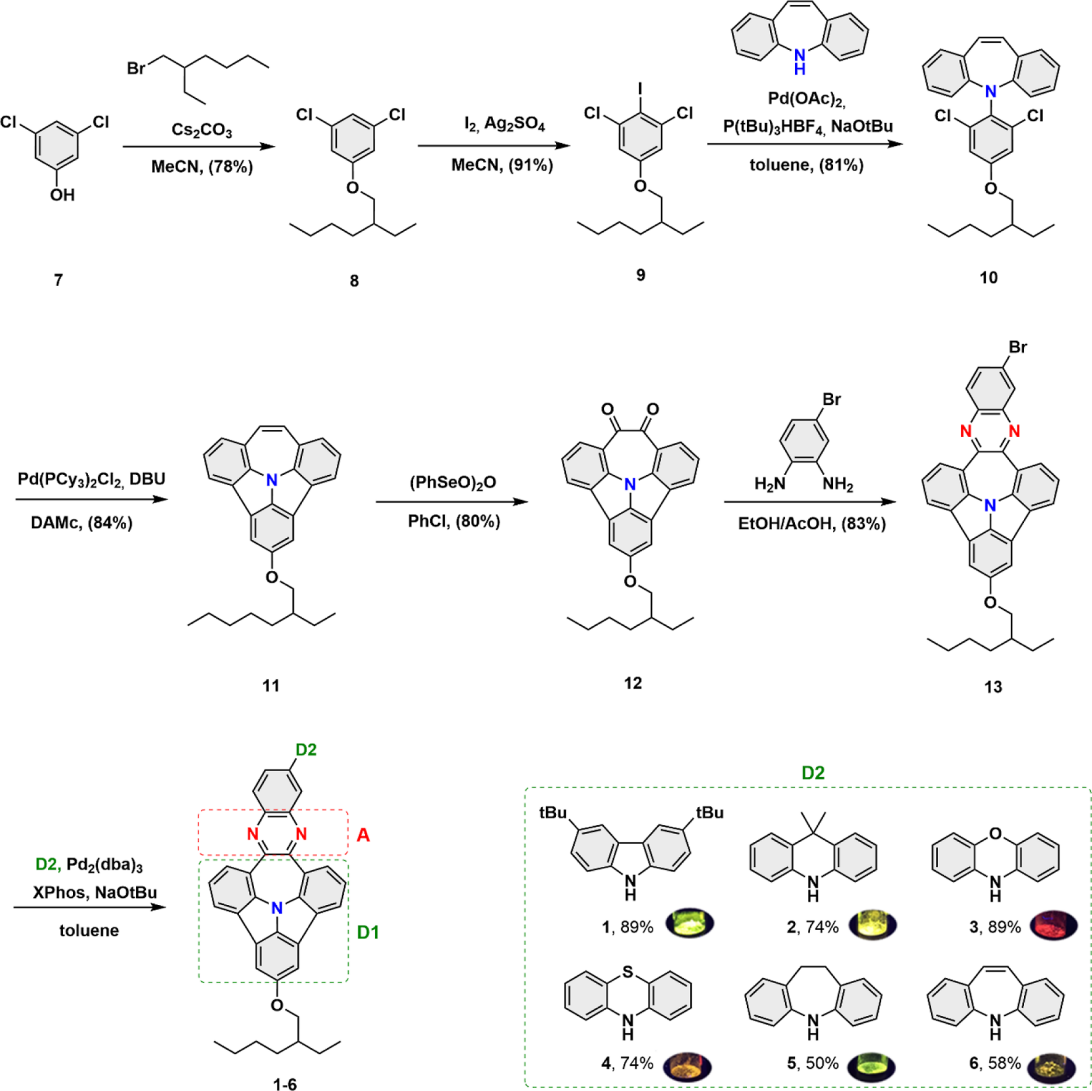
Synthetic Pathway toward N-PAH Architectures with D–A–D Electronic Structure (**1**–**6**)

Considering a moderate solubility of our parental N-PAH derivatives, the present core structure is being decorated with a long alkoxy chain to: (i) improve its processing in solution, (ii) to strengthen an electron-donating character of the segment containing the pyramidal nitrogen dopant, and (iii) to preclude aggregation-caused quenching. In these regards, a new set of emitters was assembled within scalable synthetic steps (see Scheme 1), eliminating, for the majority of transformations, a need of column chromatography separation, which is pivotal from the viewpoint of its prospective application in organic electronics. As shown in Scheme 1, the synthesis was initiated with alkylation of commercially available 3,5-dichlorophenol with 2-ethylhexylbromide providing compound **8** with good yield (78%).^27^ Next, electrophilic iodination^28^ of **8** afforded a valuable intermediate **9** (91%), which subsequently was used to perform a sequence of Pd-catalyzed steps, Buchwald–Hartwig amination (**10**, 81%), and a two-fold ring closure (**11**, 84%). An oxidation of intermediate **11** toward diketone **12** (80% yield) and its subsequent condensation with 4-bromo-1,2-diaminobenzene opened the way to conduct a series of aminations, leading to the target (D–A)–D N-PAHs (**1**–**6**) with yields ranging from 50 to 89%. The full experimental procedures for the synthesis and the characterization data of the molecules in Scheme 1 are detailed in Sections SI-2 and SI-8 of the Supporting Information.

As indicated in Scheme 1, in what follows, the fused azepine electron-donating moiety will be denoted D1, the phenazine electron-accepting moiety will be denoted A, and the pendant electron-donating moiety will be denoted D2.^26^

### 2.2 X-ray Crystallography

**Figure 2:**
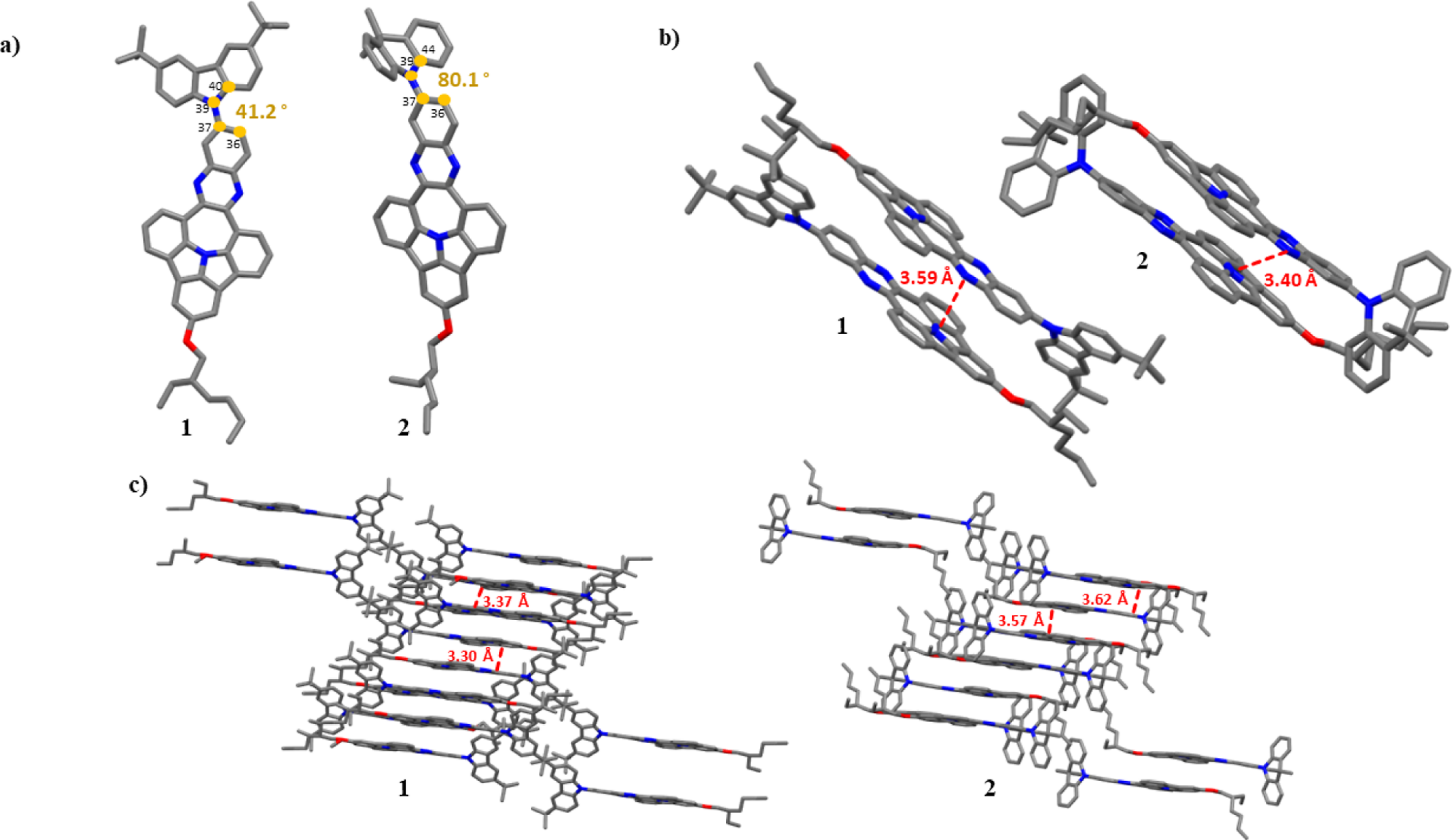
Crystal structures of **1** and **2**; (a) top view of the crystal structure of **1** and **2** with measured dihedral angles between auxiliary donors and acceptor fragments; (b,c) molecular packing in the crystalline state determined for **1** and **2**, with measured distances between adjacent molecules. Disorder solvent molecule and all hydrogen atoms were omitted for clarity.

To determine the molecular geometries of compounds **1**–**6**, we sought to determine their crystal structures. Of the six compounds, only compounds **1** and **2** formed crystals of sufficient quality for X-ray diffraction measurements. For both these compounds, crystallization was induced by the slow evaporation of solvents (chloroform for **1** and DCM in THF for **2**, both at room temperature). The crystal structures are illustrated in Figure 2a. Both compounds crystallize in the *P*1 space group. To our surprise, we found that the D1–A fragments of both compounds adopt planar geometries. This is contrary to the results of geometry optimizations for an isolated molecule of compound **1** (see the computational modeling section below), which indicate a markedly concave geometry. As this unforeseen effect severely influences the crystal packing mode, we tentatively attribute the planarization of the N-PAH fragments of compounds **1**–**6** to the molecular packing in the crystal phase. This is in contrast to the series of N-PAHs reported by our group previously,^26^ which N-PAH moieties are markedly concave. The visual comparison of both geometries (concave and planar) is shown in Supporting Information (Figure S6).

In the crystal phase, each N-PAH fragment is sandwiched between two other N-PAH fragments (per unit cell) in a head-to-tail π-stacking arrangement with the nearest distance found to be 3.59and 3.40 Å for compounds **1** and **2**, respectively (see Figure 2b).

Moreover, both molecules tend to form an extended 3D structure in a crystal lattice via two C–H···π intermolecular interactions with distances between neighboring molecules found to be 3.57 and 3.62 Å for dye **2** (Figure 2c), whereas short C–H···π contact distances, for **1**, were revealed as slightly smaller (3.30 and 3.37 Å, Figure 2c). Under these circumstances, the electron-rich tertiary amine group of each molecule is positioned close to the electron-poor phenazine moieties of the flanking molecules, which presumably leads to strong electrostatic interactions within the stack. These interactions may potentially be responsible for the planarization molecules of these in the crystal phase.^29,30^

Concomitantly, investigating a spatial organization of protruding donor subunits, with respect to the central scaffold, the dihedral angles were determined 41.2° for **1** while 80.1° for **2**. Thus, one can infer that a nearly perpendicular orientation of dimethylacridine (**2**) is anticipated to maintain slightly larger intermolecular distances. Despite this fact, both arrangements are not favorable enough to induce pronounced minimization of frontier molecular orbitals overlap. Therefore, the moderate value of Δ*E*_ST_ gap computed for **1** and **2** can be linked with their solid-state interplays.

### 2.3 Thermogravimetric Analysis

**Figure 3:**
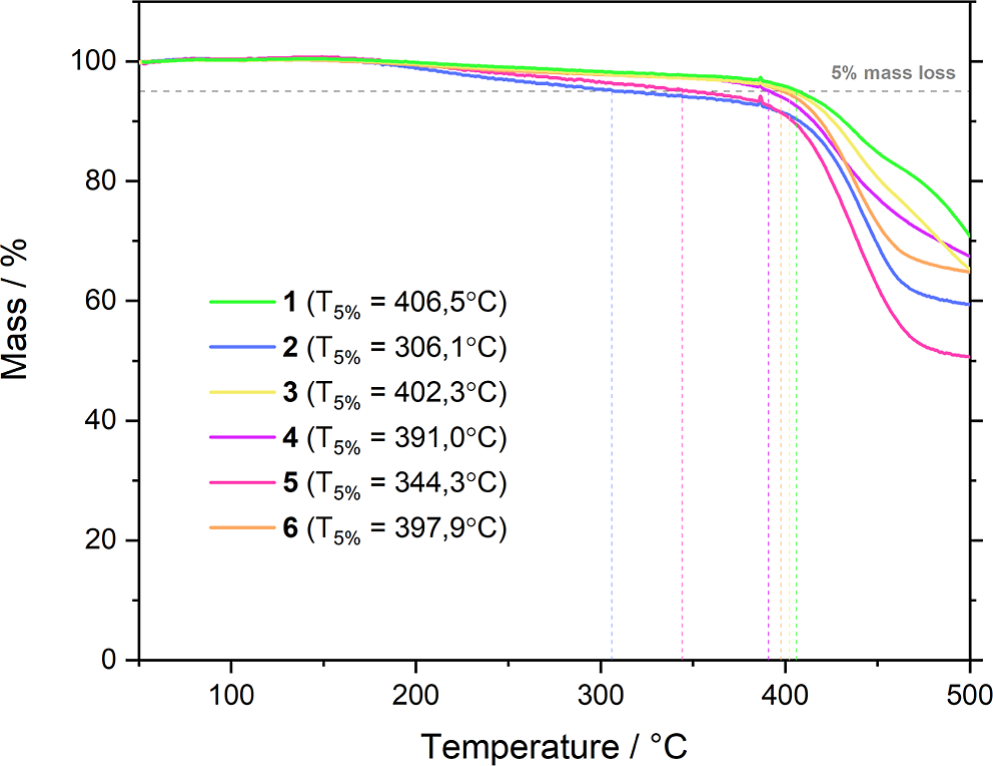
Thermogravimetric curves for the set of the dyes **1**–**6**.

As the thermal stability of emitters is a parameter which is vitally important for OLED device fabrication, thermogravimetric analysis (TGA) was performed for vacuum-sublimed samples. As demonstrated in Figure 3, the entire set of compounds exhibit much better thermal stability, with respect to parental structures,^26^ with decomposition temperatures (*T*_d_ defined as the temperature where 5% loss of initial weight is reached) in the range of 306–406 °C, which are in similar range to the parental one. As dimethylacridine derivative **2** acts in similar fashion as azepine dye **5** (*T*_d_ = 306 and 344 °C for **2** and **5**, respectively), the incorporation of a double bond to the azepine as well as an extra five- or six-membered ring, together with O and S heteroatoms to the electron-rich moiety, leads to much higher decomposition temperatures (*T*_d_ = 391–406 °C for **1, 3**, **4**, and **6**). Even that, the TG data indicates that all of the compounds **1–6** bear sufficient thermal stability to be considered as candidates for OLED fabrication. Furthermore, we conducted differential scanning calorimetry (DSC) measurement (as shown in Supporting Information Figures S13–S18). The upper-temperature limits were established based on the results of thermogravimetric measurements.

### 2.4 Computational Modeling

Prior to the spectroscopic characterization of compounds **1**–**6**, we investigated their optical properties with the use of quantum chemical calculations. Because the six compounds differ only in the choice of the pendant electron-donating moiety D2, we focused on compound **1** as a representative example for this series of compounds.

**Figure 4:**
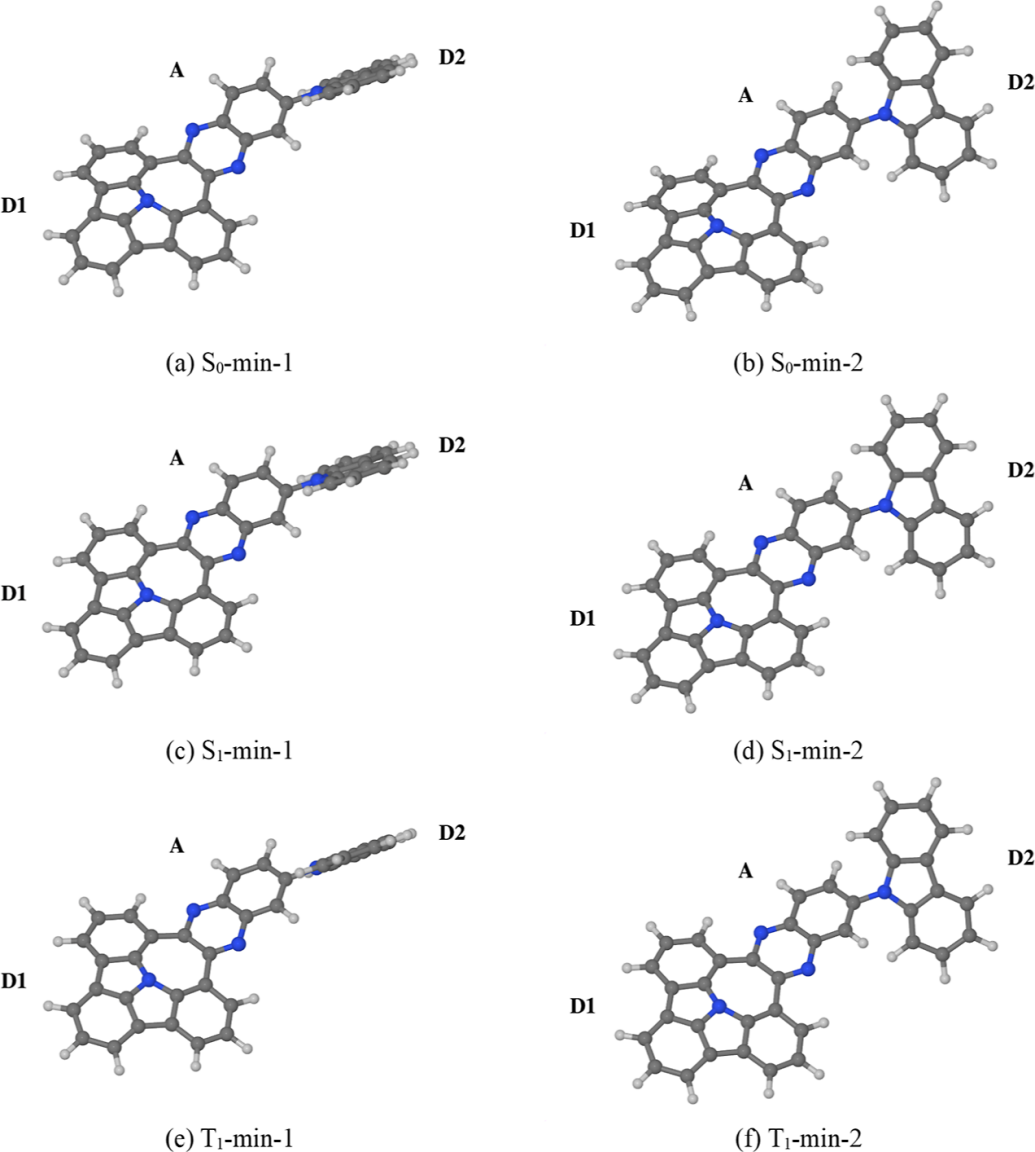
Ground- and excited-state equilibrium geometries (a–f) of **1′** as optimized at the SOS-MP2/6-31G(d) and SOS-ADC(2)/6-31G(d) levels of theory. The two electron-donating moieties are labeled D1 and D2, and the electron-accepting moiety is labeled A.

Furthermore, in our calculations, compound **1** was represented by a truncated model **1′**, in which the alkoxy group and the two *tert*-butyl groups on the D2 moiety were deleted and replaced with hydrogen atoms. We commenced the discussion on the simulation results by investigating the molecular geometry of compound **1′** in the electronic ground state. We have located two minima on the potential energy surface (PES) of the S_0_ state, which we label S_0_-min-1 and S_0_-min-2. Their geometries, which are shown in Figure 4a,b, differ mainly in the orientation of the D2 moiety with respect to the A moiety. As such, they correspond to different ground-state conformers. According to the SOS-ADC(2)/6-31G(d) level of theory, the two conformers are very close in energy, with S_0_-min-1 being marginally lower in energy (by roughly 1 meV) than S_0_-min-2. Hence, in the solution phase, the two conformers are expected to exist in about the same concentration. (NB: S_0_-min-1 and S_0_-min-2 are not a pair of enantiomers, but a pair of diastereoisomers which happen to lie very close in energy). At either ground-state minimum, the D1 moiety assumes a visibly concave (bowl-like) geometry.

**Figure 5:**
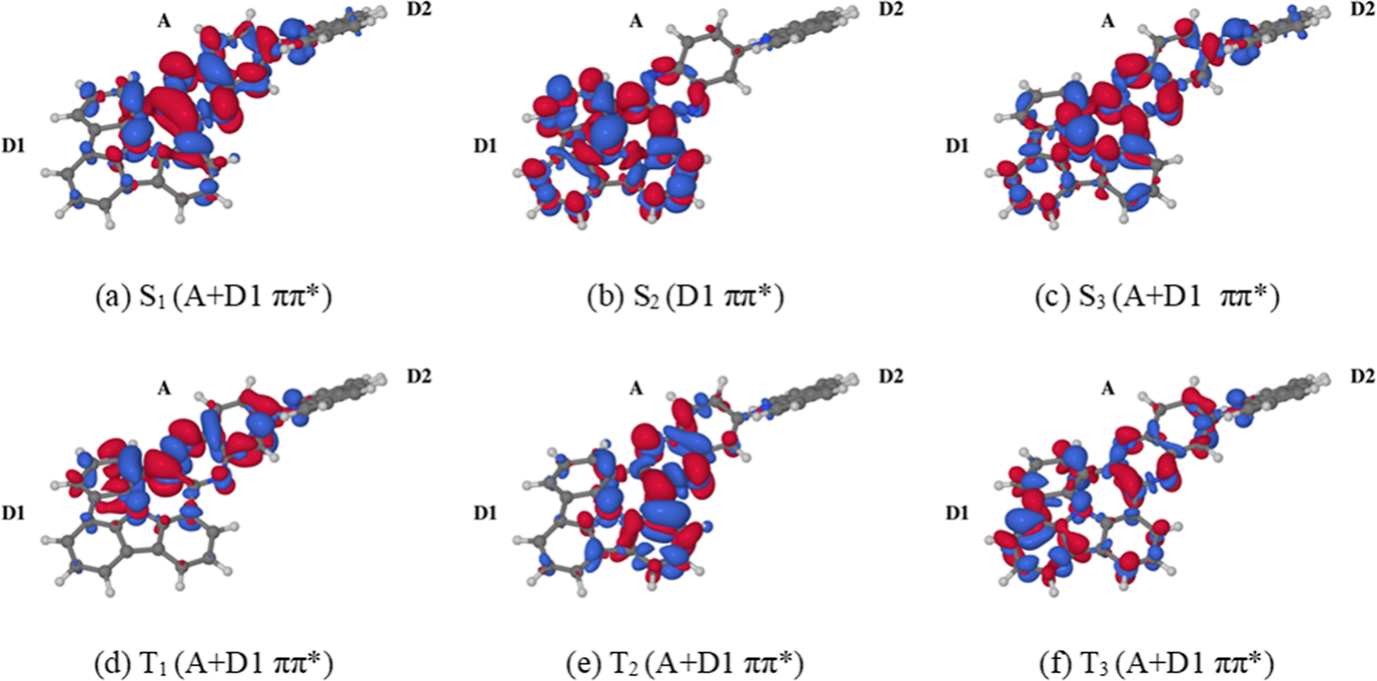
EDDMs for the low-lying excited electronic states of compound **1** calculated at the S_0_-min-1 geometry. The EDDMs for singlet (a–c) and triplet (d–f) excited states are plotted in the form of isosurfaces with isovalues of ±0.001 e/a_0_^3^. The red and blue isosurfaces delimit regions in which the electron density is increased and decreased, respectively, relative to the S_0_ state.

This is consistent with what was previously seen for the analogous compound, which lacks the pendant electron-donating group D2. Our next order of business will be the electronic excitation spectrum of compound **1′**. The calculated vertical transitions are listed in Table 1. Accompanying these data, Figure 5 shows electron density difference maps (EDDMs) of the low-lying excited electronic states of conformer S_0_-min-1 (the electronic excitation spectra of the two conformers of compound **1′** are very similar electronic excitation spectra, and so the EDDMs of conformer S_0_-min-2 need not be shown separately). As is typical of PAHs, whether or not they have been modified by doping with heteroatoms, the lowest excited electronic states are ππ*-type in character. The S_1_ state has a vertical excitation energy of roughly 3.5 eV, and it is delocalized over the A and D1 moieties. A close inspection of its EDDM (Figure 5a) reveals that the S_1_ state features a slight amount of intramolecular charge transfer (ICT) from the D1 and D2 moieties onto the A moiety. However, despite the S_1_ state having partial ICT character, its electric dipole moment is only marginally larger than that of the singlet ground state. This is presumably because the electron-donating moieties D1 and D2 are on opposite sides of the electron-accepting moiety A, such that the shift of electron density onto the latter does not lead to a significant increase of the net electric dipole moment. The S_1_ state is the only one from among the low-lying excited states of **1** to exhibit an appreciably large oscillator strength for excitation from the ground state. As such, the first photoabsorption band of compound **1** can be attributed mainly to the S_0_ → S_1_ transition. The S_1_ state is closely followed by the S_2_ state, with a vertical excitation energy of roughly 3.7 eV.

**Table 1:** Calculated Vertical Excitation Spectra of Conformers of Compound **1**′—Vertical Excitation Energies (Δ*E*) and Associated Oscillator Strengths (*f*)^a^

conformer	state	Δ*E*, eV	*f*	μ, D
S_0_-min-1	S_0_			1.6
	S_1_ (A + D1 ππ*)	3.491	0.492	2.2
	S_2_ (D1 ππ*)	3.689	0.033	2.6
	S_3_ (A + D1 ππ*)	3.998	0.021	1.0
	T_1_ (A + D1 ππ*)	3.042	0	0.9
	T_2_ (A + D1 ππ*)	3.170	0	4.5
	T_3_ (A + D1 ππ*)	3.450	0	1.1
S_0_-min-2	S_0_			1.6
	S_1_ (A + D1 ππ*)	3.495	0.490	2.4
	S_2_ (D1 ππ*)	3.689	0.033	2.6
	S_3_ (A + D1 ππ*)	3.998	0.019	1.2
	T_1_ (A + D1 ππ*)	3.046	0	0.9
	T_2_ (A + D1 ππ*)	3.168	0	4.5
	T_3_ (A + D1 ππ*)	3.450	0	1.1

a μ is the (orbital-relaxed) electric dipole moment of the given state.

**Figure 6:**
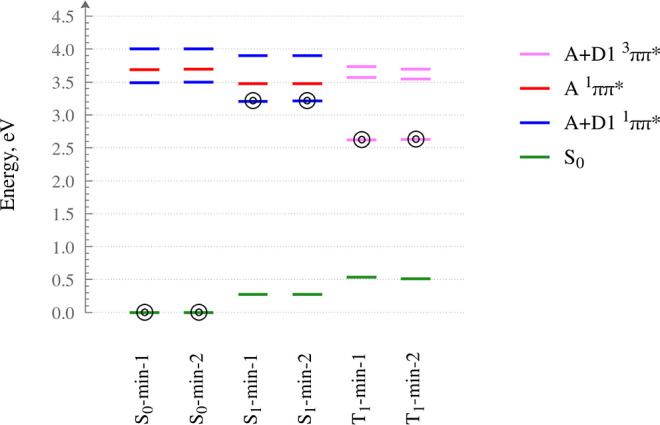
Energy-level diagram for compound **1′** as calculated at the SOS-MP2/6-31G(d) and the SOS-ADC(2)/6-31G(d) level of theory. The zero of the energy scale corresponds to the adiabatic energy of the singlet ground state (S_0_) at the S_0_-min-1 structure. The horizontal bars represent the adiabatic energies of the ground state and the lowest three excited states at each equilibrium geometry. The electronic state on which the given structure was optimized is marked with a bullseye symbol.

The S_2_ state is predominantly localized on the D1 moiety, and it does not appear to have a significant ICT character. The S_3_ state has a vertical excitation energy of around 4.0 eV, and it is delocalized over the A and the D1 moieties. As with the S_1_ state, in the S_3_ state, there is a certain amount of ICT from the D1 and D2 moieties onto the A moiety, although the electric dipole moment of the S_3_ state is still small in magnitude. Regarding, in turn, the triplet states, the three lowest triplet excited states are all delocalized over the A and D1 moieties, and they have at most a slight ICT character. Geometry optimizations on the PES of the S_1_ state reveal two minima (S_1_-min-1 and S_1_-min-2), which are essentially counterparts of the two ground-state conformers. Their geometries are shown in Figure 5c,d, respectively. Furthermore, an energy-level diagram for compound **1′** is provided in Figure 6. At either minimum on the S_1_ state, the D1 moiety assumes a near-planar geometry. Both the excited-state structures are weakly polar (specifically, S_1_-min-1 and S_1_-min-2 are calculated to have electric dipole moments of 4.3 D and 4.4 D, respectively).

This finding is consistent with the experimental observation that compound **1′** and the other compounds in the series **1**–**6** do not exhibit significant solvatofluorochromism. Compound **1′** likewise possesses two minima on the PES of the T_1_ state (T_1_-min-1 and T_1_-min-2), whose geometries are shown in Figure 5e,f, respectively. At either minimum on the T1 state, the D1 moiety is slightly concave, while the A moiety is somewhat deformed and bent away from the plane of the D1 moiety.

### 2.5 Photophysics of the Singlet and Triplet Excited States

Having examined the low-lying excited electronic states of compound **1** with model system **1′** and state-of-the-art electronic structure calculations, we are now prepared to discuss the photophysical characterization of newly synthesized emitters **1**–**6**. The compounds exhibit classical behavior in the steady-state absorption and photoluminescence (PL) analysis (Figure S1) with the emission peak in the range of 500—550 nm.

**Figure 7:**
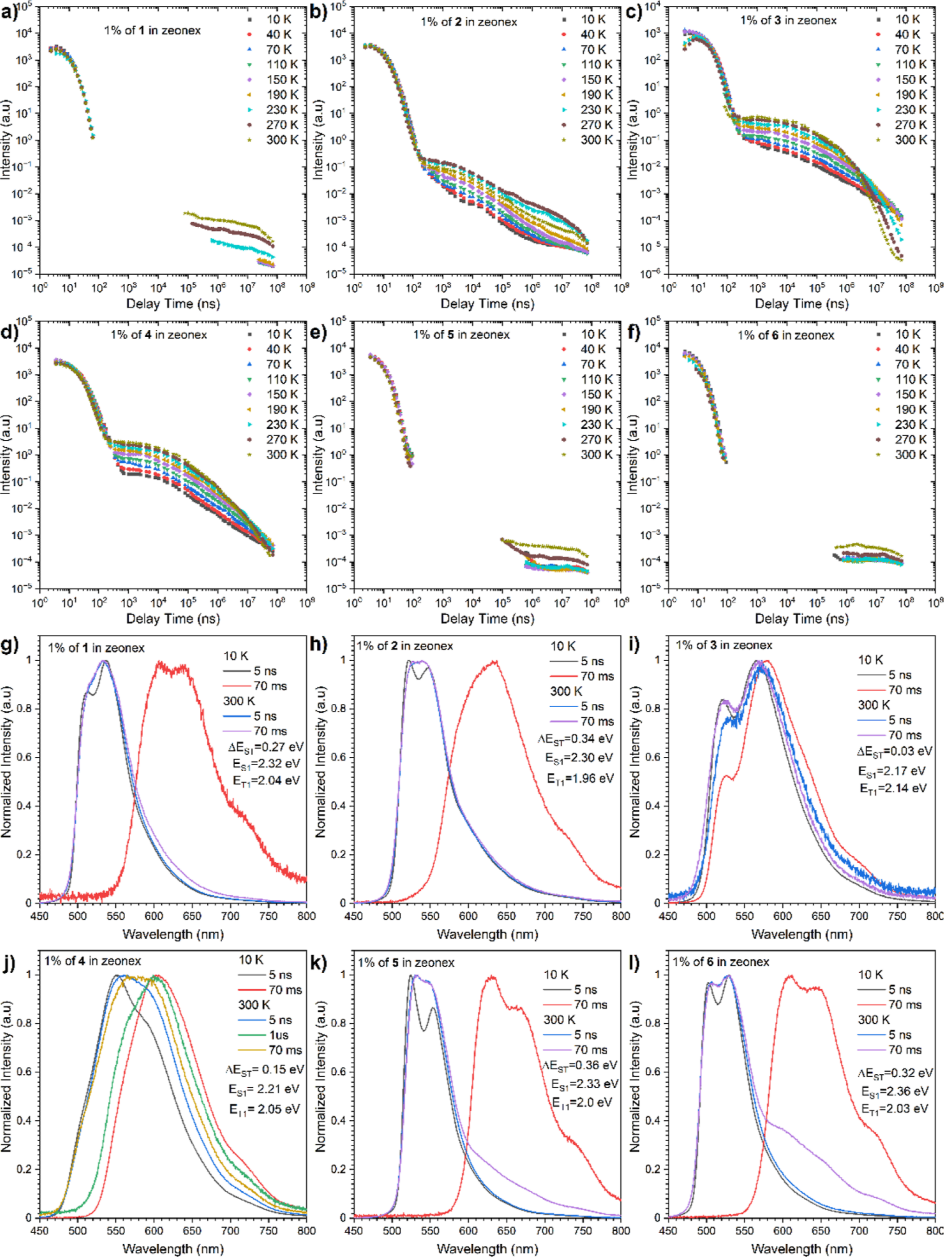
Intensity vs delay time measurement decays of compounds **1-6** in the Zeonex matrix (g–l). Time-resolved spectra (a–f), the energies correspond to the maximum emission peaks.

Additionally, the entire set of dyes show a limited solvatochromism property even the charge transfer (CT) emission is clearly observed, suggesting the emissive state is from a mixed CT&LE state. When it comes to the time-resolved emission in the solid state in two different matrixes (Zeonex, CBP), they revealed an intriguing behavior. First of all, the emission at low temperature and delayed time (70 ms) correspond to the phosphorescence emission from the LE state, where the emission in the ns time regime correspond also to the mixed LE&CT states. In terms of the carbazole (**1**) and dimethylacridine (**2**) functionalized emitters, both showed classical TADF behavior in the Zeonex matrix (Figure 7a,b,g,h), in which the compound **1** exhibited a delayed emission only at very long (millisecond) times.

**Figure 8:**
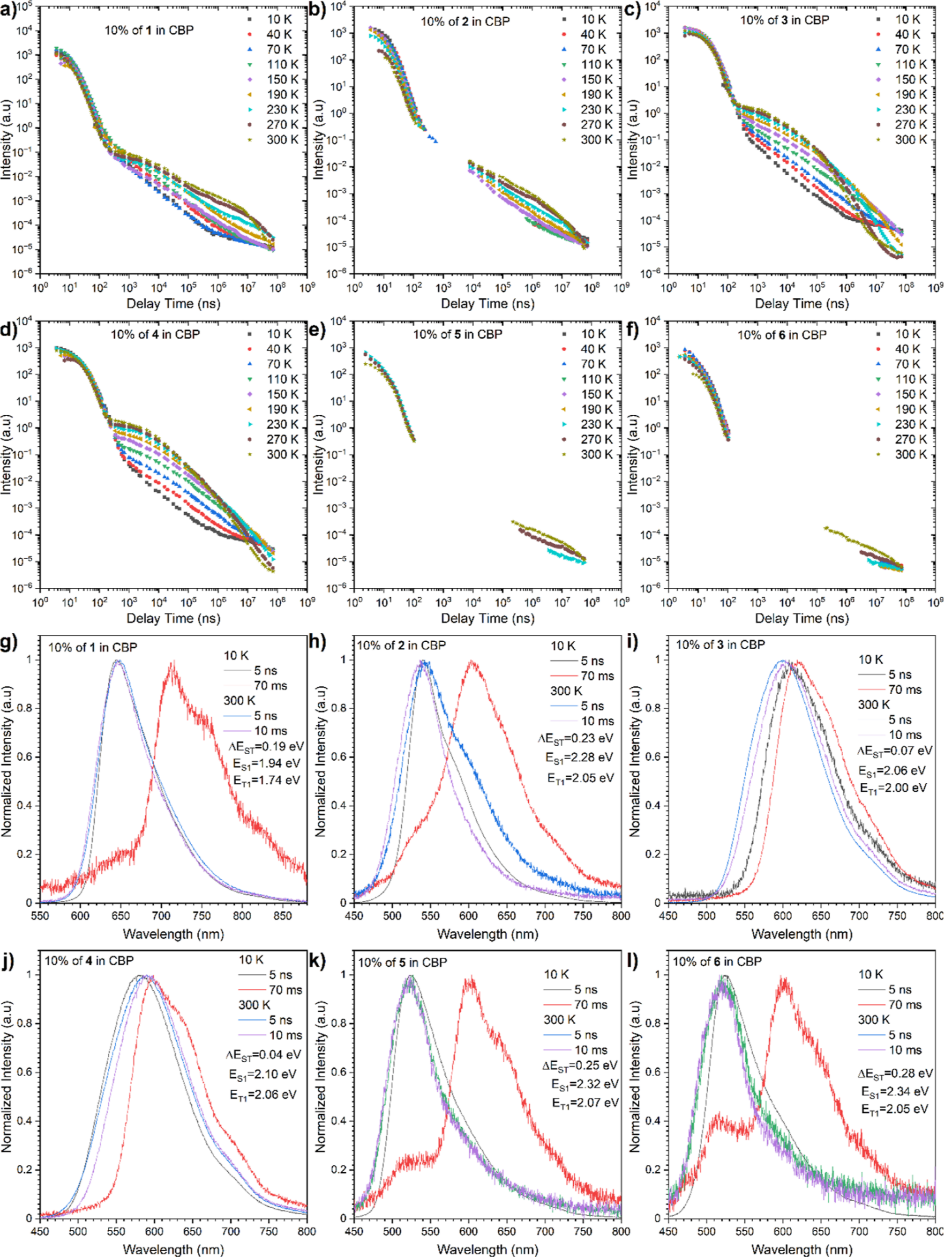
Intensity vs delay time measurement decays of compounds **1–6** in the CBP matrix (g–l). Time-resolved spectra (a–f), the energies correspond to the maximum emission peaks.

Consecutively, dyes **5** and **6** exhibited the classical TADF/RTP mechanism of emission at long delayed times (Figure 7e,f,k,l). Although most of the emission originated from the TADF mechanism, it was a visible rise of the tail emission at millisecond delay time. Surprisingly, dyes **3** and **4** behaved completely differently. For the phenoxazine derivative **3**, typical TADF mechanism with low Δ*E*_ST_ gap was observed, while for the phenothiazine analogue **4**, we found mixed emission from TADF and RTP processes (Figure 8c,d,i,j). Usually, the phenoxazine- and phenothiazine-based emitters possess similar emissive properties but in this particular case, in a Zeonex matrix, the situation is altered as those molecules tend to adopt different conformations.^31,32^ More precisely, compound **3** is quasi-equatorial, whereas phenothiazine **4** holds a quasi-axial conformation. This structural feature rationalizes the difference in emissive pathways and the observation of a RTP process. Similar behavior was observed in previous studies, where polymeric hosts allowed for quasi-axial conformation and observation of the RTP process, and it was associated with dopant and host interactions.^33−35^ In the CBP matrix, likewise to Zeonex, the phosphorescence comes from the LE state, where the fluorescence and DF are from mixed LE&CT states. Different conclusions could be extracted from the time-resolved spectra (Figure 8). Importantly, both compounds **3** and **4** exhibited TADF emissions with very low Δ*E*_ST_ gaps, which suggests the presence of quasi-equatorial conformations.

On the other hand, the high gap and mixed emissive property of compounds **5** and **6** together with their electrochemical results (Figure S3), from which the acceptor core was found to have higher LUMO energy, can suggest that both of them have quasi-axial conformations as standard which was additionally supported by quantum mechanic (QM) calculations (vide supra). This explains a long-lived DF emission with phosphorescence component and high Δ*E*_ST_ gap (Table 2). Since quasi-equatorial conformation (featured with 90° angle) was recognized for compounds **1** and **2**, their CT properties are expectably amplified,^37^ leading to the strong TADF contribution. Dyes **1** and **2** deliver interesting results as well, although the Δ*E*_ST_ gaps drop by only ca. 100 meV, leaving a moderately high difference between singlet and triplet levels (ca. 0.2 eV). Nevertheless, substantial contribution of the DF component is visible (4.64 times for compound **2**) with a high increase of the emission intensity and PLQY by oxygen removal (3.1 and 4.3 times for compounds **1** and **2**, Table 2). This behavior suggests a fast rISC process and that the DF activation energy is more important rather Δ*E*_ST_ gap.

**Table 2:** Summary of the General Photophysical Properties of Compounds **1–6**

compound	λ_em_, nm^a^	host	PLQY (%)^b^	τ_PF_, ns^c^	τ_DF_, μs^d^	DF/PF^e^	*E*_a_ eV^f^	S_1_ eV^g^	T_1_, eV^g^	DE_ST_, eV^h^	*k*_r_, 10^5^ s^–1^^i^	*k*_nr_, 10^5^ s^–1^^i^	*k*_RISC_, 10^5^ s^–1^^j^
**1**	535	Zeonex	58	6.96 ± 0.10	41.63 ± 1.61	1.41	0.11	2.32	2.04	0.28	4.1	0.10	0.34*
	550	CBP	95	6.25 ± 0.18	18.92 ± 1.14	1.93	0.05	1.94	1.74	0.19	6.7	0.03	0.75*
**2**	538	Zeonex	84	7.97 ± 0.10	1.89 ± 0.19	2.00	0.03	2.30	1.96	0.35	4.3	0.78	10.61*
	543	CBP	90	9.95 ± 0.60	8.44 ± 0.61	4.64	0.05	2.28	2.05	0.23	1.9	0.12	5.50
**3**	572	Zeonex	74	18.59 ± 0.82	29.97 ± 3.11	8.38	0.03	2.17	2.14	0.03	0.9	0.09	2.79
	600	CBP	96	16.52 ± 0.45	12.11 ± 1.18	1.81	0.04	2.07	2.00	0.07	7.2	0.03	1.10*
**4**	562	Zeonex	68	16.51 ± 0.26	5.72 ± 0.36	8.67	0.02	2.21	2.06	0.15	0.8	0.57	15.15
	590	CBP	84	17.77 ± 0.47	6.11 ± 0.16	1.96	0.04	2.11	2.07	0.04	4.3	0.27	3.21*
**5**	530	Zeonex	39	6.61 ± 46	82.17 ± 4.54	0.58	0.04	2.34	1.97	0.37	6.8	0.07	0.07*
	534	CBP	34	7.93 ± 0.23	27.48 ± 1.96	0.95	0.10	2.33	2.07	0.26	3.6	0.24	0.34*
**6**	530	Zeonex	53	6.54 ± 0.20	84.96 ± 4.53	2.53	0.09	2.35	2.03	0.32	2.1	0.06	0.30*
	530	CBP	33	12.03 ± 0.85	43.22 ± 3.22	0.92	0.08	2.34	2.06	0.28	3.5	0.16	0.21*

a Photoluminescence maximum.

b Photoluminescence quantum yield under vacuum conditions.

c Prompt fluorescence (PF) lifetime in host.

d Delayed emission lifetime in host.

e DF to PF ratio in host.

f Activation energy of the triplet to singlet transfer.

g Singlet and triplet energy in host. Error ± 0.03 eV.

h Singlet-triplet energy splitting in Zeonex. Error ± 0.05 eV.

i Estimates of *k*_r_ and *k*_nr_ assuming that the emitting state is formed with unit efficiency such that *k*_r_ = Φ/τ and *k*_nr_ = (1 – Φ)/^τ^.^36^

j Values of reverse intersystem crossing rate constant, *k*_RISC_*k*_RISC_ = (DF/PF)/τ_DF_ * Rough estimation of the rate constant values as this system does not fulfill all assumption to use abovementioned equations.^36^

### 2.6 OLED Fabrication

**Figure 9:**
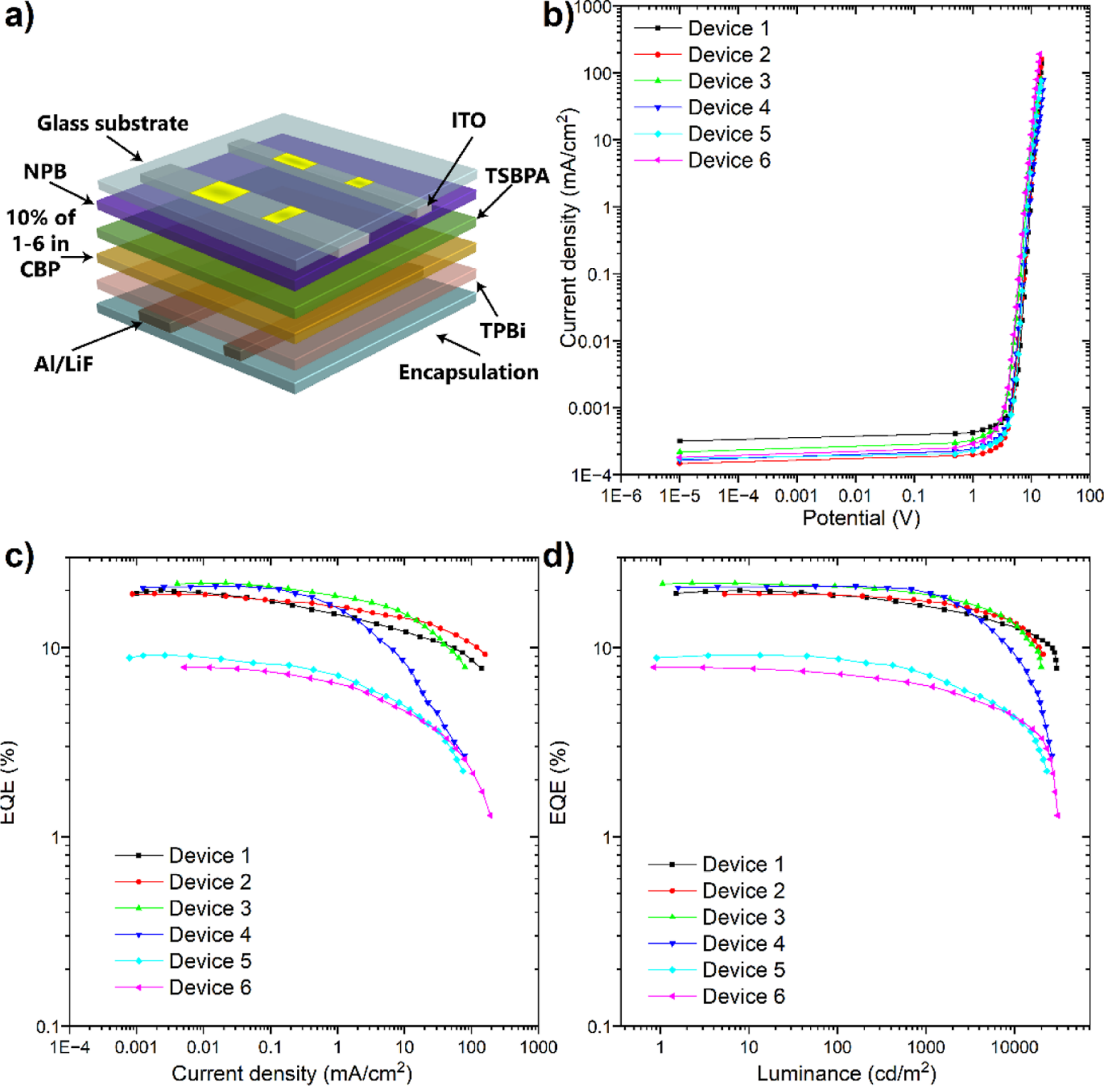
Characteristics of the OLED devices based on emitters **1–6**. (a) OLED structure. (b) Current density-bias characteristic. (c) EQE–current density characteristics. (d) EQE–luminance characteristics.

As a final step, the organic LEDs were prepared and characterized to support the efficiency obtained by photophysical analysis. Based on the electrochemical analysis for the HOMO–LUMO values and triplet levels of the emitters, the host and OLED structure were chosen. As optimal host for all compounds, the CBP host was chosen. The optimal device structure was similar to previously reported OLEDs.^26^ Devices **1–6** -ITO/NPB [*N*,*N*′-di(1-naphthyl)-*N*,*N*′-diphenyl-(1,1′-biphenyl)-4,4′-diamine] (30 nm)/TSBPA [4,4′-(diphenylsilanediyl)bis(*N*,*N*-diphenylaniline)] (10 nm)/10% of **1–6** in CBP (25 nm)/TPBi [2,2′,2″-(1,3,5- benzinetriyl)-tris(1-phenyl-1-*H*-benzimidazole)] (50 nm)/LiF (1 nm)/Al (100 nm). Schematic diagram illustrating the device structure is also presented in Figure 9a. All the OLEDs showed TADF emission, and there was no visible RTP component.

The overall OLED efficiency showed high EQE, up to 21.9% for Device **3** and 21.1% for Device **4** (Table S3), proving the overall concept true in comparison to previously studied compounds. The lowest efficiency was obtained for Devices **5** and **6** based on the quasi-axial compounds **5** and **6** down to 9.1% for Device **5** and 7.8% for Devices **6**. Nevertheless, even with low efficiency for those two compounds, they exhibit quite high values in comparison to other studies based on the same donor (D–A or D–A–D) structures. Good optimization of the OLED devices can be supported by very high luminance values which for all devices went above 20,000 cd/m^2^, with the maximum for Device **1**, based on a carbazole donor, up to 30,109 cd/m^2^ (Figure 9). The devices have very low roll-off, showing high efficiency at high luminance (Figure 9d).

## 3 Conclusions

We demonstrate a facile approach toward efficient TADF OLED emitters through peripherally functionalized ambipolar N-doped PAH. Implementation of an auxiliary electron-donating group leads to extended D–A–D electronic structure of our dyes. The twisted donor and bulky O-alkyl chain mounted at the opposite peripheries of the scaffold have contributed to the small Δ*E*_ST_, which was translated to a smooth TADF up-conversion. Implementation of our molecular design led to not only pronounce emission efficiency (PLQY = 96%) but also to achieve a significant EQE up to 21.9% with in parallel preserved luminance (up to 31,900 cd/m^2^) which is in striking contrast to preceding studies on N-doped PAHs used in OLED domains.

## 4 Experimental Section

### 4.1 Synthesis of Dyes **1–6**

The synthetic protocols and spectroscopic identification of compounds **1**–**6** are provided in Sections SI-2 and SI-8 of the Supporting Information.

### 4.2 General Remarks

All applied reagents and solvents were acquired from commercial sources and were utilized without further purification. Reaction grade solvents, i.e., CH_2_Cl_2_, ethyl acetate, and hexane were distilled prior to use. For water-sensitive reactions, solvents were dried using the swift solvent purification system by MBraun (https://www.mbraun.com/us/), while for moisture and oxygen-sensitive transformations, reactions were proceeded under an inert atmosphere of argon. The progress of the reaction was monitored by using of thin-layer chromatography (TLC), applying, silica gel-coated (60 F254 Merck) aluminum foil plates. A column chromatography purification (Kieselgel 60 Merck) was carried out to purify intermediates and target products. Characterization of all intermediates and target compounds was carried out by ^1^H NMR and ^13^C NMR spectrometry as well as by HRMS spectrometry (via EI-MS) and IR spectroscopy. Purity of compounds was corroborated with the HPLC technique (Shimadzu HPLC Chromatograph). NMR spectra were recorded with Bruker AM 500 MHz, Bruker AM 600 MHz, Varian 600 MHz, or Varian 400 MHz instruments. Tetramethylsilane (TMS) was used as an internal standard. Chemical shifts for ^1^H NMR are given in parts per million (ppm) with respect to the TMS (δ 0.00 ppm), CDCl_3_ (δ 7.26 ppm), and CD_2_Cl_2_ (δ 5.30 ppm). Chemical shifts for ^13^C NMR are expressed in ppm relative to CDCl_3_ (δ 77.16 ppm) and CD_2_Cl_2_ (δ 54.00 ppm). Data are displayed in the following order: chemical shift, multiplicity (s = singlet, d = doublet, dd = doublet of doublets, t = triplet, td = triplet of doublets, q = quartet, p = quintet, hept = septet, and m = multiplet), coupling constant (Hz), and integration. EI mass spectra were measured on an AutoSpec Premier spectrometer. IR spectra were obtained with a JASCO FT/IR-6200 spectrometer. TGAs were executed by means of a Mettler-Toledo TGA/DSC 3+ thermal gravimetric analyzer. The measurements were performed under an inert atmosphere of nitrogen, ranging from 50 to 500 °C at the heating rate of 5 °C/min. The temperature of 5 wt % and 10 wt % of mass loss was determined. DSC [heating/cooling (5 °C/ min)] experiments were performed using a Mettler-Toledo DSC 3 analyzer.

### 4.3 Photophysics

Photophysical measurements were performed in a similar way as previously reported.^34^ Namely, a Shimadzu UV-2550 spectrophotometer and a Jobin Yvon Horiba Fluoromax 3 spectrofluorometer were utilized for UV–vis spectra and steady-state emission spectra, respectively. According to the company-supplied specific calibration files for the instrument, PL spectra were calibrated for the detector efficiency. PL cuvettes (Aireka Cells, path length: 1 cm) were used for measurements in solution. The solutions of analyzed compounds in toluene were degassed through five freeze/thaw/pump cycles using a custom made degassing cell equipped with a Young tap (path length: 1 cm). Temperature-dependent experiments were conducted within a Janis Research cryostat cooled with liquid nitrogen. The PLQYs of the emitters were determined by exploiting the integrating sphere (both in solution and in solid state). The matrix-doped films were prepared on cleaned and dried sapphire disc substrates as 1 wt % of the emitter in Zeonex host. Spectra and decays of prompt fluorescence (PF), phosphorescence, and DF were measured utilizing nanosecond-gated luminescence and lifetime investigations (in the range of 400 ps to 1 s). For those experiments, a Q-Spark A50-TH-RE high energy pulsed DPSS laser (λ_em_ = 355 nm) was used as well as a Stanford Computer Optics sensitive gated iCCD camera with a sub-nanosecond resolution for detection. PF/DF time-resolved analysis was carried out by increasing gate and integration times (exponentially). Temperature-dependent experiments under vacuum were conducted within a Janis Research cryostat cooled with helium. Time-resolved spectra were recorded using a Stanford Computer Optics 4Picos iCCD camera by increasing the gate and delay times of iCCD camera (exponentially). In order to avoid overlapping, the delay and integration times are fixed at a time longer than the previous sum of delay and integration time. The recorded spectra are then integrated to indicate a proper luminescence decay profile. Each point shows the single emission spectra collected for the respective emitter.

### 4.4 Devices

The fabrication of OLED devices was performed in similar fashion described previously.^26,34^ NPB was implemented as a hole injection layer and hole transport layer, and TSBPA was applied as an electron blocking layer. TPBi was utilized as an electron transport layer. Lithium fluoride (LiF) and aluminum were used as the cathode. Organic semiconductors and aluminum were deposited at a rate of 1 Å s^–1^, and the LiF layer was deposited at 0.1 Å s^–1^. CBP was exploited as hosts for the entire set of emitting dyes. Materials used in following studies were acquired from Sigma-Aldrich or Lumtec and were purified by temperature-gradient sublimation under vacuum. OLEDs have been assembled on pre-cleaned, patterned indium-tin-oxide (ITO)-coated glass substrates with a sheet resistance of 20 Ω/sq and ITO thickness of 100 nm. The entire set of small mass compounds as well as cathode layers were thermally evaporated in a Kurt J. Lesker Nano36 evaporation system under pressure of 10^–7^ mbar without breaking the vacuum. The sizes of pixels were 4, 8, and 16 mm^2^. Each emitting layer has been constructed by co-deposition of the dopant and host at the specific rate to obtain 10% content of the emitter. The characterization of obtained devices was performed with 6-inch integrating sphere (Labsphere) inside the glovebox linked to a Source Meter Unit and Ocean Optics USB4000 spectrometer.

### 4.5 Calculations

DFT calculations were performed by means of the QChem 5.0 software package.42. The ωB97X-D functional was implemented to optimizing a geometry and to appropriately tune ωPBE for calculations of excited-state levels. Nonequilibrium polarizable continuum model (PCM) models were utilized to capture solvation effects. Further details of the calculations can be found in the Supporting Information.

## Supporting Information Available

^1^H NMR and ^13^C NMR spectra, HRMS, TGA, FT-IR, and HPLC analyses; detailed photophysical methods and spectra; computational data and coordinates; and crystallographic data **1** (CCDC 2250069) and **2** (CCDC 2250070) (PDF)

## References

[ref1] Narita A.; Wang X.-Y.; Feng X.; Müllen K. New Advances in Nanographene Chemistry. Chem. Soc. Rev. 2015, 44, 6616–6643. 10.1039/C5CS00183H. 26186682

[ref2] Stępień M.; Gońka E.; Żyła M.; Sprutta N. Heterocyclic Nanographenes and Other Polycyclic Heteroaromatic Compounds: Synthetic Routes, Properties, and Applications. Chem. Rev. 2017, 117, 3479–3716. 10.1021/acs.chemrev.6b00076. 27258218

[ref3] Borissov A.; Maurya Y. K.; Moshniaha L.; Wong W.-S.; Żyła-Karwowska M.; Stępień M. Recent Advances in Heterocyclic Nanographenes and Other Polycyclic Heteroaromatic Compounds. Chem. Rev. 2022, 122, 565–788. 10.1021/acs.chemrev.1c00449. 34850633 PMC8759089

[ref4] Krzeszewski M.; Dobrzycki Ł.; Sobolewski A. L.; Cyrański M. K.; Gryko D. T. Bowl-Shaped Pentagon- and Heptagon-Embedded Nanographene Containing a Central Pyrrolo[3,2- b ]Pyrrole Core. Angew. Chem., Int. Ed. 2021, 60, 14998–15005. 10.1002/anie.202104092. 33831270

[ref5] Krzeszewski M.; Dobrzycki Ł.; Sobolewski A. L.; Cyrański M. K.; Gryko D. T. Saddle-Shaped Aza-Nanographene with Multiple Odd-Membered Rings. Chem. Sci. 2023, 14, 2353–2360. 10.1039/D2SC05858H. 36873850 PMC9977460

[ref6] Zhou L.; Zhang G. A Nanoboat with Fused Concave N -Heterotriangulene. Angew. Chem., Int. Ed. 2020, 59, 8963–8968. 10.1002/anie.202002869. 32150655

[ref7] Song Y.; Zhang G. Effect of Fusion Manner of Concave Molecules on the Properties of Resulting Nanoboats. Org. Lett. 2021, 23, 491–496. 10.1021/acs.orglett.0c04008. 33403857

[ref8] Deng N.; Zhang G. Nitrogen-Centered Concave Molecules with Double Fused Pentagons. Org. Lett. 2019, 21, 5248–5251. 10.1021/acs.orglett.9b01861. 31247791

[ref9] Zhu G.; Song Y.; Zhang Q.; Ding W.; Chen X.; Wang Y.; Zhang G. Modulating the Properties of Buckybowls Containing Multiple Heteroatoms. Org. Chem. Front. 2021, 8, 727–735. 10.1039/D0QO01452D.

[ref10] Jiang S.; Yu Y.; Li D.; Chen Z.; He Y.; Li M.; Yang G.; Qiu W.; Yang Z.; Gan Y.; Lin J.; Ma Y.; Su S. Sulfone-Embedded Heterocyclic Narrowband Emitters with Strengthened Molecular Rigidity and Suppressed High-Frequency Vibronic Coupling. Angew. Chem., Int. Ed. 2023, 62, e202218892 10.1002/anie.202218892. 36815469

[ref11] Nakamura K.; Ochiai K.; Yubuta A.; He D.; Miyajima D.; Ito S. Pyridine-Fused Azacorannulene: Fine-Tuning of the Structure and Properties of Nitrogen-Embedded Buckybowls. Precis. Chem. 2023, 1, 29–33. 10.1021/prechem.3c00004. PMC12381888 40880624

[ref12] Ito S.; Tokimaru Y.; Nozaki K. Benzene-Fused Azacorannulene Bearing an Internal Nitrogen Atom. Angew. Chem., Int. Ed. 2015, 54, 7256–7260. 10.1002/anie.201502599. 25914254

[ref13] Zhang X.; Mackinnon M. R.; Bodwell G. J.; Ito S. Synthesis of a Π-Extended Azacorannulenophane Enabled by Strain-Induced 1,3-Dipolar Cycloaddition. Angew. Chem., Int. Ed. 2022, 61, e202116585 10.1002/anie.202116585. 35148448

[ref14] Li Q.; Hamamoto Y.; Kwek G.; Xing B.; Li Y.; Ito S. Diazapentabenzocorannulenium: A Hydrophilic/Biophilic Cationic Buckybowl. Angew. Chem., Int. Ed. 2022, 61, e202112638 10.1002/anie.202112638. 34863045

[ref15] Schaub T. A.; Padberg K.; Kivala M. Bridged Triarylboranes, -silanes, -amines, and -phosphines as Minimalistic Heteroatom-containing Polycyclic Aromatic Hydrocarbons: Progress and Challenges. J. Phys. Org. Chem. 2020, 33, e4022 10.1002/poc.4022.

[ref16] Hirai M.; Tanaka N.; Sakai M.; Yamaguchi S. Structurally Constrained Boron-Nitrogen-Silicon-and Phosphorus-Centered Polycyclic π-Conjugated Systems. Chem. Rev. 2019, 119, 8291–8331. 10.1021/acs.chemrev.8b00637. 30860363

[ref17] Wang C.; Dong H.; Hu W.; Liu Y.; Zhu D. Semiconducting π-Conjugated Systems in Field-Effect Transistors: A Material Odyssey of Organic Electronics. Chem. Rev. 2012, 112, 2208–2267. 10.1021/cr100380z. 22111507

[ref18] Aumaitre C.; Morin J. Polycyclic Aromatic Hydrocarbons as Potential Building Blocks for Organic Solar Cells. Chem. Rec. 2019, 19, 1142–1154. 10.1002/tcr.201900016. 31106986

[ref19] Zou S.-N.; Peng C.-C.; Yang S.-Y.; Qu Y.-K.; Yu Y.-J.; Chen X.; Jiang Z.-Q.; Liao L.-S. Fully Bridged Triphenylamine Derivatives as Color-Tunable Thermally Activated Delayed Fluorescence Emitters. Org. Lett. 2021, 23, 958–962. 10.1021/acs.orglett.0c04159. 33439028

[ref20] Lee H. L.; Chung W. J.; Lee J. Y. Narrowband and Pure Violet Organic Emitter with a Full Width at Half Maximum of 14 Nm and y Color Coordinate of Below 0.02. Small 2020, 16, 1907569 10.1002/smll.201907569. 32162765

[ref21] Patil V. V.; Lim J.; Lee J. Y. Strategic Synchronization of 7,7-Dimethyl-5,7-Dihydroindeno[2,1- b ]Carbazole for Narrow-Band, Pure Violet Organic Light-Emitting Diodes with an Efficiency of > 5% and a CIE y Coordinate of < 0.03. ACS Appl. Mater. Interfaces 2021, 13, 14440–14446. 10.1021/acsami.1c02635. 33749250

[ref22] Wei J.; Zhang C.; Zhang D.; Zhang Y.; Liu Z.; Li Z.; Yu G.; Duan L. Indolo[3,2,1- Jk ]Carbazole Embedded Multiple-Resonance Fluorophors for Narrowband Deep-blue Electroluminescence with EQE≈34.7 % and CIE _y_ ≈0.085. Angew. Chem., Int. Ed. 2021, 60, 12269–12273. 10.1002/anie.202017328. 33742743

[ref23] Meng G.; Zhang D.; Wei J.; Zhang Y.; Huang T.; Liu Z.; Yin C.; Hong X.; Wang X.; Zeng X.; Yang D.; Ma D.; Li G.; Duan L. Highly Efficient and Stable Deep-Blue OLEDs Based on Narrowband Emitters Featuring an Orthogonal Spiro-Configured Indolo[3,2,1- de ]Acridine Structure. Chem. Sci. 2022, 13, 5622–5630. 10.1039/D2SC01543A. 35694343 PMC9116299

[ref24] Hall D.; Stavrou K.; Duda E.; Danos A.; Bagnich S.; Warriner S.; Slawin A. M. Z.; Beljonne D.; Köhler A.; Monkman A.; Olivier Y.; Zysman-Colman E. Diindolocarbazole – Achieving Multiresonant Thermally Activated Delayed Fluorescence without the Need for Acceptor Units. Mater. Horiz. 2022, 9, 1068–1080. 10.1039/D1MH01383A. 35067689

[ref25] Zeng X.; Wang X.; Zhang Y.; Meng G.; Wei J.; Liu Z.; Jia X.; Li G.; Duan L.; Zhang D. Nitrogen-Embedded Multi-Resonance Heteroaromatics with Prolonged Homogeneous Hexatomic Rings. Angew. Chem., Int. Ed. 2022, 61, e202117181 10.1002/anie.202117181. 35092123

[ref26] Wagner J.; Zimmermann Crocomo P.; Kochman M. A.; Kubas A.; Data P.; Lindner M. Modular Nitrogen-Doped Concave Polycyclic Aromatic Hydrocarbons for High-Performance Organic Light-Emitting Diodes with Tunable Emission Mechanisms**. Angew. Chem., Int. Ed. 2022, 61, e202202232 10.1002/anie.202202232. PMC9321062 35348258

[ref27] Bender A. M.; Griggs N. W.; Gao C.; Trask T. J.; Traynor J. R.; Mosberg H. I. Rapid Synthesis of Boc-2′,6′-dimethyl-l-tyrosine and Derivatives and Incorporation into Opioid Peptidomimetics. ACS Med. Chem. Lett. 2015, 6, 1199–1203. 10.1021/acsmedchemlett.5b00344. 26713104 PMC4677371

[ref28] Patel D. G. D.; Ohnishi Y.; Yang Y.; Eom S.-H.; Farley R. T.; Graham K. R.; Xue J.; Hirata S.; Schanze K. S.; Reynolds J. R. Conjugated Polymers for Pure UV Light Emission: Poly(Meta -Phenylenes). J. Polym. Sci. B Polym. Phys. 2011, 49, 557–565. 10.1002/polb.22224.

[ref29] Kroeger A. A.; Karton A. Graphene-induced Planarization of Cyclooctatetraene Derivatives. J. Comput. Chem. 2022, 43, 96–105. 10.1002/jcc.26774. 34677827

[ref30] Karton A. Planarization of Negatively Curved [7]Circulene on a Graphene Monolayer. Chem. Phys. 2023, 569, 111853 10.1016/j.chemphys.2023.111853.

[ref31] Wang K.; Zheng C.-J.; Liu W.; Liang K.; Shi Y.-Z.; Tao S.-L.; Lee C.-S.; Ou X.-M.; Zhang X.-H. Avoiding Energy Loss on TADF Emitters: Controlling the Dual Conformations of D-A Structure Molecules Based on the Pseudoplanar Segments. Adv. Mater. 2017, 29, 1701476 10.1002/adma.201701476. 29116652

[ref32] Shi Y.; Wang K.; Zhang S.; Fan X.; Tsuchiya Y.; Lee Y.; Dai G.; Chen J.; Zheng C.; Xiong S.; Ou X.; Yu J.; Jie J.; Lee C.; Adachi C.; Zhang X. Characterizing the Conformational Distribution in an Amorphous Film of an Organic Emitter and Its Application in a “Self-Doping” Organic Light-Emitting Diode. Angew. Chem., Int. Ed. 2021, 60, 25878–25883. 10.1002/anie.202108943. 34585471

[ref33] Nobuyasu R. S.; Ward J. S.; Gibson J.; Laidlaw B. A.; Ren Z.; Data P.; Batsanov A. S.; Penfold T. J.; Bryce M. R.; Dias F. B. The Influence of Molecular Geometry on the Efficiency of Thermally Activated Delayed Fluorescence. J. Mater. Chem. C 2019, 7, 6672–6684. 10.1039/C9TC00720B.

[ref34] Higginbotham H. F.; Okazaki M.; de Silva P.; Minakata S.; Takeda Y.; Data P. Heavy-Atom-Free Room-Temperature Phosphorescent Organic Light-Emitting Diodes Enabled by Excited States Engineering. ACS Appl. Mater. Interfaces 2021, 13, 2899–2907. 10.1021/acsami.0c17295. 33404215

[ref35] Stavrou K.; Franca L. G.; Böhmer T.; Duben L. M.; Marian C. M.; Monkman A. P. Unexpected Quasi-Axial Conformer in Thermally Activated Delayed Fluorescence DMAC-TRZ, Pushing Green OLEDs to Blue. Adv. Funct. Mater. 2023, 33, 2300910 10.1002/adfm.202300910.

[ref36] Dias F. B.; Penfold T. J.; Monkman A. P. Photophysics of Thermally Activated Delayed Fluorescence Molecules. Methods Appl. Fluoresc. 2017, 5, 012001 10.1088/2050-6120/aa537e. 28276340

[ref37] Data P.; Takeda Y. Recent Advancements in and the Future of Organic Emitters: TADF- and RTP-Active Multifunctional Organic Materials. Chem.—Asian J. 2019, 14, 1613–1636. 10.1002/asia.201801791. 30609306 PMC6590235

