# Supplementary material for: Facile Functionalization of Ambipolar, Nitrogen-Doped PAHs toward Highly Efficient TADF OLED Emitters
Source: ACS Appl Mater Interfaces. 2023 Jul 28;15(31):37728–40. doi: 10.1021/acsami.3c07552 (PMC10416149; doi:10.1021/acsami.3c07552)
Supplement: Supplementary file 1 — am3c07552_si_001.pdf [file am3c07552_si_001.pdf]

Supporting Information

Facile functionalization of ambipolar, nitrogen-doped PAHs towards highly efficient TADF OLED emitters

[a]Jakub Wagner‡, [c]Dharmendra Kumar‡, [b]Michał Andrzej Kochman‡, [b]Tomasz Gryber, [a]Magdalena Grzelak, [b]Adam Kubas, [c]Przemysław Data,* [a]Marcin Lindner**

[a]Institute of Organic Chemistry, Polish Academy of Sciences, Kasprzaka 44/52, 01-224 Warsaw, Poland;

[b]Institute of Physical Chemistry, Polish Academy of Sciences, Kasprzaka 44/52, 01-224 Warsaw, Poland;

[c]Łódź University of Technology, Department of Chemistry, Stefana Żeromskiego 114, Łódź 90-543, Poland

‡ these authors contributed equally

Corresponding Author:

* Marcin Lindner - https://orcid.org/0000-0002-5514-674X; marcin.lindner@icho.edu.pl

SI-1 Experimental section 3

SI-2 Synthetic procedures 5

SI-3 Steady-state characterisation 13

SI-5 X-Ray crystallography analysis 16

SI-6 TGA/DSC and DSC (heating/cooling) measurements 23

SI-7 OLED devices 29

SI-8 NMR spectra of synthesized compounds 30

SI-9 HPLC spectra of final compounds 42

SI-10 Molecular geometries 45

SI-11 References 54

SI-1 Experimental section

General Remarks. All reagents and solvents were purchased from commercial sources and were used as received unless otherwise noted. Reagent grade solvents (CH_2Cl_2, hexane, ethyl acetate) were distilled prior to use. For water-sensitive reactions solvents were dried using Solvent Purification System from MBraun (https://www.mbraun.com/us/). Transformations with moisture and oxygen sensitive compounds were performed under a stream of argon. The reaction progress was monitored by means of thin layer chromatography (TLC), which was performed on aluminium foil plates, covered with Silica gel 60 F254 (Merck) or Aluminium oxide 60 F254 (neutral, Merck). Products purification was done by means of column chromatography with Kieselgel 60 (Merck) or Aluminium oxide (Fluka). The identity and purity of prepared compounds were proved by ^{1}H NMR and ^{13}C NMR spectrometry as well as by HRMS spectrometry (via EI-MS) and IR spectroscopy. NMR spectra were measured on Bruker AM 500 MHz, Bruker AM 600 MHz, Varian 600 MHz or Varian 400 MHz instruments with TMS as internal standard. Chemical shifts for ^{1}H NMR are expressed in parts per million (ppm) relative to tetramethylsilane (δ 0.00 ppm), $CDCl_3$ (δ 7.26 ppm), CD_2Cl_2 (δ 5.30 ppm). Chemical shifts for ^{13}C NMR are expressed in ppm relative to $CDCl_3$ (δ 77.16 ppm), CD_2Cl_2 (δ 54.00 ppm). Data are reported as follows: chemical shift, multiplicity (s = singlet, d = doublet, dd = doublet of doublets, t = triplet, td = triplet of doublets, q = quartet, p =quintet, hept=septet, m = multiplet), coupling constant (Hz), and integration. EI mass spectra were obtained on AutoSpec Premier spectrometer. IR spectra were recorded on JASCO FT/IR-6200 spectrometer. Thermogravimetric Analyses (TGA) were performed using a Mettler-Toledo TGA/DSC 3+ thermal gravimetric analyzer. The measurements were conducted in nitrogen atmosphere, from 50°C to 500°C at the heating rate of 5°C/min. The temperature of 5 wt% and 10wt% of mass loss were determined. DSC (heating/cooling (5ºC/ min)) experiments were performed using Mettler-Toledo DSC 3 analyzer.

Photophysics. UV-vis spectra were recorded on a Shimadzu UV-2550 spectrophotometer. Steady-state emission spectra were recorded on Jobin Yvon Horiba Fluoromax 3, with solvent studies performed in clean 1 cm path-length photoluminescence cuvettes (Arieka Cells) and temperature dependent film photoluminescence films studies performed on within a liquid N_2 cooled cryostat (Janis Research). Photoluminescence spectra were calibrated for detector efficiency using company supplied, instrument specific calibration files. The emitter materials

was also degassed in toluene solvent using a custom made 1 cm path-length degassed cell stoppered with a Young tap and degassed using 5 freeze/thaw/pump cycles. The photoluminescence quantum yield (PLQY) of emitters in solvent and in solid state was determined integrating sphere. Solid-state samples were prepared as 1% w/w ratio emitters in Zeonex® polymer host on clean/dry sapphire disc substrates. Phosphorescence, prompt fluorescence (PF), and delayed fluorescence (DF) spectra and decays were recorded using nanosecond gated luminescence and lifetime measurements (from 400 ps to 1 s) using either third harmonics of a high energy pulsed DPSS laser emitting at 355 nm (Q-Spark A50-TH-RE). Emission was focused onto a spectrograph and detected on a sensitive gated iCCD camera (Stanford Computer Optics) having a sub-nanosecond resolution. PF/DF time-resolved measurements were performed by exponentially increasing gate and integration times. Temperature-dependent experiments were conducted using an helium cryostat (Janis Research) under a vacuum. Time-resolved measurements were performed by exponentially increasing the gate and delay times of iCCD Stanford Computer Optics 4Picos camera from laser excitation. The delay and integration times are set at a time longer than the previous sum of delay and integration time to avoid overlap. As the next step, the curve is corrected by integrating the measured spectra to obtain proper luminescence decay profile. Every point represents the collected emission spectra of respective emitting specie.

Devices. NPB (*N*,*N*′-di(1-naphthyl)-*N*,*N*′-diphenyl-(1,1′-biphenyl)-4,4′-diamine) was used as a Hole Injection Layer (HIL) and Hole Transport Layer (HTL), TSBPA (4,4'-(Diphenylsilanediyl)bis(N,N-diphenylaniline)) was used as a Electron Blocking Layer (EBL). TPBi 2,2’,2”-(1,3,5-Benzinetriyl)-tris(1-phenyl-1-H-benzimidazole) was introduced as an Electron Transport Layer (ETL). Lithium fluoride (LiF) and aluminium were used as the cathode. Organic semiconductors and aluminium were deposited at a rate of 1 Ås-1, and the LiF layer was deposited at 0.1 Ås-1. CBP 4,4′-bis(*N*-carbazolyl)-1,1′-biphenyl was used as hosts for all emitters. All materials were purchased from Sigma Aldrich or Lumtec and were purified by temperature-gradient sublimation in a vacuum. OLEDs have been fabricated on pre-cleaned, patterned indium-tin-oxide (ITO) coated glass substrates with a sheet resistance of 20 Ω/sq and ITO thickness of 100 nm. All small molecules and cathode layers were thermally evaporated in a Kurt J. Lesker Nano36 evaporation system under pressure of 10^{-7} mbar without breaking the vacuum. The sizes of pixels were 4 mm^2, 8 mm^2 and 16 mm^2. Each emitting layer has been formed by co-deposition of dopant and host at the specific rate to obtain 10% content

of the emitter. The characteristics of the devices were recorded using a 6-inch integrating sphere (Labsphere) inside the glovebox connected to a Source Meter Unit and Ocean Optics USB4000 spectrometer.

SI-2 Synthetic procedures

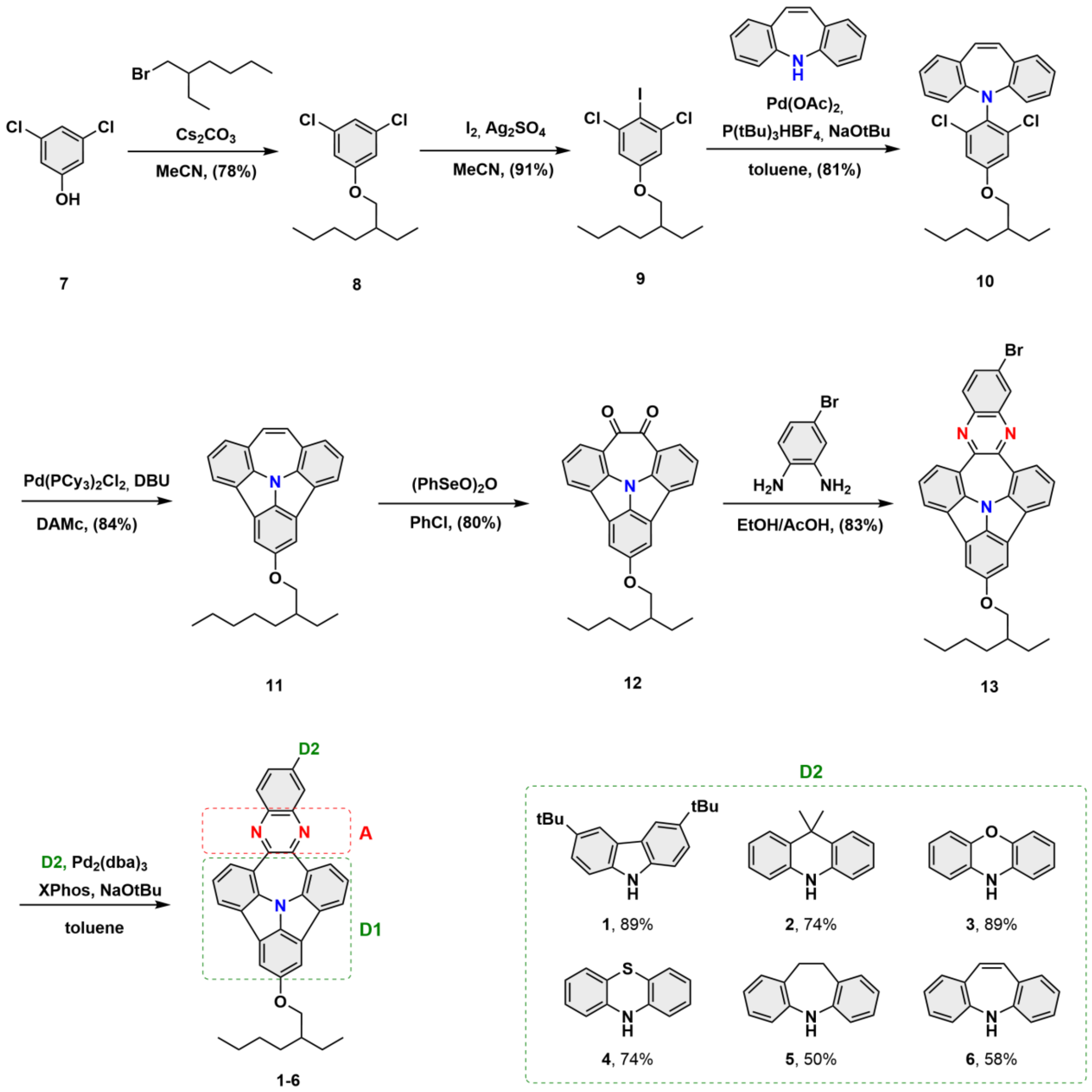

Scheme S1. The synthetic pathway towards N-PAHs with D-A-D electronic stucture.

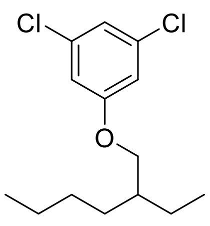

8

General procedure for the synthesis of 1,3-Dichloro-5-(2-ethylhexyloxy)benzene (8):

The compound **8** was synthesized using literature procedure. [1] 3,5-Dichlorophenol (5.00 g, 30.7 mmol, 1.0 equiv.) and cesium carbonate (20.0 g, 61.4 mmol, 2.0 equiv.) were added to an oven-dried and argon flushed 100 mL round-bottom flask equipped with magnetic stir bar, following by dry acetonitrile (40 mL). The mixture was stirred at room temperature for 30 minutes. Afterwards, 2-ethylhexylbromide (5.45 mL, 30.5 mmol, 0.99 equiv.) was added in one portion via syringe. The reaction mixture was stirred overnight at 60°C under inert atmosphere. Then, the solvent was removed under vacuum and the residue was diluted with diethyl ether. The organic layer was washed 3 times with water and dried over anhydrous Na_2SO_4. Diethyl ether was removed under vacuum and the crude mixture was passed through silica pad in hexane affording compound **8** (6.60 g, 78%) as a colourless oil. ^{1}H NMR (400 MHz, 300 K, $CDCl_3$) δ 6.93 (t, *J* = 1.8 Hz, 1H), 6.79 (d, *J* = 1.8 Hz, 2H), 3.81 (dd, *J* = 5.7, 1.2 Hz, 2H), 1.70 (m, 1H), 1.49 – 1.29 (m, 8H), 0.92 (m, 6H). (comparable to [36])

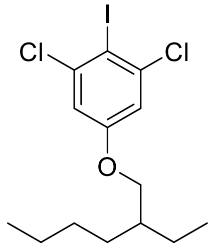

9

General procedure for the synthesis of 1,3-Dichloro-2-iodo-5-(2-ethylhexyloxy)benzene (9):

The compound **9** was synthesized using literature procedure for a similar compound. [2] The solution of 1,3-Dichloro-5-(2-ethylhexyloxy)benzene (**8**) (6.60 g, 24.0 mmol, 1.0 equiv.) in dry acetonitrile (200mL) were prepared in an oven-dried and argon flushed 500 mL round-bottom flask equipped with magnetic stir bar. Ag_2SO_4 (11.0 g, 36.0 mmol, 1.5 equiv.) and I_2 (6.64 g, 26.4 mmol, 1.1 equiv.) were added to a stirring solution. The reaction mixture was stirred for 3 days at room temperature under inert atmosphere. Then, the solvent was removed under vacuum and the residue was diluted with DCM. Saturated sodium thiosulfate solution was added and the aqueous layer was extracted 3 times with DCM. Combined organic layers were washed with brine and dried over anhydrous Na_2SO_4.Then, After cooling the reaction mixture to the room temperature, brine was addended and the mixture was extracted 3 times with DCM. The organic layers were combined and dried over anhydrous Na_2SO_4. Organic solvents were removed under vacuum and the crude mixture was passed through silica pad in hexane affording compound **9** (8.75 g, 91%) as a colourless oil. The product was slightly contaminated acc. to NMR (less than 10%) and was used in the next step without further purification. ^{1}H NMR (400 MHz, 300 K, $CDCl_3$) δ 6.96 (s, 2H), 3.80 (dd, *J* = 5.7, 1.3 Hz, 2H), 1.70 (m, 1H), 1.48 – 1.28 (m, 8H), 0.91 (m, 6H). ^{13}C NMR (126 MHz, 300K, $CDCl_3$) δ 160.2, 140.7, 114.5, 91.8, 71.4, 39.4, 30.6, 29.2, 23.9, 23.1, 14.2, 11.2. HRMS (APCI) calcd for $C_{14}H_{19}Cl_2IO$: 399.9858 [M], found: 399.9859; IR ($CHCl_3$) $\tilde{\nu}$=2961(s), 2930(s), 2873(m), 2861(m), 1635(w), 1592(m), 1497(s), 1449(s), 1310(m), 1299(m), 1252(m), 1164(w), 1095(s), 1065(m), 1020(m), 946(w), 931(w), 850(w), 830(w) cm^{-1};

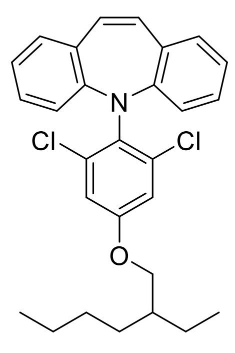

10

General procedure for the synthesis of 10:

1,3-Dichloro-2-iodo-5-(2-ethylhexyloxy)benzene (**9**) (3.15 g, 7.85 mmol, 1.1 equiv.), 5H-dibenz[*b*,*f*]azepine (1.39 g, 7.19 mmol, 1.0 equiv.) and NaO*tBu* (1.27 g, 14.2 mmol, 2.0 equiv.) were added to an oven-dried and argon flushed pressure tube equipped with magnetic stir bar, following by dry toluene (55 mL). The mixture was purged with argon for 15 minutes. Afterwards, $Pd(OAc)_2$ (160 mg, 0.71 mmol, 0.1 equiv.) and P(*t*Bu)$_3$HBF$_4$ (420 mg, 1.44 mmol, 0.2 equiv.) were added at once and the reaction mixture was purged with argon an additional two minutes and the tube was tightly sealed. The reaction mixture was refluxed (117°C) overnight under inert atmosphere. After cooling the reaction mixture to the room temperature, brine was added and the mixture was extracted 3 times with DCM. The organic layers were combined and dried over anhydrous Na_2SO_4. Organic solvents were removed under vacuum and the crude mixture was purified by a column chromatography separation on silica gel (from hexane to hexane/ethyl acetate, 49:1) affording compound **10** (2.71 g, 81%) as a yellow oil. R_f=0.46 (SiO_2, hexane:ethyl aetate, 19:1). ^{1}H NMR (600 MHz, 300 K, CD_2Cl_2) δ 7.04 (m, 2H), 6.93 – 6.89 (m, 2H), 6.85 (m, 2H), 6.81 (m, 4H), 6.17 (mf, 2H), 3.84 (dd, *J* = 5.7, 2.0 Hz, 2H), 1.74 – 1.68 (m, 1H), 1.48 – 1.36 (m, 4H), 1.33 – 1.28 (m, 4H), 0.92 – 0.88 (m, 6H). ^{13}C NMR (151 MHz, 300 K, CD_2Cl_2) δ 159.0, 149.4, 139.9, 137.1, 133.2, 133.1, 132.1, 129.4, 124.3, 123.7, 116.4, 72.0, 39.8, 30.9, 29.6, 24.3, 23.6, 14.4, 11.4. HRMS (APCI) calcd for $C_{28}H_{29}Cl_2NO$: 466.1704 [M]+H, found: 466.1700; IR ($CHCl_3$) $\tilde{\nu}$=3006(w), 2961(s), 2930(s), 2874(m), 2862(m), 1592(s), 1548(m), 1484(s), 1457(s), 1435(m), 1389(w), 1306(m), 1286(m), 1247(m), 1119(w), 1066(m), 1034(m), 940(w), 902(w), 859(w) cm^{-1};

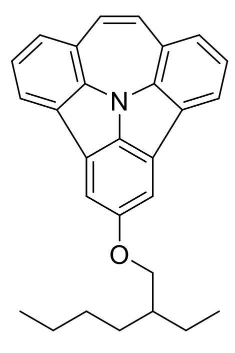

11

General procedure for the synthesis of 11:

Compound **10** (1.46 g, 3.13 mmol, 1.0 equiv.), DBU (2.8 mL) and dry DMAc (7 mL) were added to a reaction vessel equipped with magnetic stir bar. The mixture was purged with argon for 10 minutes. Subsequently, $Pd(PCy_3)_2Cl_2$ (237 mg, 0.32 mmol, 0.1 equiv.) was added to the vessel and the reaction mixture was purged with argon an additional two minutes. The tube was placed in microwave reactor for 50 minutes (150W, 180°C). After cooling it, most of the solvent was removed under vacuum by co-evaporation with hexane. The residue was diluted with DCM and extracted with water (notably, extraction was repeated until removal of DMAc from the mixture). The organic layer was dried over anhydrous Na_2SO_4. Solvents were evaporated and the crude product was purified by a column chromatography separation on silica gel (from hexane to hexane/ethyl acetate, 49:1) affording the desired product **11** (1.03 g, 84%) as an orange-red oil. R_f= 0.50 (SiO_2, hexane:ethyl acetate, 19:1). ^{1}H NMR (400 MHz, 300 K, $CDCl_3$) δ 7.49 (dd, *J* = 7.9, 0.8 Hz, 2H), 7.45 (s, 2H), 6.82 (t, *J* = 7.7 Hz, 2H), 6.67 (dd, *J* = 7.4, 0.8 Hz, 2H), 5.91 (s, 2H), 4.03 – 3.96 (m, 2H), 1.80 (m, 1H), 1.64 – 1.54 (m, 2H), 1.48 (m, 2H), 1.41 – 1.32 (m, 4H), 0.98 (t, *J* = 7.5 Hz, 3H), 0.95 –

0.91 (m, 3H). ^{13}C NMR (151 MHz, 300 K, $CDCl_3$) δ 158.5, 142.8, 139.5, 129.8, 129.2, 126.4, 125.9, 124.4, 123.3, 120.4, 109.2, 73.5, 39.9, 30.8, 29.3, 24.1, 23.3, 14.3, 11.3. HRMS (APCI) calcd for $C_{28}H_{27}NO$: 394.2171 [M]+H, found: 394.2177; IR ($CHCl_3$) $\tilde{\nu}$=3060(w), 3006(w), 2961(s), 2930(s), 2873(m), 2861(m), 1910(w), 1795(w), 1632(m), 1601(m), 1487(s), 1451(m), 1367(m), 1335(m), 1297(s), 1256(s), 1164(s), 1149(m), 1082(m), 1036(m), 992(m), 898(w), 862(w), 838(w), 811(w) cm^{-1};

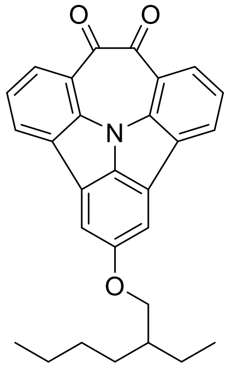

12

General procedure for the synthesis of 12:

A 100 mL two-necked round-bottomed flask was charged with **11** (1.03 g, 2.62 mmol, 1.0 equiv.) and dry chlorobenzene (25 mL). The solution was purged with argon for 15 minutes. Afterwards, benzeneseleninic anhydride (BSA) (1.04 g, 2.88 mmol, 1.1 eqiuv.) was added to the flask. The reaction mixture was refluxed (140°C) overnight with reflux condenser under argon atmosphere. After cooling it to the room temperature, the solvent was removed under vacuum. The residue was diluted with DCM and extracted with water and brine. Aqueous phases were extracted once with DCM. Combine organic layers were dried over anhydrous Na_2SO_4. Solvents were evaporated and the crude product was passed through silica pad (DCM) to remove residual starting material. The subsequent recrystallization from DCM/MeOH affording pure compound **12** (880 mg, 80%) as an orange solid. R_f=0.29 (SiO_2, DCM:triethylamine, 99:1). ^{1}H NMR (400 MHz, 300K, $CDCl_3$) δ 8.28 (d, *J* = 8.0 Hz, 2H), 8.22 (d, *J* = 7.5 Hz, 2H), 7.58 (s, 2H), 7.48 (t, *J* = 7.8 Hz, 2H), 4.05 (d, *J* = 5.8 Hz, 2H), 1.86 (m, 1H), 1.71 – 1.56 (m, 3H), 1.52 – 1.35 (m, 5H), 1.04 (t, *J* = 7.4 Hz, 3H), 1.00 – 0.93 (m, 3H). ^{13}C NMR (151 MHz, 300K, $CDCl_3$) δ 183.0, 159.6, 138.8, 135.7, 131.0, 129.7, 129.1, 124.0, 119.5, 118.5, 110.1, 73.2, 39.9, 30.8, 29.4, 24.2, 23.3, 14.3, 11.4. HRMS (EI) calcd for $C_{28}H_{25}NO_3$: 423.1834 [M]+, found: 423.1842; IR ($CHCl_3$) $\tilde{\nu}$=3032(w), 3003(w), 2961(s), 2930(s), 2873(m), 2861(m), 1733(w), 1662(s), 1630(m), 1612(m), 1507(s), 1496(s), 1453(m), 1379(m), 1296(m), 1241(m), 1119(s), 1088(s), 1052(m), 994(m), 885(w) cm^{-1};

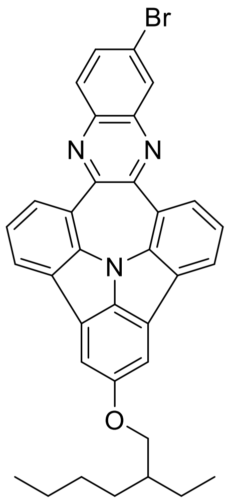

13

General procedure for the synthesis of 13:

A Schlenk tube was charged with compound **12** (500 mg, 1.18 mmol, 1.0 equiv.) and 4-bromo-1,2-diaminobenzene (221 mg, 1.18 mmol, 1.0 equiv.), following by glacial acetic acid (40 mL) and anhydrous ethyl alcohol 99.8% (40 mL). The mixture was argon purged for 20 minutes and reaction was stirred overnight at 120°C under inert atmosphere. After cooling the mixture, precipitate was filtered off , washed with water (3x30mL) and methanol (3x30mL) and dried thoroughly under vacuum affording pure compound **13** (560 mg, 83%) as an orange solid. R_f=0.65 (SiO_2, hexane:DCM, 1:1). ^{1}H NMR (400 MHz, 300K, $CDCl_3$) δ 8.26 (d, *J* = 7.8 Hz, 2H), 8.02 (d, *J* = 2.1 Hz, 1H), 7.78 (d, *J* = 7.7,f 2H), 7.67 (d, *J* = 8.8 Hz, 1H), 7.61 (dd, *J* = 8.8, 2.1 Hz, 1H), 7.49 (s, 2H), 7.16 (t, *J* = 7.8 Hz, 2H), 4.08 – 3.99 (m, 2H), 1.84 (m, 1H), 1.69 – 1.54 (m, 3H), 1.52 – 1.36 (m, 5H), 1.02 (t, *J* = 7.5 Hz, 3H), 0.99 – 0.92 (m, 3H). ^{13}C NMR (151 MHz, 300K, $CDCl_3$) δ 158.9, 150.7, 150.2, 141.0, 140.7, 140.4, 140.4, 139.3, 133.4, 131.2, 130.5, 130.5, 130.1, 127.4, 127.3, 125.7, 125.6, 123.9, 123.7, 123.6, 123.4, 123.4, 120.6, 120.6, 108.9, 73.3, 39.9, 30.8, 29.4, 24.2, 23.3, 14.3, 11.4. HRMS (EI) calcd for $C_{34}H_{28}BrN_3O$: 573.1416 [M]+, found: 573.1404; IR ($CHCl_3$) $\tilde{\nu}$=2962(s), 2930(s), 2874(m), 2861(m), 1676(w), 1581(s), 1548(s), 1462(m), 1424(s), 1382(m), 1282(s), 1242(m), 1181(w), 1119(s), 1036(m), 918(w), 859(w), 837(w) cm^{-1};

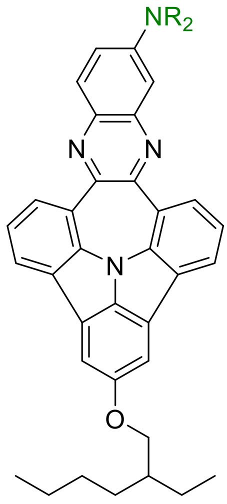

1 - 6

General procedure for the synthesis of 1-6:

Compound **13** (100 mg, 0.174 mmol, 1.00 equiv.), appropriate amine (0.183 mmol, 1.05 equiv.) and NaO*tBu* (17.6 mg, 0.183 mmol, 1.05 equiv.) were added to an oven-dried and argon flushed pressure tube equipped with magnetic stir bar, following by dry toluene (10 mL). The mixture was purged with argon for 15 minutes. Afterwards, $Pd_2(dba)_3$ (11.9 mg, 0.013 mmol, 0.075 equiv.) and XPhos (12.4 mg, 0.026 mmol, 0.15 equiv.) were added at once and the reaction mixture was purged with argon an additional two minutes and the tube was tightly sealed. The reaction mixture was refluxed (117°C) overnight under inert atmosphere. After cooling the reaction mixture to the room temperature, the solvent was removed under vacuum and the residue was diluted with DCM. The brine was added and the mixture was extracted 3 times with DCM. The organic layers were combined and dried over anhydrous Na_2SO_4. Organic solvents were removed under vacuum and the crude mixture was purified by a column chromatography separation on silica gel (hexane/DCM, 2:1). The subsequent recrystallization from DCM/MeOH affording pure compounds **1-6**.

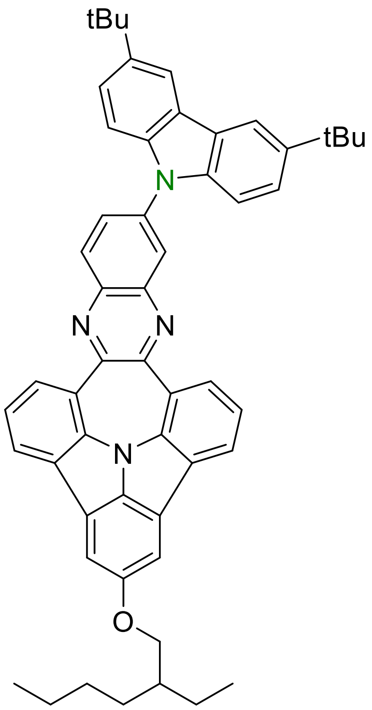

1

1, Orange solid, yield: 89%.

R_f=0.70 (SiO_2, hexane:DCM, 1:1).

^{1}H NMR (600 MHz, 300K, CD_2Cl_2) δ 8.30 (d, *J* = 7.9 Hz, 1H), 8.28 (d, *J* = 7.9 Hz, 1H), 8.19 (d, *J* = 1.9 Hz, 2H), 8.10 (d, *J* = 2.3 Hz, 1H), 8.03 (d, *J* = 8.7 Hz, 1H), 7.86 (dd, *J* = 8.7, 2.3 Hz, 1H), 7.76 (dd, *J* = 7.6, 0.8 Hz, 1H), 7.73 (dd, *J* = 7.6, 0.9 Hz, 1H), 7.58 (d, *J* = 8.6 Hz, 2H), 7.51 – 7.47 (m, 4H), 7.10 (td, *J* = 7.7, 6.2 Hz, 2H), 4.01 (m, 2H), 1.80 (hept, *J* = 6.2 Hz, 1H), 1.62 – 1.52 (m, 4H), 1.47 (s, 18H), 1.42 – 1.35 (m, 4H), 0.99 (t, *J* = 7.5 Hz, 3H), 0.96 – 0.92 (m, 3H).

^{13}C NMR (151 MHz, 300K, CD_2Cl_2) δ 159.5, 151.2, 150.4, 144.3, 141.9, 141.5, 141.3, 141.2, 140.3, 139.8, 139.3, 131.1, 131.0, 130.9, 129.4, 127.9, 127.7, 126.2, 126.0, 125.1, 124.5, 124.4, 124.4, 123.9, 123.9, 121.3, 117.0, 110.0, 109.3, 109.3, 73.7, 40.4, 35.3, 32.3, 31.2, 29.8, 24.6, 23.7, 14.5, 11.6;

HRMS (EI) calcd for $C_{54}H_{52}N_4O$: 772.4141 [M]+, found: 772.4121; IR ($CHCl_3$) $\tilde{\nu}$=2956(m), 2928(w), 2868(w), 1640(w), 1607(m), 1505(m), 1486(s), 1470(m), 1453(m), 1365(m), 1296(m), 1255(m), 1212(s), 1162(w), 1094(s), 1017(w), 878(w), 839(w), 810(m), 754(m), 734(s), 662(m), 598(m) cm^{-1};

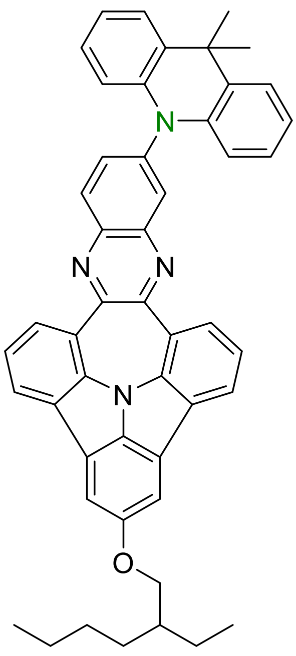

2

2, Orange solid, yield: 74%.

R_f=0.57 (SiO_2, hexane:DCM, 1:1).

^{1}H NMR (500 MHz, 300K, $CDCl_3$) δ 8.43 (d, *J* = 8.0, 1H), 8.35 (d, *J* = 8.0, 1H), 8.20 (d, *J* = 8.7 Hz, 1H), 8.05 (d, *J* = 2.2 Hz, 1H), 7.88 (d, *J* = 7.7, 1H), 7.84 (d, *J* = 7.6, 1H), 7.60 (dd, *J* = 8.7, 2.2 Hz, 1H), 7.55 – 7.49 (m, 4H), 7.28 (d, *J* = 7.8 Hz, 1H), 7.23 – 7.17 (m, 1H), 7.06 – 6.96 (m, 4H), 6.50 (dd, *J* = 8.1, 1.4 Hz, 2H), 4.08 – 4.01 (m, 2H), 1.85 (m, 1H), 1.75 (s, 6H), 1.65 – 1.57 (m, 2H), 1.53 – 1.34 (m, 6H), 1.01 (t, *J* = 7.5 Hz, 3H), 0.99 – 0.93 (m, 3H).

^{13}C NMR (126 MHz, 300K, $CDCl_3$) δ 159.1, 150.9, 150.7, 143.0, 142.2, 141.6, 141.3, 140.7, 140.4, 133.5, 131.9, 131.4, 130.9, 130.8, 130.8, 127.6, 127.6, 126.6, 125.9, 125.9, 125.5, 124.2, 124.0, 123.8, 123.7, 121.3, 121.2, 121.2, 114.6, 109.0, 73.4, 39.9, 36.3, 31.2, 30.8, 29.3, 24.2, 23.3, 14.3, 11.4;

HRMS (EI) calcd for $C_{49}H_{42}N_4O$: 702.3359 [M]+, found: 702.3345; IR (KBr) $\tilde{\nu}$=3056(w), 2956(m), 2923(m), 2856(w), 1693(w), 1592(m), 1503(s), 1476(m), 1449(s), 1325(m), 1248(s), 1214(m), 1162(w), 1094 (s), 1062(w), 1017(w), 930(w), 791(w), 743(m), 669(w) cm^{-1};

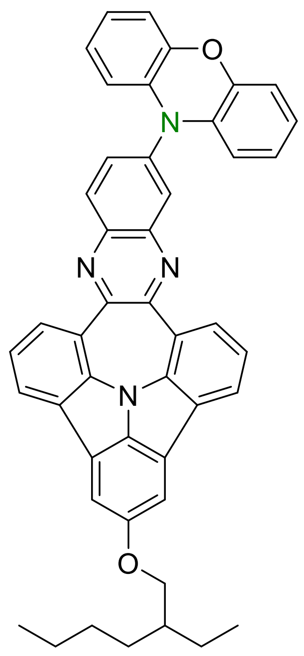

3

3, Red solid, yield: 89%.

R_f=0.62 (SiO_2, hexane:DCM, 1:1).

^{1}H NMR (500 MHz, 300K, $CDCl_3$) δ 8.40 – 8.35 (d, *J* = 7.8 Hz, 1H), 8.33 (d, *J* = 8.0 Hz, 1H), 8.15 (d, *J* = 8.6 Hz, 1H), 8.02 (d, *J* = 2.2 Hz, 1H), 7.89 – 7.79 (m, 2H), 7.60 (dd, *J* = 8.7, 2.2 Hz, 1H), 7.51 (m, 2H), 7.24 (t, *J* = 8.0 Hz, 1H), 7.19 (t, *J* = 7.8 Hz, 1H), 6.78 – 6.61 (m, 6H), 6.15 (dd, *J* = 8.0, 1.5 Hz, 2H), 4.08 – 4.01 (m, 2H), 1.83 (hept, *J* = 6.1 Hz, 1H), 1.67 – 1.56 (m, 2H), 1.54 – 1.32 (m, 6H), 1.02 (t, *J* = 7.4 Hz, 3H), 0.99 – 0.93 (m, 3H).

^{13}C NMR (126 MHz, 300K, $CDCl_3$) δ 159.1, 151.0, 150.7, 144.2, 142.1, 141.5, 141.2, 140.6, 140.5, 133.9, 132.7, 132.3, 131.4, 130.9, 130.8, 127.6, 127.5, 126.0, 125.9, 124.0, 123.9, 123.7, 123.5, 122.1, 121.1, 121.1, 115.9, 113.7, 109.0, 73.3, 39.9, 30.8, 29.3, 24.2, 23.3, 14.3, 11.4;

HRMS (EI) calcd for $C_{46}H_{36}N_4O_2$: 676.2838 [M]+, found: 676.2831; IR ($CHCl_3$) $\tilde{\nu}$=3060(w), 2958(m), 2926(m), 2871(w), 1603(w), 1503(m), 1487(s), 1462(m), 1333(m), 1273(m), 1251(m), 1215(m), 1162(w), 1094(m), 1018(w), 791(w), 737(m), 662(m), 596(w) cm^{-1};

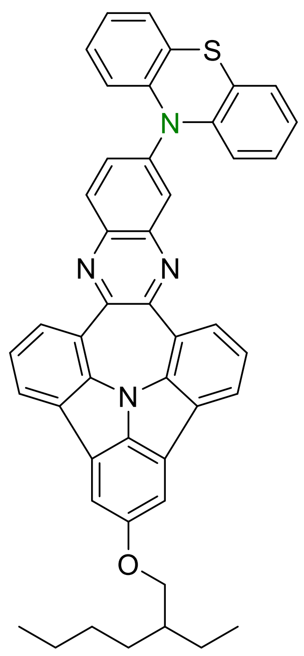

4

4, Orange solid, yield: 74%.

R_f=0.63 (SiO_2, hexane:DCM, 1:1).

^{1}H NMR (500 MHz, 300K, $CDCl_3$) δ 8.34 (d, *J* = 7.9 Hz, 1H), 8.30 (d, *J* = 7.9 Hz, 1H), 7.95 (d, *J* = 8.9 Hz, 1H), 7.81 (dd, *J* = 7.6, 4.9 Hz, 2H), 7.75 (d, *J* = 2.5 Hz, 1H), 7.54 (dd, *J* = 8.9, 2.5 Hz, 1H), 7.51 (d, *J* = 1.7 Hz, 2H), 7.29 (dd, *J* = 7.7, 1.5 Hz, 2H), 7.21 (m, 1H), 7.19 – 7.12 (m, 3H), 7.06 (td, *J* = 7.5, 1.3 Hz, 2H), 6.97 (dd, *J* = 8.1, 1.3 Hz, 2H), 4.08 – 4.01 (m, 2H), 1.83 (hept, *J* = 6.2 Hz, 1H), 1.66 – 1.56 (m, 2H), 1.52 – 1.34 (m, 6H), 1.01 (t, *J* = 7.5 Hz, 3H), 0.98 – 0.93 (m, 3H). ^{13}C NMR (126 MHz, 300K, $CDCl_3$) δ 159.0, 150.6, 149.2, 144.9, 142.8, 142.0, 141.7, 141.3, 141.2, 138.8, 130.9, 130.8, 128.1, 127.6, 127.5, 127.4, 127.3, 125.7, 125.4, 124.7, 124.4, 124.2, 123.7, 123.6, 121.7, 121.4, 121.3, 121.3, 109.0, 108.9, 73.3, 39.9, 30.8, 29.3, 24.2, 23.3, 14.3, 11.4;
HRMS (EI) calcd for $C_{46}H_{36}N_4OS$: 692.2610 [M]+, found: 692.2603; IR (KBr) $\tilde{\nu}$=3434(m), 3058(w), 2955(m), 2923(m), 2855(m), 1607(w), 1587(m), 1503(m), 1460(s), 1442(m), 1305(m), 1245(m), 1211(m), 1159 (w), 1092(s), 1015(w), 928(w), 790(w), 736(s), 655(w) cm^{-1};

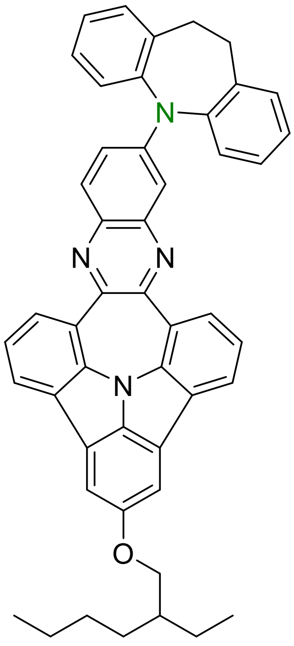

5

5, Yellow solid, yield: 50%.

R_f=0.48 (SiO_2, hexane:DCM, 1:1).

^{1}H NMR (500 MHz, 300K, $CDCl_3$) δ 8.36 – 8.26 (m, 2H), 7.82 (d, *J* = 7.7 Hz, 2H), 7.71 (d, *J* = 9.2, 1H), 7.54 – 7.49 (m, 4H), 7.39 – 7.29 (m, 6H), 7.23 (dd, *J* = 5.8, 2.5 Hz, 1H), 7.17 (t, *J* = 7.7 Hz, 1H), 7.13 (dd, *J* = 9.3, 2.7 Hz, 1H), 6.97 (d, *J* = 2.7 Hz, 1H), 4.04 (dd, *J* = 5.6, 2.8 Hz, 2H), 3.07 (s, 4H), 1.82 (p, *J* = 6.2 Hz, 1H), 1.61 (m, 2H), 1.54 – 1.32 (m, 6H), 1.00 (t, *J* = 7.5 Hz, 3H), 0.97 – 0.91 (m, 3H).

^{13}C NMR (126 MHz, 300K, $CDCl_3$) δ 159.0, 150.6, 150.2, 145.9, 143.0, 142.5, 142.1, 141.7, 141.3, 138.2, 135.8, 131.4, 130.7, 130.6, 129.7, 128.0, 127.7, 127.4, 126.8, 125.3, 125.1, 124.8, 124.6, 123.7, 123.5, 121.8, 121.6, 120.0, 108.8, 108.8, 106.8, 73.3, 39.9, 30.9, 30.8, 29.3, 24.2, 23.3, 14.3, 11.4;

HRMS (EI) calcd for $C_{48}H_{40}N_4O$: 688.3202 [M]+, found: 688.3183; IR (KBr) $\tilde{\nu}$=3434(m), 3058(w), 2956(m), 2923(m), 2856(m), 1611(m), 1597(s), 1479(s), 1449(m), 1333(m), 1312(m) 1245(s), 1222(m), 1162(w), 1095(s), 1018(w), 823(w), 793(w), 740(m) cm^{-1};

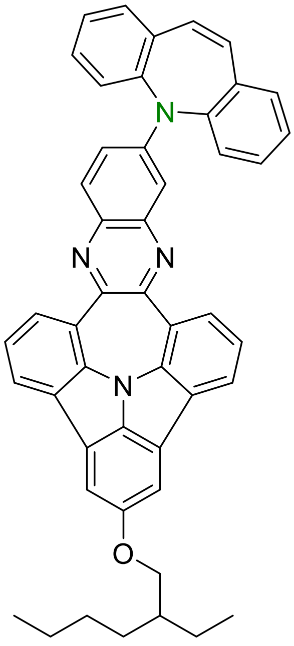

6

6, Yellow solid, yield: 58%.

R_f=0.55 (SiO_2, hexane:DCM, 1:1).

^{1}H NMR (500 MHz, 300K, $CDCl_3$) δ 8.30 (d, *J* = 8.1, 1H), 8.26 (d, *J* = 7.9, 1H), 7.80 (d, *J* = 7.6, 2H), 7.64 – 7.57 (m, 5H), 7.55 – 7.48 (m, 4H), 7.45 (m, 2H), 7.22 (t, *J* = 7.9, 1H), 7.16 (t, *J* = 7.9, 1H), 6.90 (s, 2H), 6.82 (dd, *J* = 9.3, 2.7 Hz, 1H), 6.67 (d, *J* = 2.7 Hz, 1H), 4.07 – 3.99 (m, 2H), 1.82 (hept, *J* = 6.2 Hz, 1H), 1.61 (m, 2H), 1.53 – 1.32 (m, 6H), 1.00 (t, *J* = 7.5 Hz, 3H), 0.97 – 0.91 (m, 3H).

^{13}C NMR (126 MHz, 300K, $CDCl_3$) δ 159.0, 150.4, 150.1, 145.8, 142.4, 142.2, 142.1, 141.7, 141.3, 136.2, 136.0, 130.7, 130.6, 130.6, 130.3, 130.0, 129.4, 127.8, 127.4, 126.8, 125.2, 125.1, 124.8, 124.6, 123.7, 123.5, 121.8, 121.6, 119.4, 108.8, 108.7, 106.3, 73.3, 39.9, 30.8, 29.3, 24.2, 23.3, 14.3, 11.4.

HRMS (EI) calcd for $C_{48}H_{38}N_4O$: 686.3046 [M]+, found: 686.3044; IR ($CHCl_3$) $\tilde{\nu}$=3062(w), 2956(m), 2926(m), 1610(s), 1593(m), 1485(s), 1452(m), 1335(m), 1312(m), 1248(s), 1222(m), 1162(w), 1095(s), 1018(w), 824(w), 794(m), 756(m), 739(m), 598(w) cm^{-1};

SI-3 Steady-state characterisation

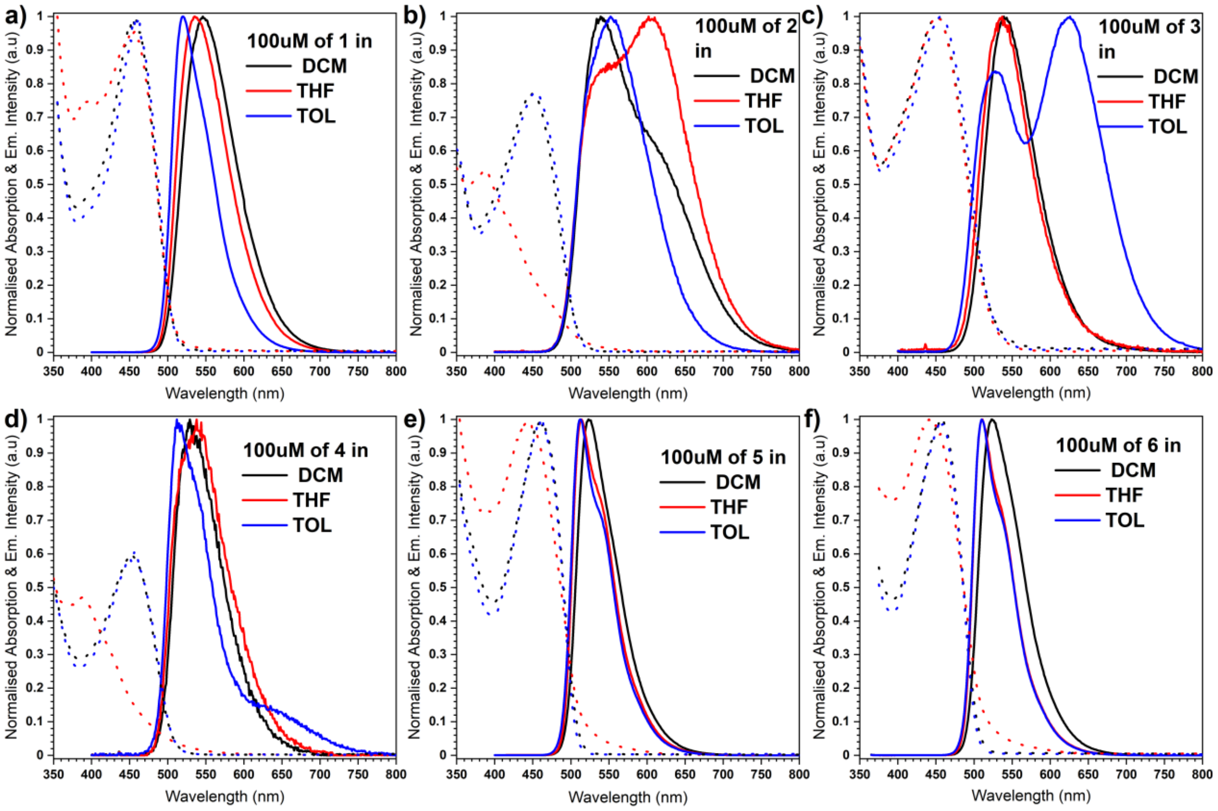

Figure S1. a-f) UV-vis absorption and PL spectra measured for compounds **1-6**, respectively, in diluted solutions (c 10–5 M) of dichloromethane, tetrahydrofuran, and toluene solvents.

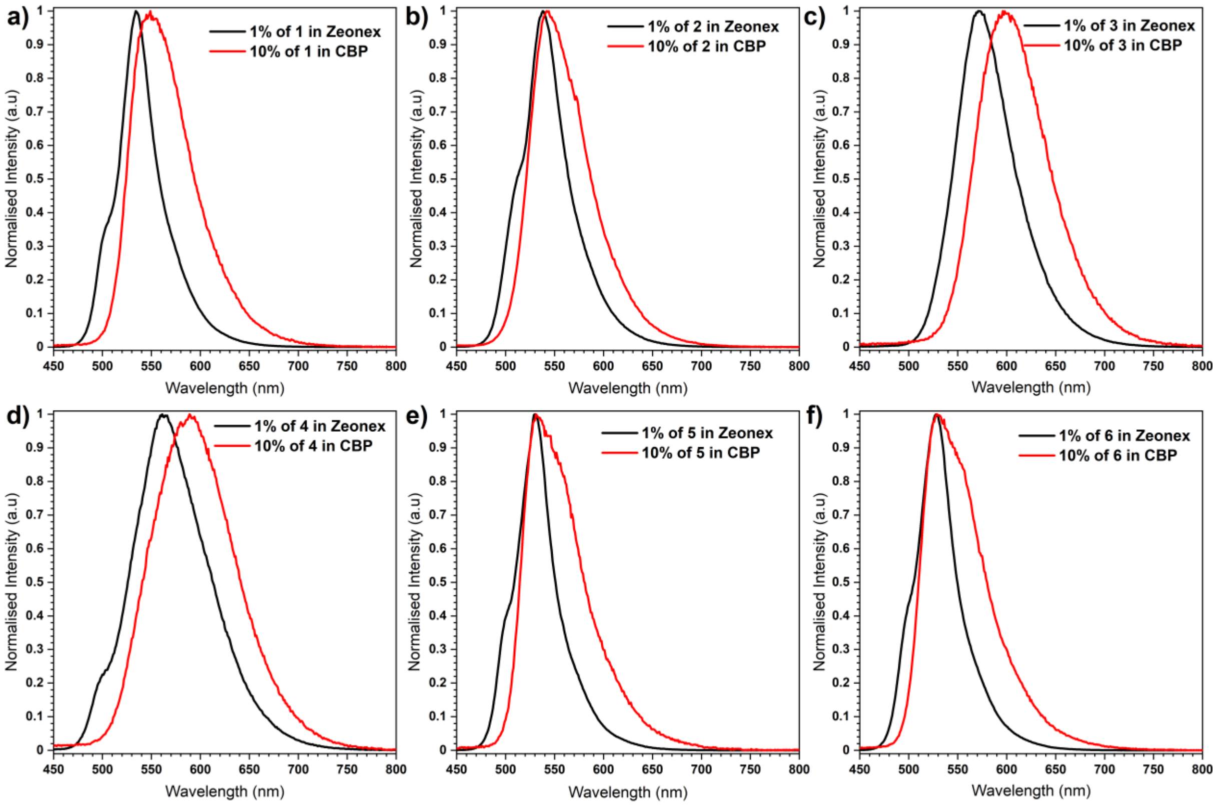

Figure S2. a-f) PL spectra measured for compounds **1-6,** respectively, in solid CBP and Zeonex matrix.

SI-4 Cyclic voltammetry

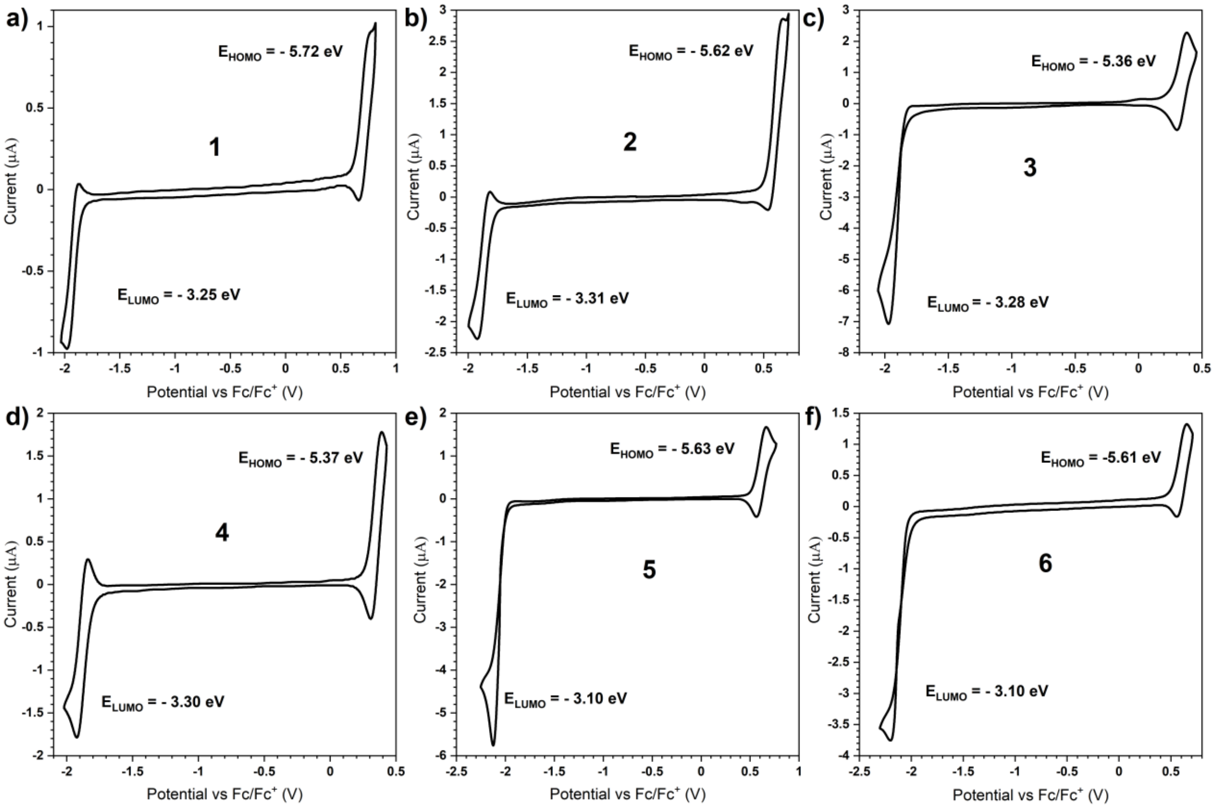

Figure S3. a-f) CV of 1 mM concentration of compounds **1-6**, respectively, in 0.1 M Bu4NBF4 in DCM electrolyte at the scan rate of 50 mV/s.

SI-5 X-Ray crystallography analysis

The X-ray measurement of **2** was performed at 130.0(5) K on a Bruker D8 Venture PhotonII diffractometer equipped with a TRIUMPH monochromator and a MoKα fine focus sealed tube (λ = 0.71073 Å). A total of 2400 frames were collected with Bruker APEX3 program [3]. The frames were integrated with the Bruker SAINT software package [4] using a narrow-frame algorithm. The integration of the data using a triclinic unit cell yielded a total of 37659 reflections to a maximum θ angle of 25.05° (0.84 Å resolution), of which 6342 were independent (average redundancy 5.938, completeness = 99.7%, R_{int} = 8.87%, R_{sig} = 6.22%) and 4232 (66.73%) were greater than $2\sigma(F^2)$. The final cell constants of a = 8.5144(7) Å, b = 10.6983(9) Å, c = 20.5982(17) Å, α = 103.982(3)°, β = 96.845(3)°, γ = 94.261(3)°, V = 1797.3(3) Å^3, are based upon the refinement of the XYZ-centroids of 487 reflections above 20 $\sigma(I)$ with 4.281° < 2θ < 44.67°. Data were corrected for absorption effects using the Multi-Scan method [5]. The ratio of minimum to maximum apparent transmission was 0.829. The calculated minimum and maximum transmission coefficients (based on crystal size) are 0.976 and 0.996.

The structure was solved and refined using SHELXTL Software Package [6,7] using the space group $P\bar{1}$, with Z = 2 for the formula unit, $C_{49}H_{42}N_4O$. The final anisotropic full-matrix least-squares refinement on F^2 with 620 variables converged at $R1$ = 7.88%, for the observed data and $wR2$ = 22.71% for all data. The goodness-of-fit was 1.077. The largest peak in the final difference electron density synthesis was 0.416 e^-/Å^3 and the largest hole was -0.253 e^-/Å^3 with an RMS deviation of 0.069 e^-/Å^3. On the basis of the final model, the calculated density was 1.299 g/cm^3 and $F(000)$, 744 e^-. The details concerning the crystal data and structural parameters of **2** are collected in **Table S*1***.

The aliphatic chain of the organic molecule is disordered over three alternative positions with refined occupancies yielding 0.629(3), 0.262(3) and 0.109(3) for the main, B and C residues respectively. In addition the 9,9-dimethyl-9,10-dihydroacridine moiety is also disordered and located at two possible sites with refined occupancy ratio equal 0.728(2):0.272(2). The disorder model of **2** is presented in **Figure S*4* a)**.

All main residue non-hydrogen atoms were refined anisotropically. All hydrogen atoms were placed in calculated positions and refined within the riding model. To preserve reasonable geometry of the disordered molecule number of distance, angle and ADP restraints were used. The temperature factors of hydrogen atoms were not refined and were set to be 1.2 or 1.5 times

larger than U_{eq} of the corresponding heavy atom. The atomic scattering factors were taken from the International Tables [8]. Molecular graphics was prepared using program Mercury 2020.2.0 [9]. Thermal ellipsoids parameters are presented at 50% probability level in **Figure S*4* b) and c)**.

Table S1. Data collection and structure refinement parameters for **2**.

**Identification code**	**2**
**Formula**	$C_{49}H_{42}N_4O$
M_x**/ g mol^{-1}**	702.86
T**/ K**	130.5(5)
λ**/ Å**	0.71073
**Crystal size**	0.051 × 0.241 × 0.308 mm
**Space group**	$P\bar{1}$
**Unit cell dimensions**	a = 8.5144(7) Å<br>b = 10.6983(9) Å<br>c = 20.5982(17) Å<br>α = 103.982(3)°<br>β = 96.845(3)°<br>γ = 94.261(3)°
V**/ Å^3, Z**	1797.3(3), 2
D_x**/ g cm^{-3}**	1.299
μ**/ mm^{-1}**	0.078
F**(000)**	744
θ_{min}, θ_{max}	2.76°, 25.05°
**Index ranges**	$-10 \leq h \leq 10$, $-12 \leq k \leq 12$, $-24 \leq l \leq 24$
**Reflections collected/ independent**	37659/ 6342 (R_{int} = 0.0887)
**Completeness**	99.7%
**Absorption correction**	Multi-Scan
T_{max}, T_{min}	0.996, 0.976
**Refinement method**	Full-matrix LSQ on F^2
**Data / restraints / parameters**	6342 / 159 / 620
**GOF on F^2**	1.077
**Final R indices**	4232 data; I>2σ(I)<br>R1 = 0.0788, wR2 = 0.2019<br>all data<br>R1 = 0.1174, wR2 = 0.2271
**Extinction coefficient**	0.008(2)
$\Delta\rho_{max}$, $\Delta\rho_{min}$	0.416 eÅ^{-3}, -0.253 eÅ^{-3}

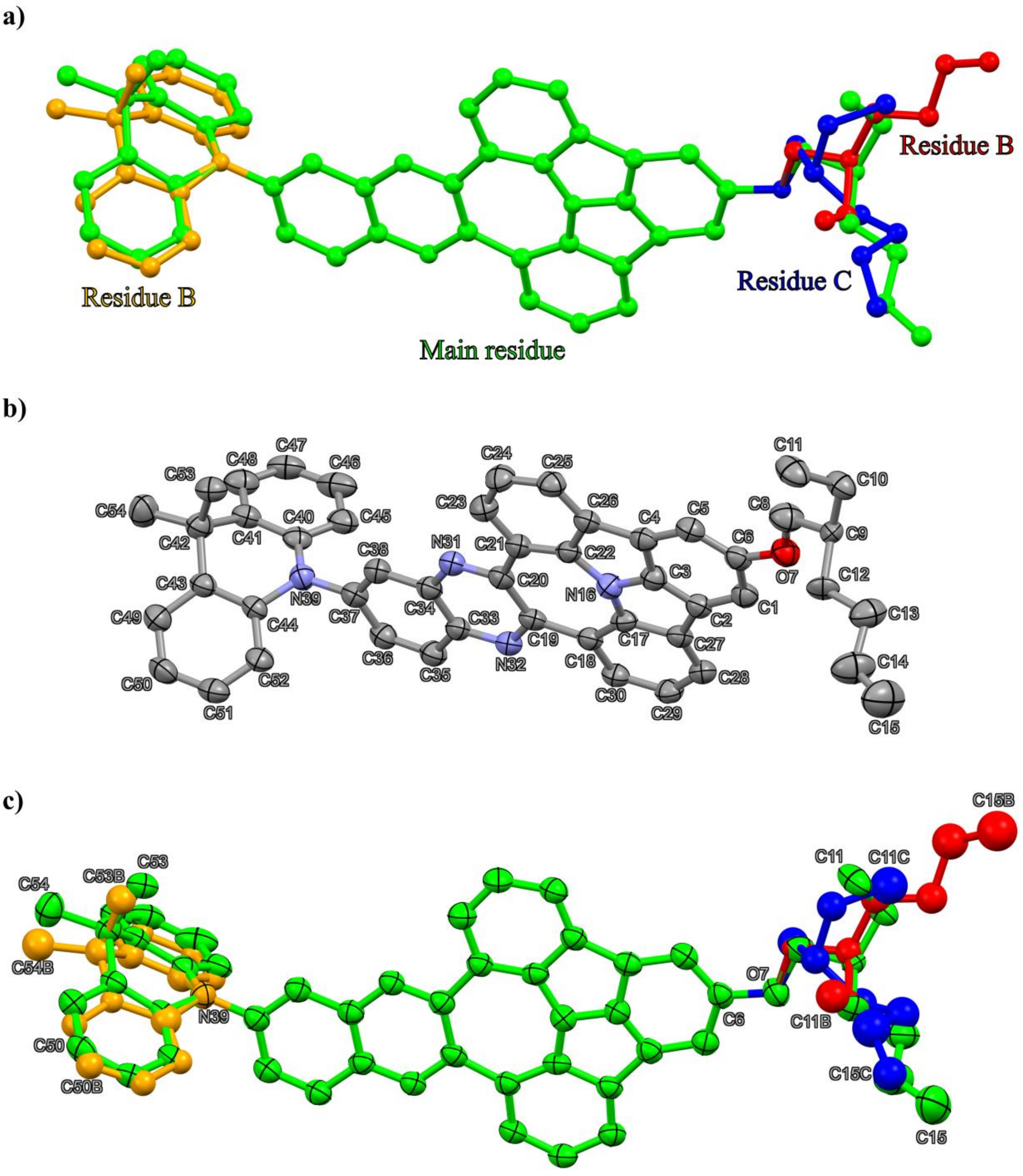

Figure S4. Molecular view of the **2**, green color represent main residue, red and blue colors denote alternative orientation of the aliphatic chain whereas the orange color indicates the other position of 9,9-dimethyl-9,10-dihydroacridine moiety, hydrogen atoms omitted for clarity **a)**. Thermal ellipsoid plot at 50% probability level together with numbering scheme of the main residue **b)**. Thermal ellipsoid plot at 50% probability level for all residues together with numbering scheme of selected atoms, hydrogen atoms omitted for clarity **c)**.

The X-ray measurement of **1** was performed at 130.0(5) K on a Bruker D8 Venture PhotonII diffractometer equipped with a fine focus sealed tube (MoKα, λ = 0.71073 Å) and a TRIUMPH monochromator. A total of 2960 frames were collected with Bruker APEX3 program [3]. The frames were integrated with the Bruker SAINT software package [4] using a narrow-frame algorithm. The integration of the data using a triclinic unit cell yielded a total of 56174 reflections to a maximum θ angle of 25.05° (0.84 Å resolution), of which 8043 were independent (average redundancy 6.984, completeness = 99.7%, R_{int} = 3.40%, R_{sig} = 2.06%) and 7200 (89.52%) were greater than $2\sigma(F^2)$. The final cell constants of a = 8.3168(9) Å, b = 10.9572(12) Å, c = 26.461(3) Å, α = 95.369(4)°, β = 97.431(4)°, γ = 105.835(4)°, V = 2279.3(4) Å^3, are based upon the refinement of the XYZ-centroids of 9698 reflections above 20 $\sigma(I)$ with 5.143° < 2θ < 50.81°. Data were corrected for absorption effects using the Multi-Scan method (SADABS) [5]. The ratio of minimum to maximum apparent transmission was 0.848. The calculated minimum and maximum transmission coefficients (based on crystal size) are 0.863 and 0.969.

The structures were solved and refined using SHELXTL Software Package [6,7] using the space group $P\bar{1}$ with Z = 2 for the formula unit, $C_{55}H_{53}Cl_3N_4O$. The final anisotropic full-matrix least-squares refinement on F^2 with 684 variables converged at $R1$ = 5.26%, for the observed data and $wR2$ = 13.05% for all data. The goodness-of-fit was 1.080. The largest peak in the final difference electron density synthesis was 0.444 e^-/Å^3 and the largest hole was -0.296 e^-/Å^3 with an RMS deviation of 0.046 e^-/Å^3. On the basis of the final model, the calculated density was 1.300 g/cm^3 and $F(000)$, 940 e^-. The details concerning the crystal data and structural parameters of **1** are collected in **Table S*2***.

The structure contains disordered over four alternative positions $CHCl_3$ solvent molecule with refined occupancy ratio yielding 0.656(3):0.177(3):0.088(2):0.079(3). In addition one *tert*-butyl group in the main compound is disordered over two sites with refined occupancy ratio equal 0.693(6):0.307(6). Moreover, aliphatic ether chain is disordered as well, with the moiety distributed over two positions with refined occupancy ratio of 0.817(4):0.183(4).

All ordered non-hydrogen atoms, and major component disordered atoms were refined anisotropically. To preserve reasonable geometry of the disordered fragments a number of distance and angles constraints was used during the structure refinement. All hydrogen atoms were placed in calculated positions and refined within the riding model. The temperature factors of hydrogen atoms were not refined and were set to be 1.2 or 1.5 times larger than U_{eq} of the

corresponding heavy atom. The atomic scattering factors were taken from the International Tables [8]. Molecular graphics was prepared using program Mercury 4.1 [9]. Thermal ellipsoids parameters are presented at 50% probability level in **Figure S5**.

Table S2. Data collection and structure refinement parameters for **1**.

**Identification code**	**1**
**Formula**	$C_{55}H_{53}Cl_3N_4O$ corresponding to:<br>$C_{54}H_{52}N_4O$ + $CHCl_3$
**M_x/ g·mol^{-1}**	892.36
**T/ K**	130.5(5)
**λ/ Å**	0.71073
**Crystal size**	0.127 × 0.149 × 0.614 mm
**Space group**	$P\bar{1}$
**Unit cell dimensions**	a = 8.3168(9) Å<br>b = 10.9572(12) Å<br>c = 26.461(3) Å<br>α = 95.369(4)°<br>β = 97.431(4)°<br>γ = 105.835(4)°
**V/ Å^3, Z**	2279.3(4), 2
**D_x/ g·cm^{-3}**	1.300
**μ/ mm^{-1}**	0.247
**F(000)**	940
**θ_{min}, θ_{max}**	2.57°, 25.05°
**Index ranges**	$-9 \leq h \leq 9$, $-13 \leq k \leq 13$, $-31 \leq l \leq 31$
**Reflections collected/ independent**	56174/ 8043 (R_{int} = 0.0340)
**Completeness**	99.7%
**Absorption correction**	Multi-Scan
**T_{max}, T_{min}**	0.969, 0.863
**Refinement method**	Full-matrix LSQ on F^2
**Data / restraints / parameters**	8043 / 107 / 684
**GOF on F^2**	1.080
**Final R indices**	7200 data; I>2σ(I)<br>R1 = 0.0526, wR2 = 0.1267<br>all data<br>R1 = 0.0582, wR2 = 0.1305
**Extinction coefficient**	0.031(6)
**$\Delta\rho_{max}$, $\Delta\rho_{min}$**	0.444 eÅ^{-3}, -0.296 eÅ^{-3}

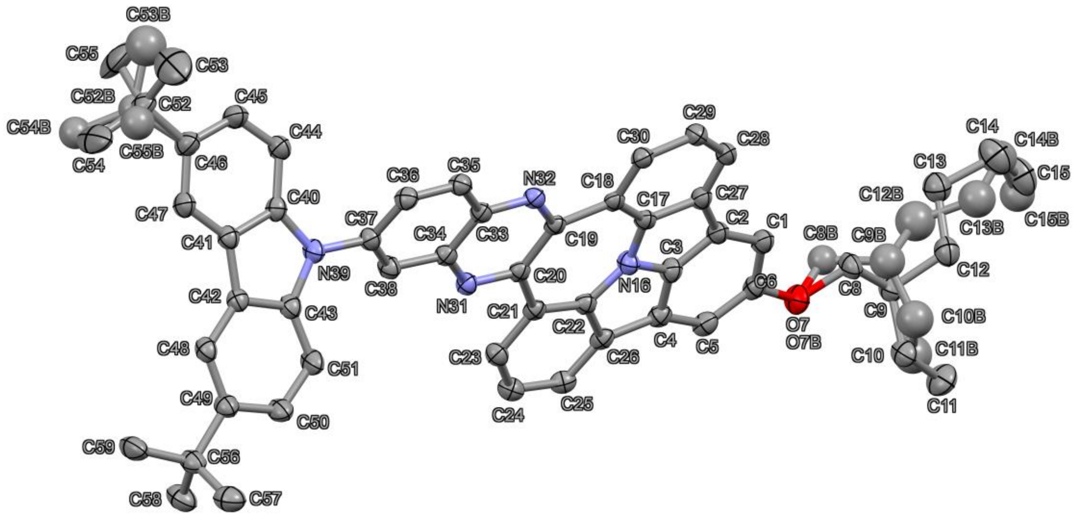

Figure S5. Thermal ellipsoid plot at 50% probability level together with numbering scheme of the atoms in the structure of **1**. Disorder $CHCl_3$ molecule and all hydrogen atoms omitted for clarity.

Molecular geometry of compound 1

As mentioned in Section 2.2 of our paper, we unexpectedly found that, in the molecular crystal phase, compounds **1** and **2** adopt geometries in which the central N-PAH moiety is planar. This is in contrast to the series of N-PAHs reported by our group previously [10], which N-PAH moieties are markedly concave.

In order to visually demonstrate the planarization of the N-PAH moiety of compound **1**, **Figure S*6*** below compares its geometry to that of compound **10l**, which is representative of the series of N-PAHs already reported by our group [10]. The geometries of both compounds were determined with the use of single-crystal X-ray diffraction, and, in **Figure S*6***, they have been superimposed on one another in such a way as to minimize the root mean square distance between atoms in the N-PAH moieties. It can be seen that compound **10l** adopts a noticeably concave geometry. On the other hand, in compound **1**, the N-PAH moiety is planar.

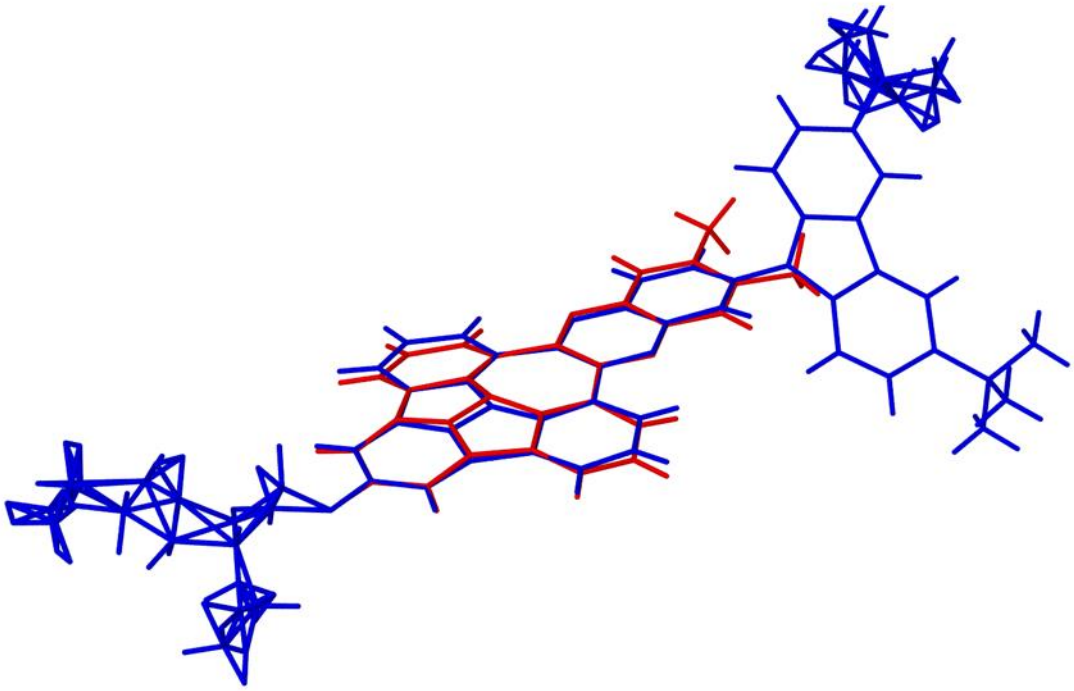

Figure S6. Visual comparison of the geometries of compound **10l** (in red) [10] and compound 1 (in blue). The alkoxy group and one of the two tert-butyl groups of compounds **1** exhibit crystallographic disorder.

SI-6 TGA/DSC and DSC (heating/cooling) measurements

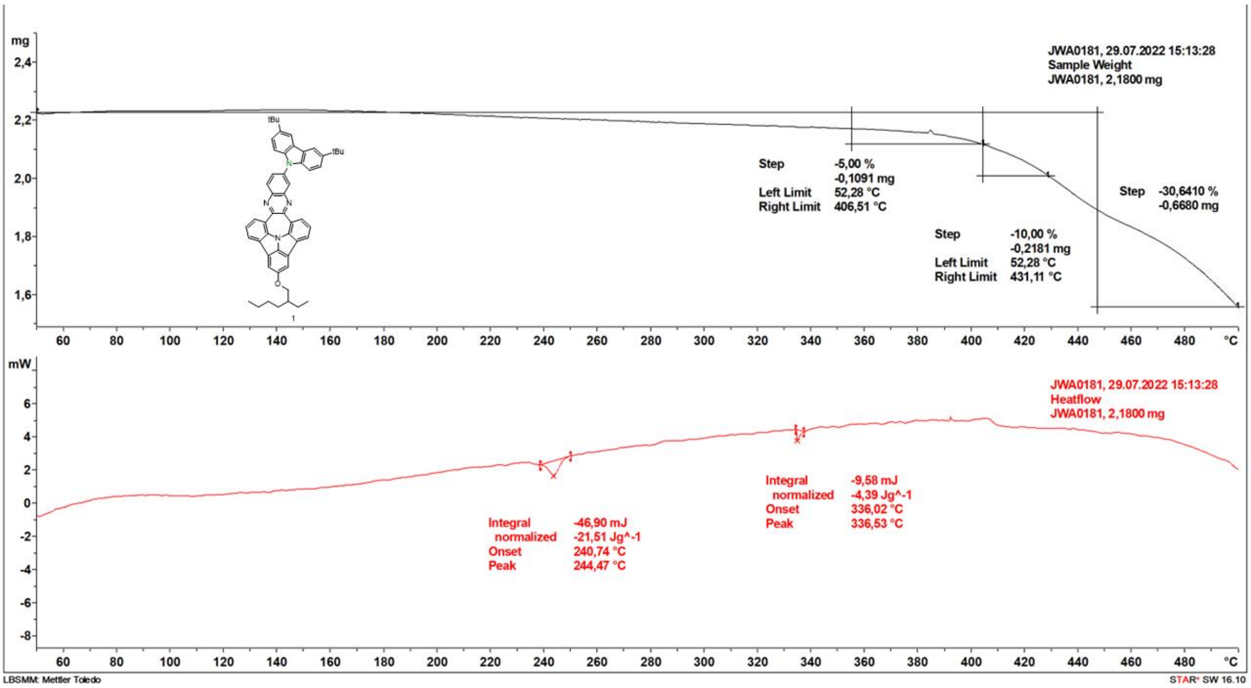

Figure S7. TGA/DSC curve of **1**.

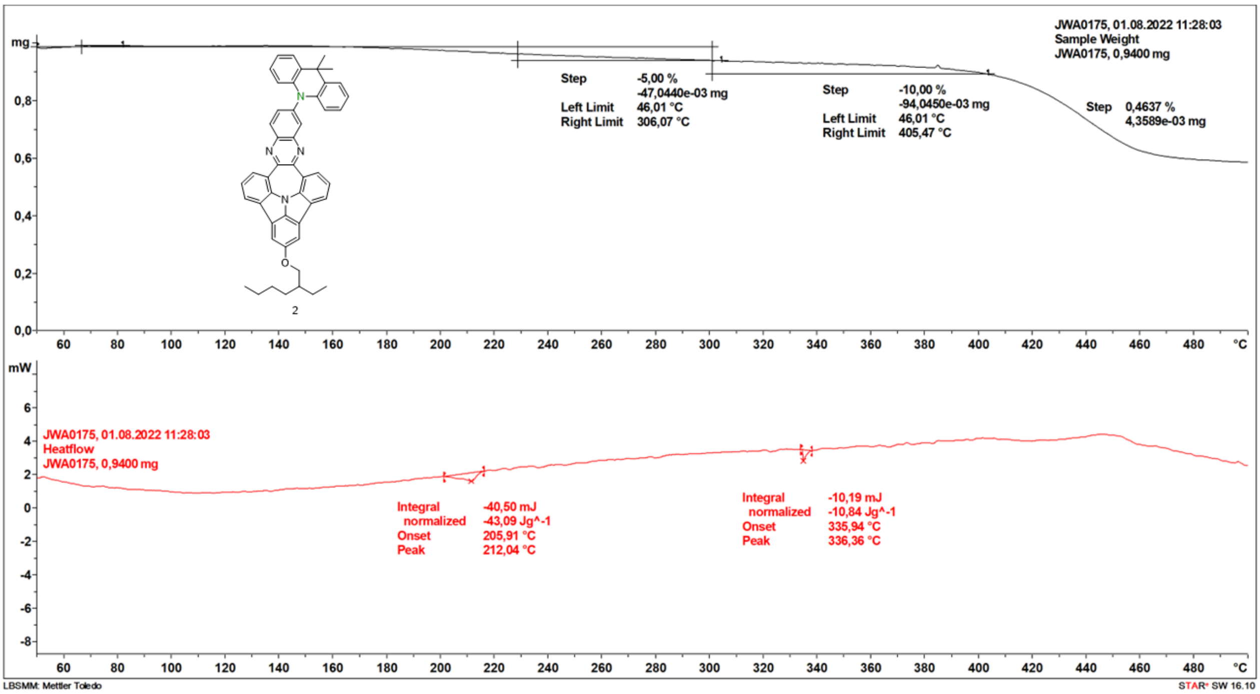

Figure S8. TGA/DSC curve of **2**.

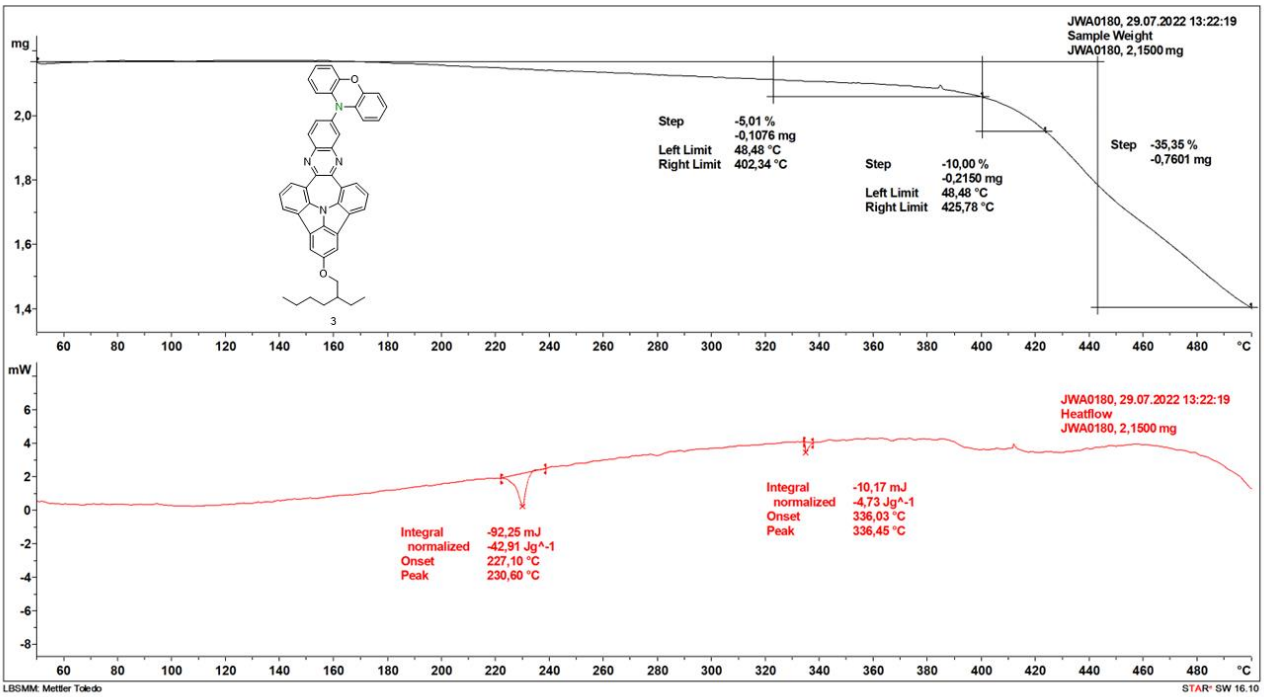

Figure S9. TGA/DSC curve of **3**.

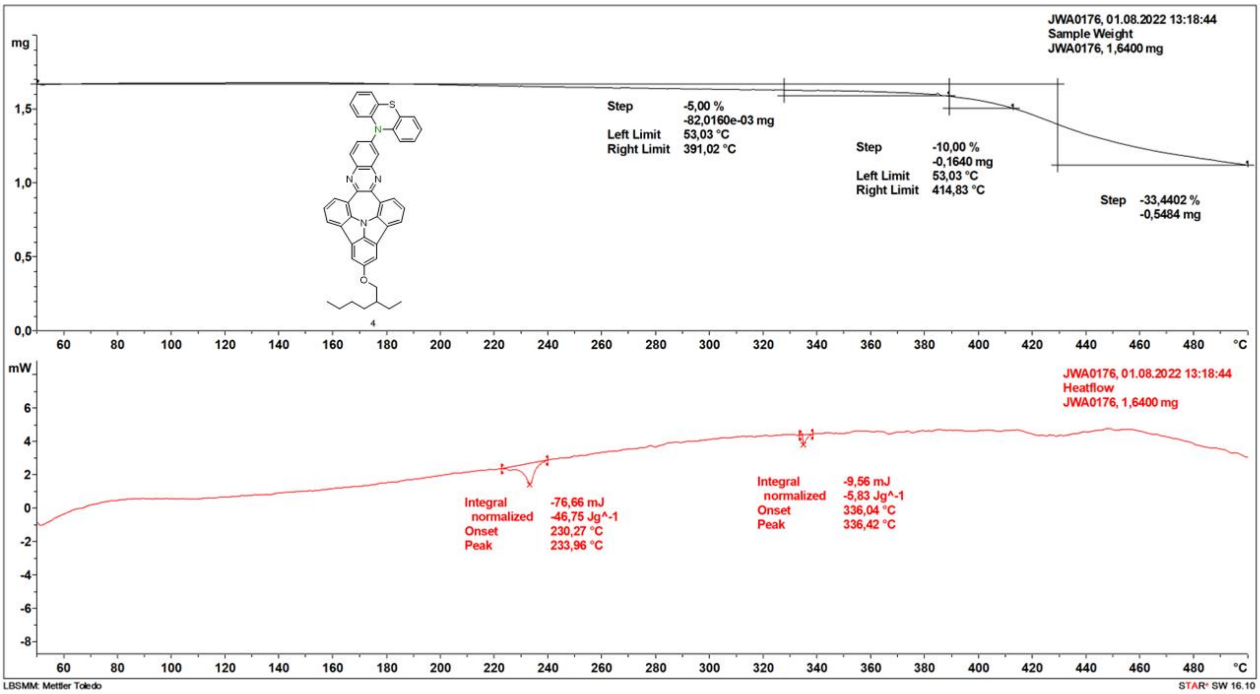

Figure S10. TGA/DSC curve of **4**.

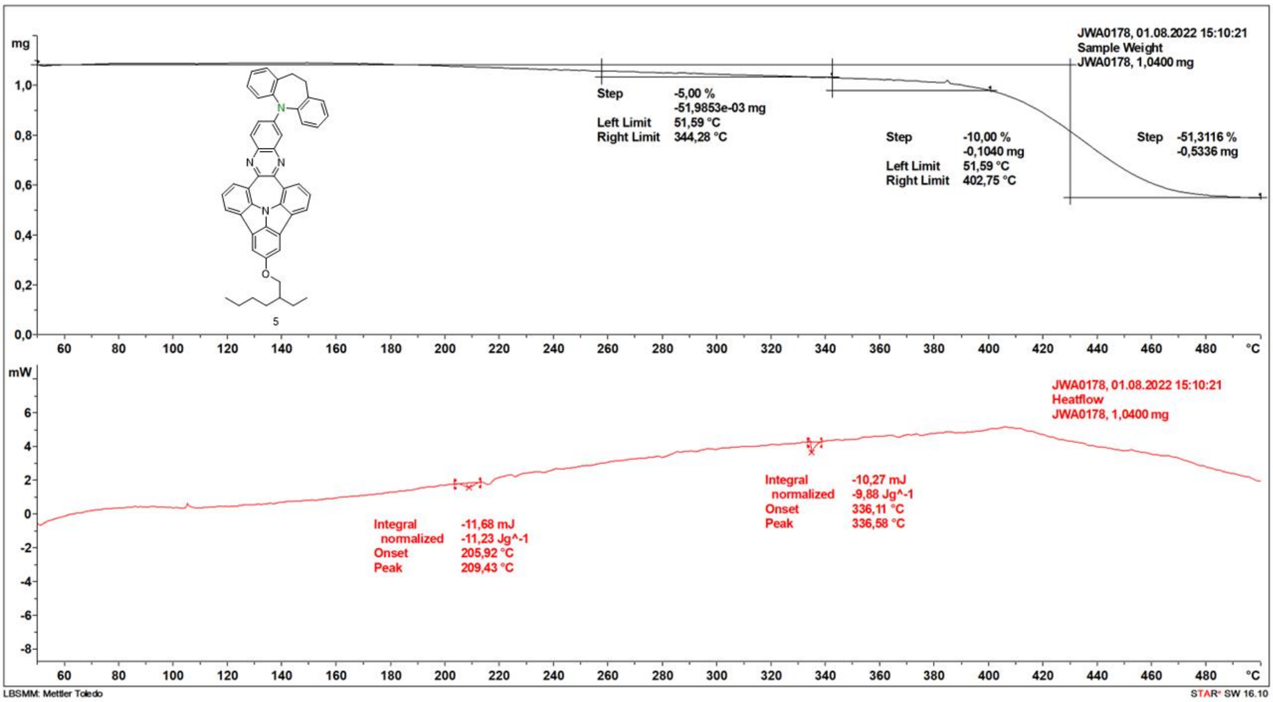

Figure S11. TGA/DSC curve of **5**.

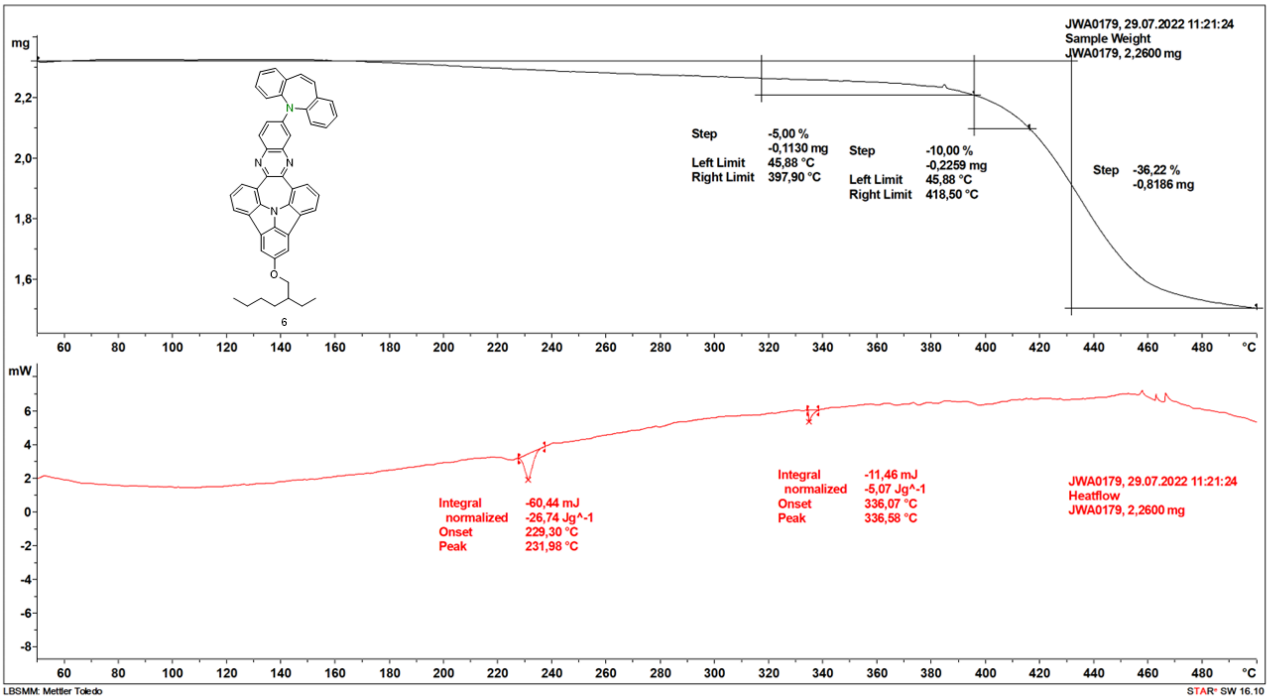

Figure S12. TGA/DSC curve of **6**.

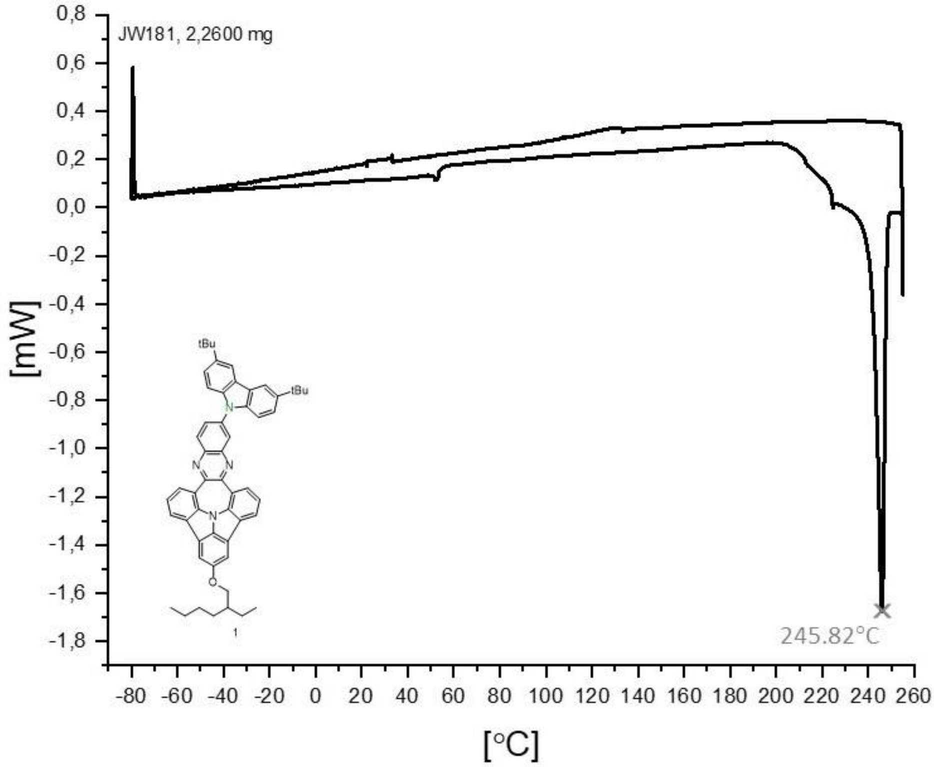

Figure S13. DSC heating/cooling (5ºC/ min) experiment for **1**.

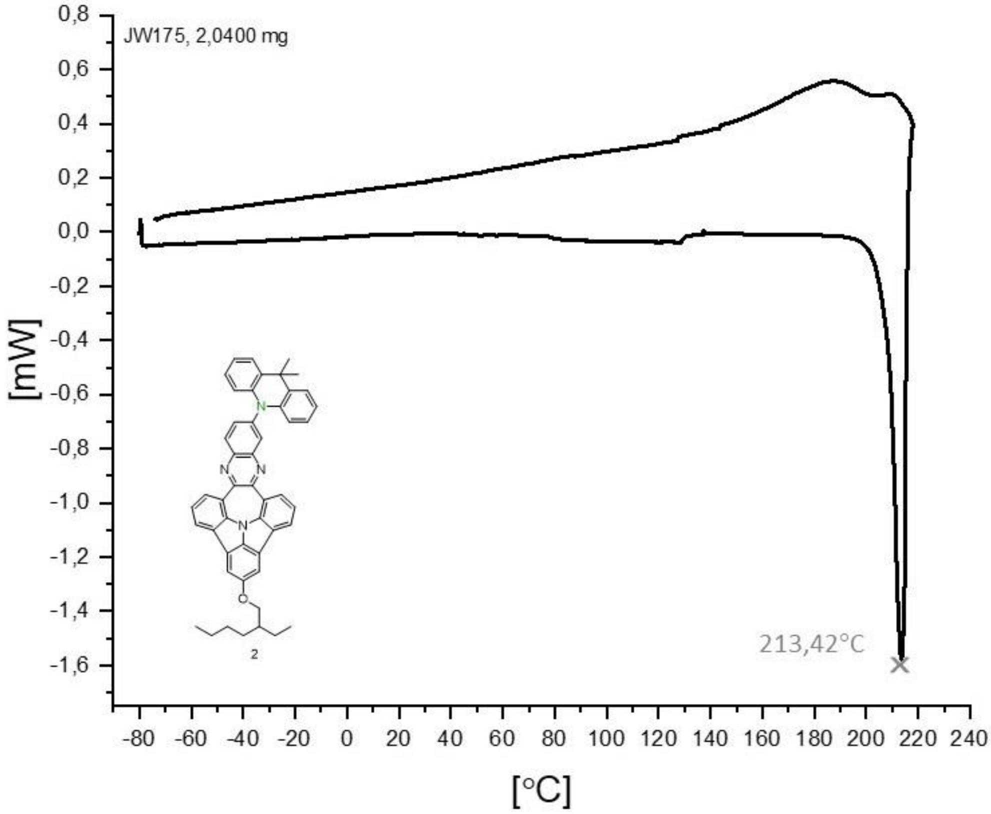

Figure S14. DSC heating/cooling (5ºC/ min) experiment for **2**.

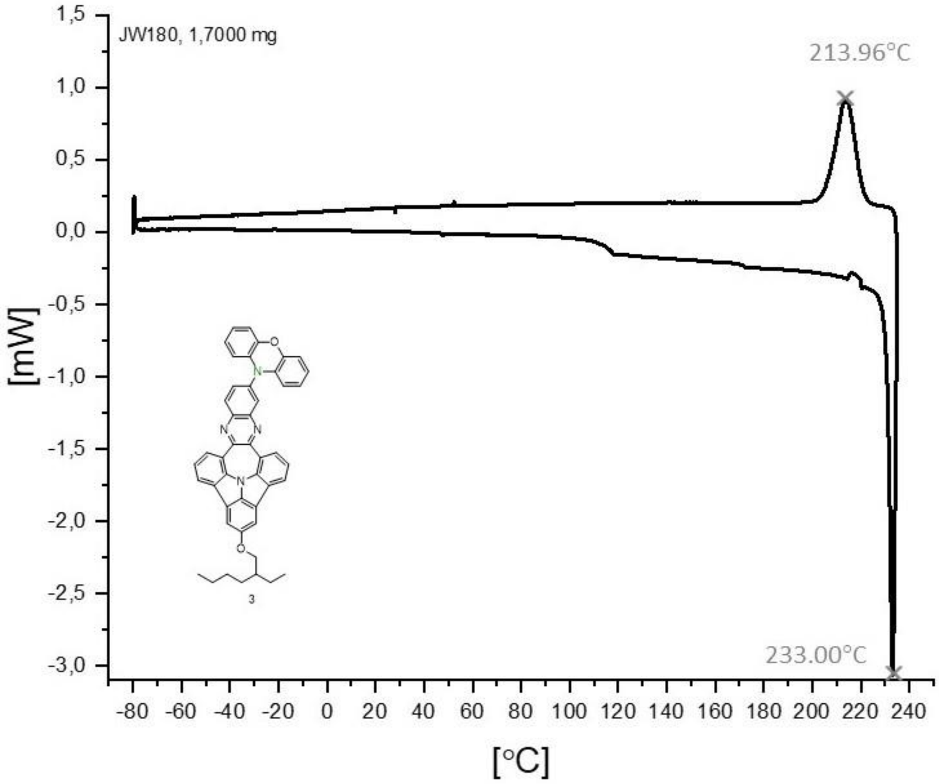

Figure S15. DSC heating/cooling (5ºC/ min) experiment for **3**.

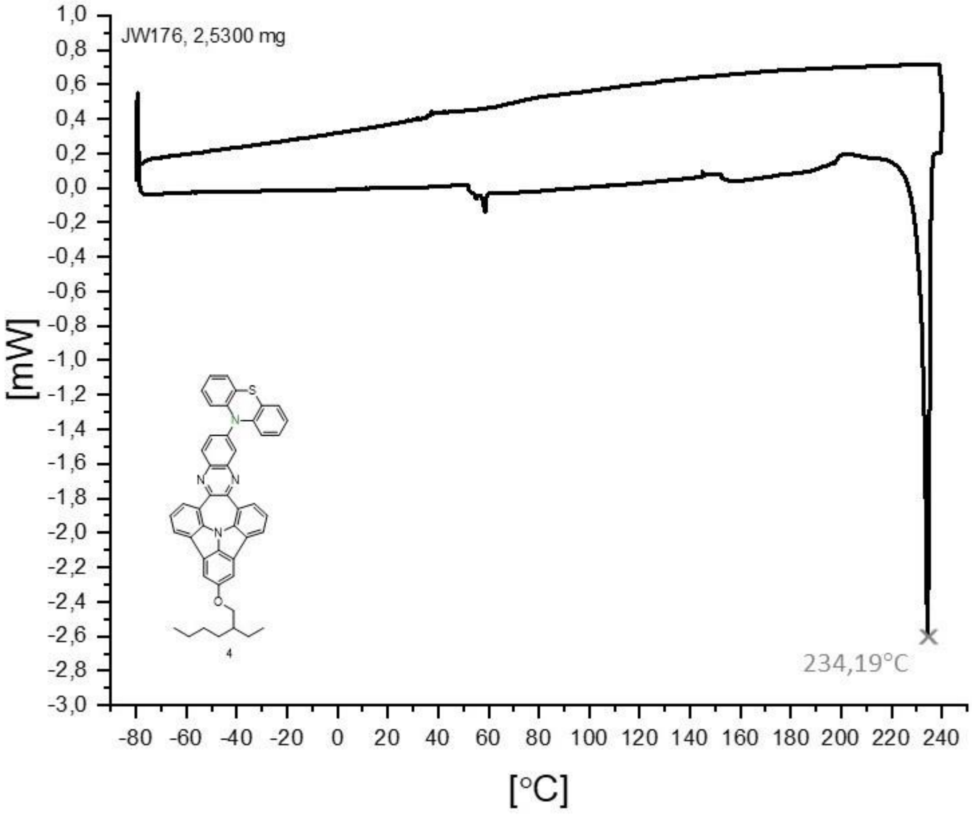

Figure S16. DSC heating/cooling (5ºC/ min) experiment for **4**.

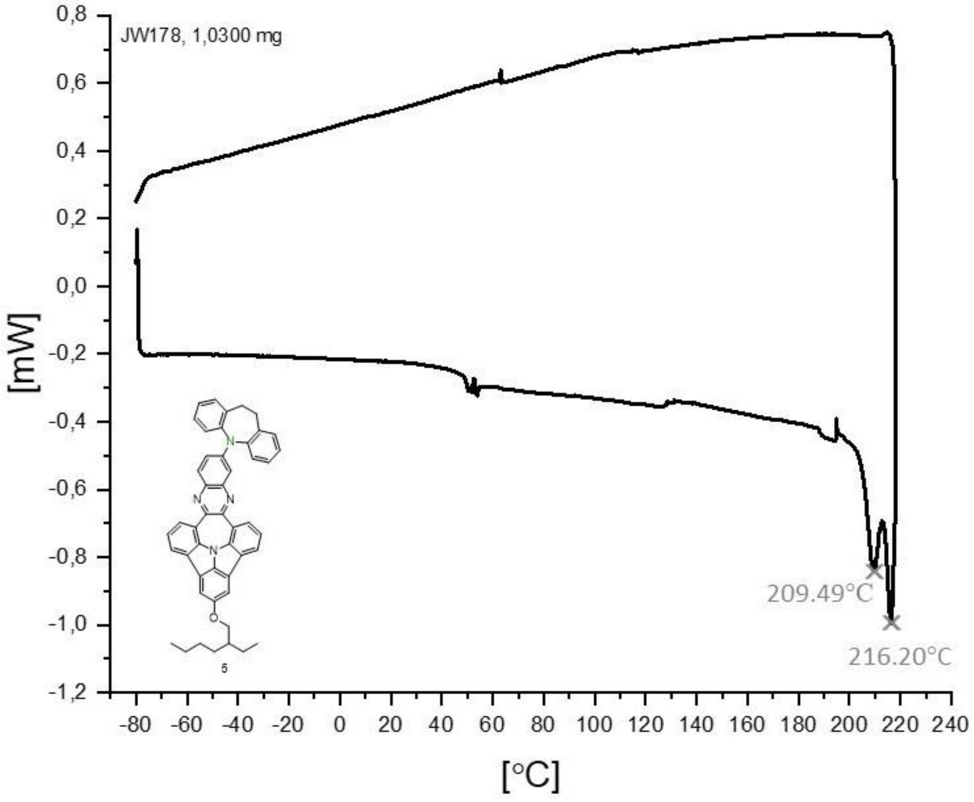

Figure S17. DSC heating/cooling (5ºC/ min) experiment for **5**.

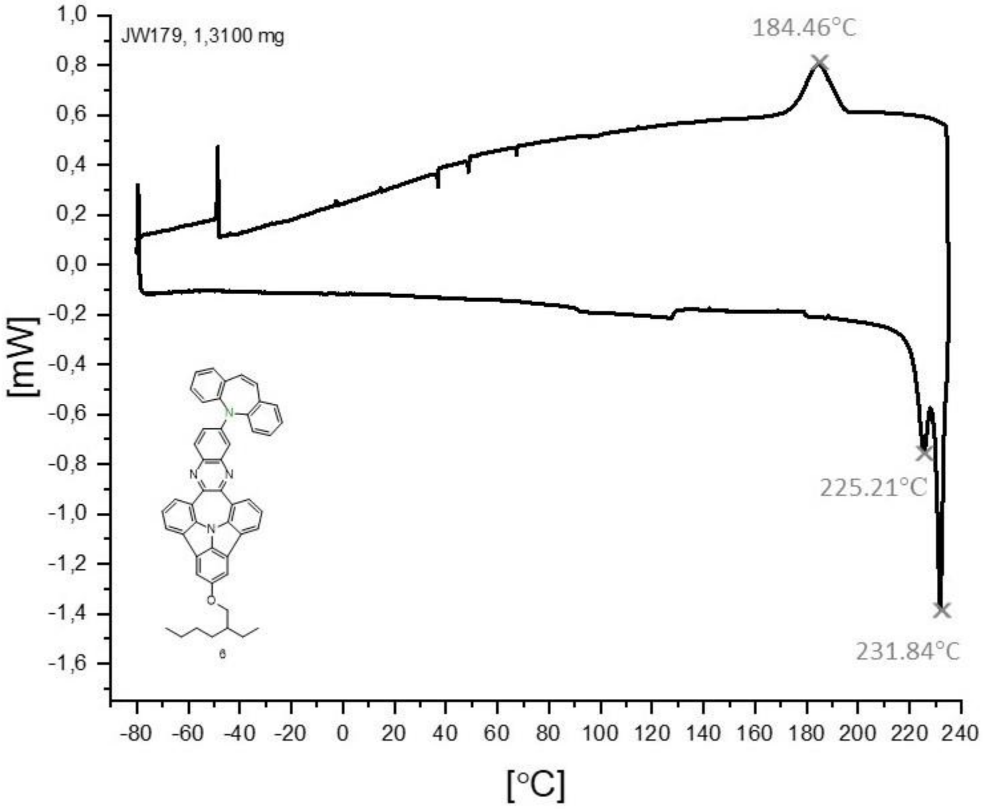

Figure S18. DSC heating/cooling (5ºC/ min) experiment for **6**.

SI-7 OLED devices

Table S3. Summary of the OLED performance based on compounds **1-6**.

**Compound**	**λ_{em}, nm**[a]	**EQE_{max} (%)**[b]	**EQE_{1000} (%)**[b]	**EQE_{5000} (%)**[b]	**EQE_{10000} (%)**[b]	**Lum_{max} (cd/m^2)**[b]
**1**	551	19.9	16.4	14.1	12.7	30 109
**2**	545	19.2	17.5	15.1	13.4	21 016
**3**	600	21.9	18.9	15.7	13.1	20 065
**4**	592	21.1	19.4	12.8	9.1	26 422
**5**	535	9.1	7.1	5.2	4.2	23 003
**6**	532	7.8	6.2	4.9	4.2	30 018

[a] Electroluminescence maximum; [b] OLED device maximum external quantum yield; [c] OLED device external quantum yield at 1000 cd/m^2; [d] OLED device external quantum yield at 5000 cd/m^2; [e] OLED device external quantum yield at 10000 cd/m^2; [f] Maximum of the luminance.

SI-8 NMR spectra of synthesized compounds

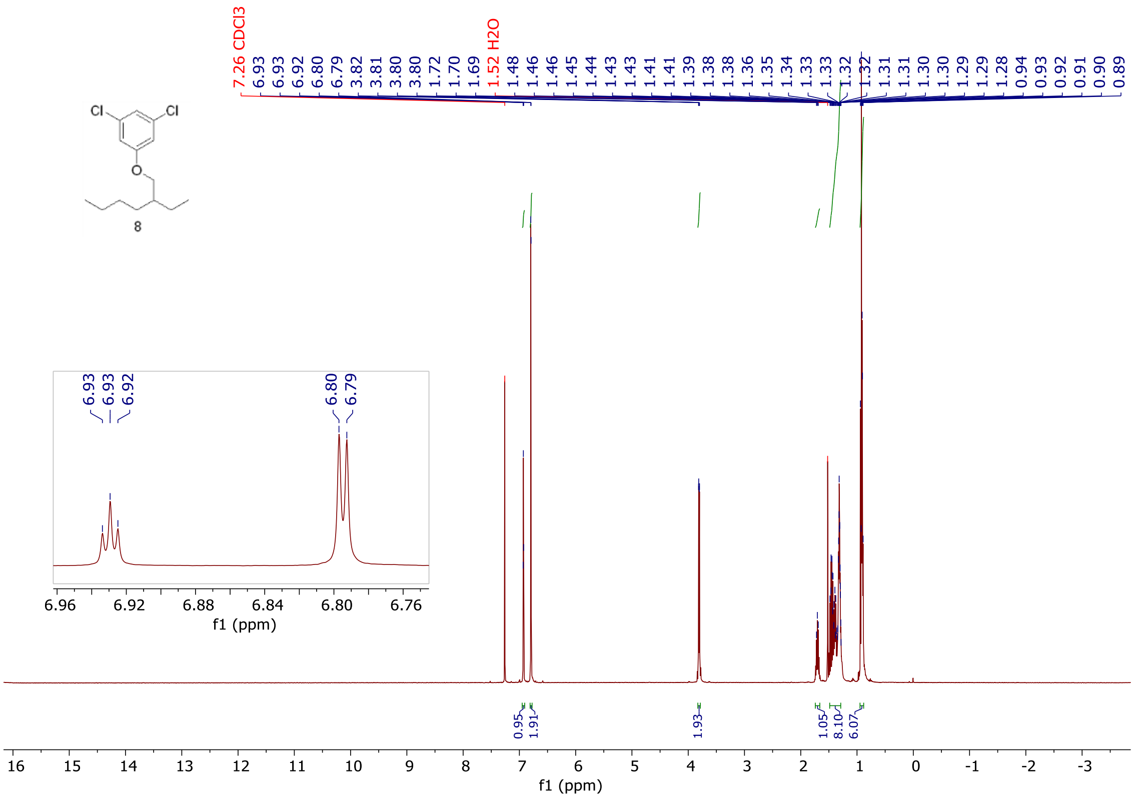

Figure S19. ^{1}H NMR spectrum of **8** (400 MHz, 300K, $CDCl_3$).

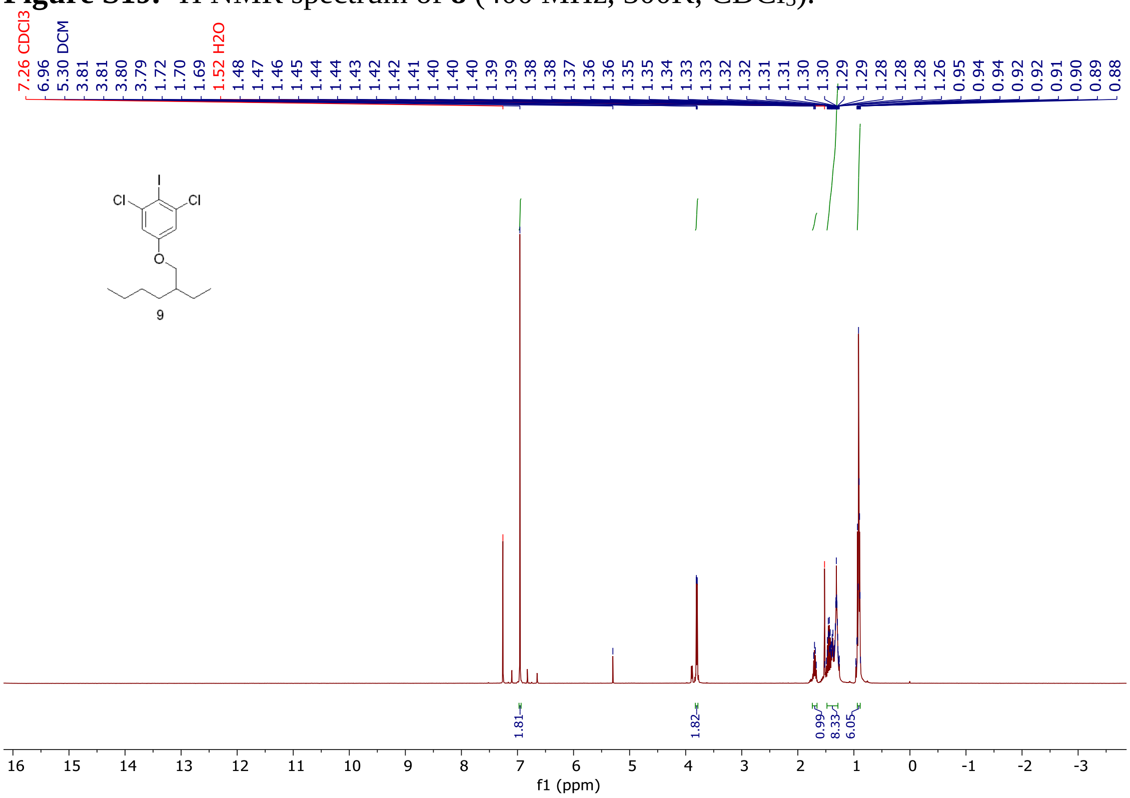

Figure S20. ^{1}H NMR spectrum of **9** (400 MHz, 300K, $CDCl_3$).

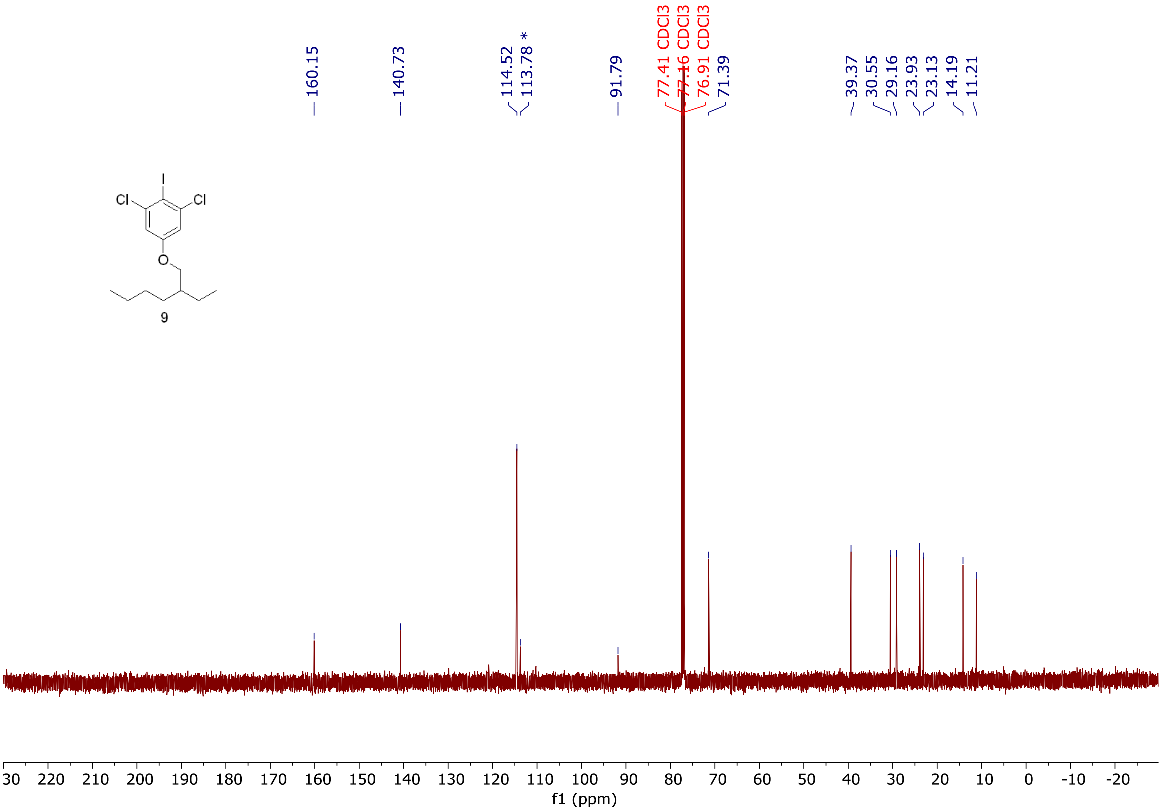

Figure S21. ^{13}C NMR spectrum of **9** (126 MHz, 300K, $CDCl_3$).

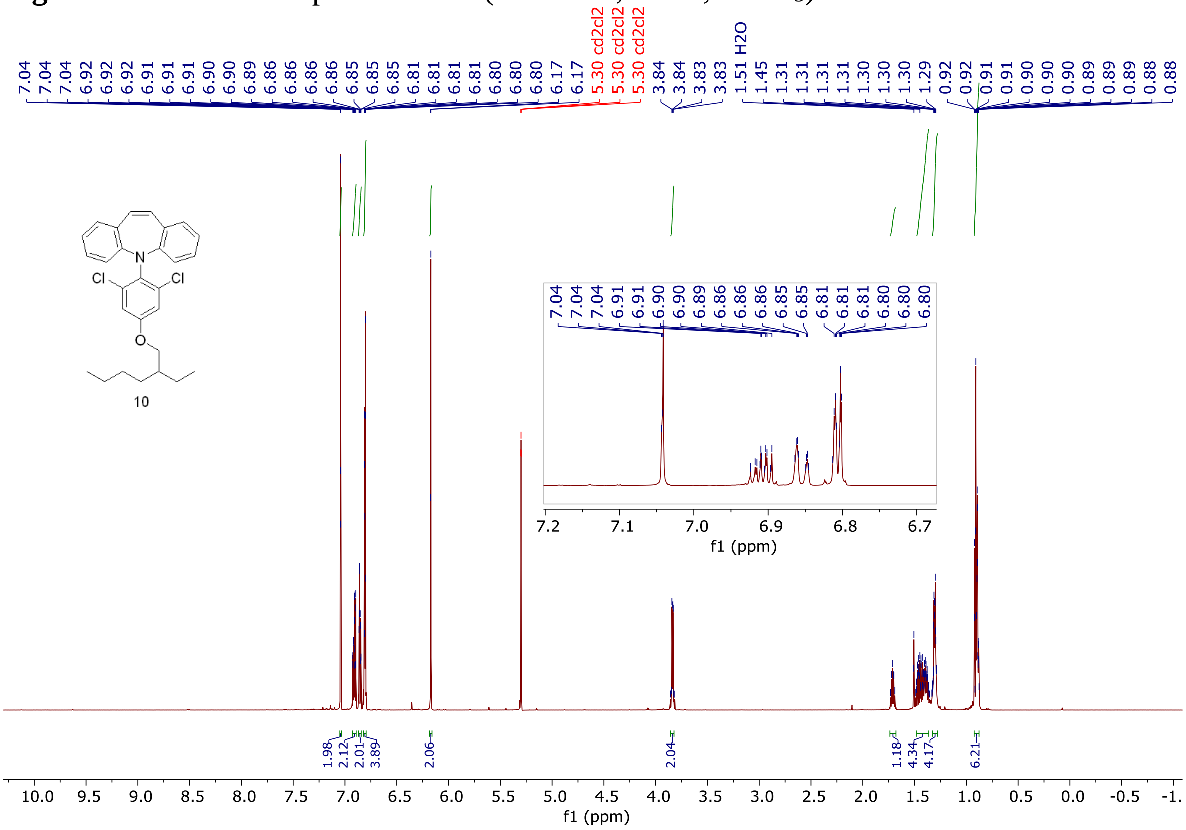

Figure S22. ^{1}H NMR spectrum of **10** (600 MHz, 300K, CD_2Cl_2).

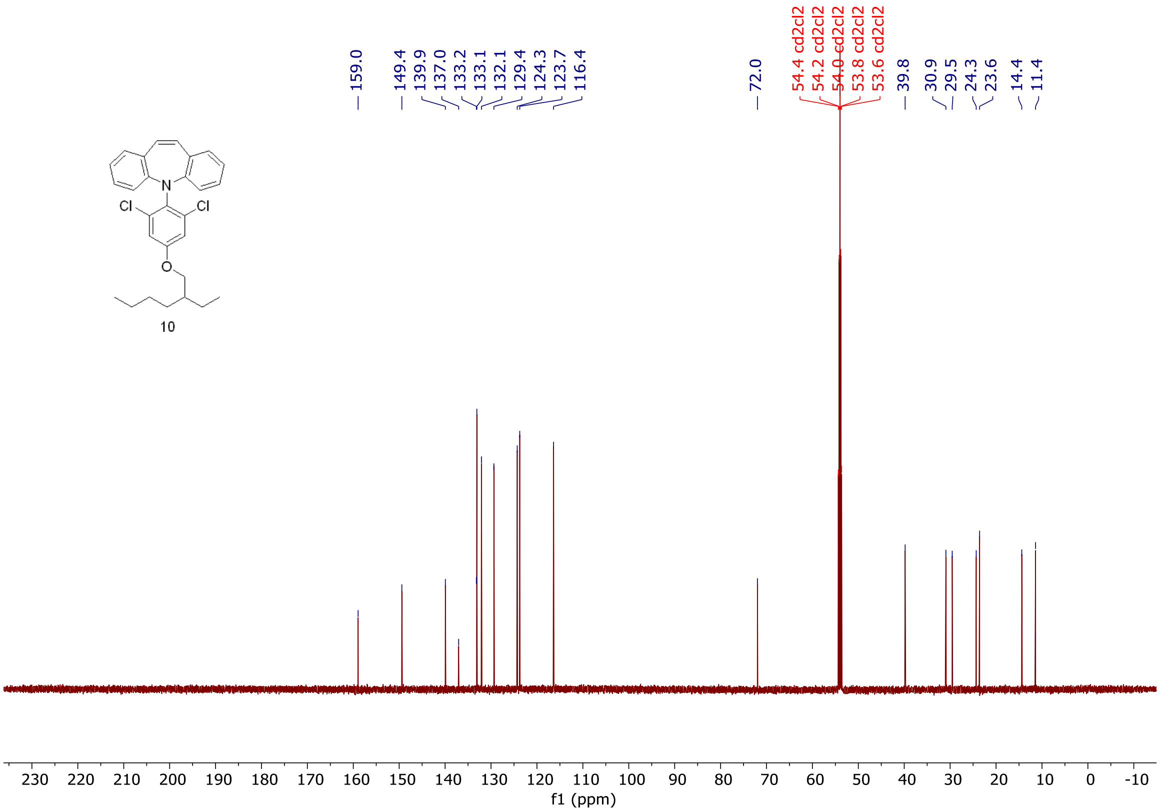

Figure S23. ^{13}C NMR spectrum of **10** (151 MHz, 300K, CD_2Cl_2).

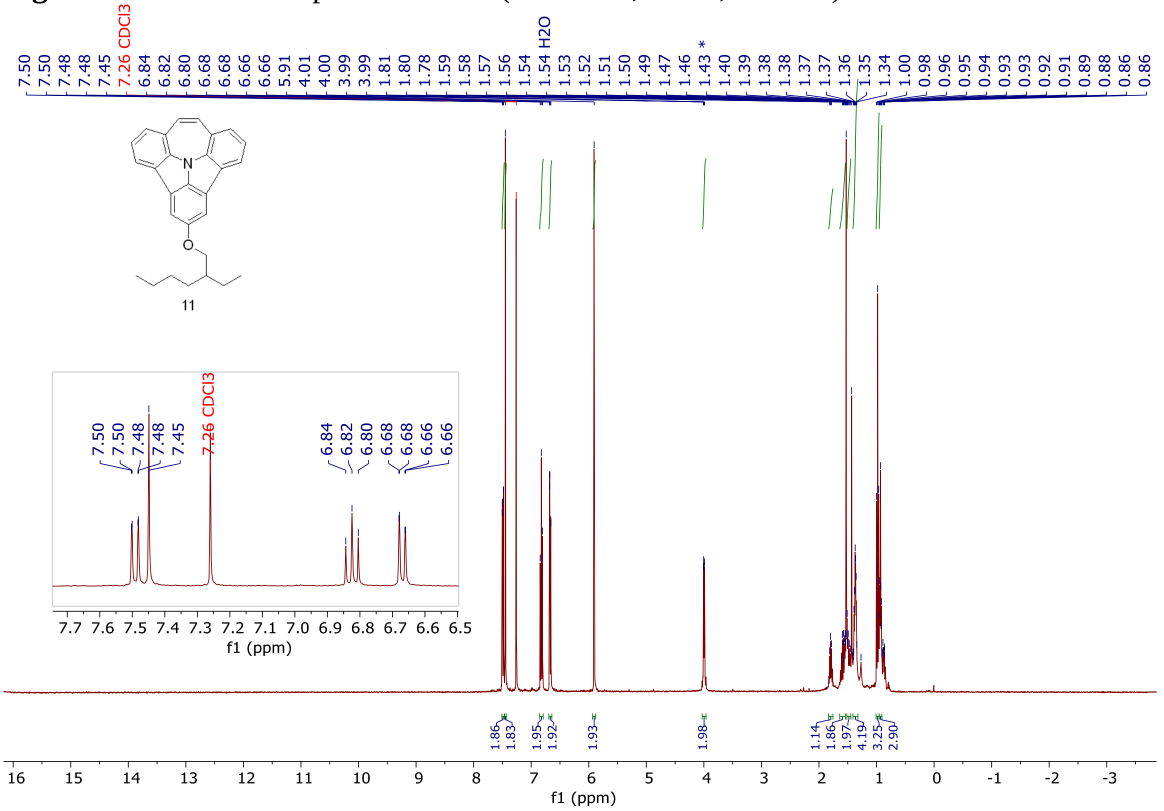

Figure S24. ^{1}H NMR spectrum of **11** (400 MHz, 300K, $CDCl_3$).

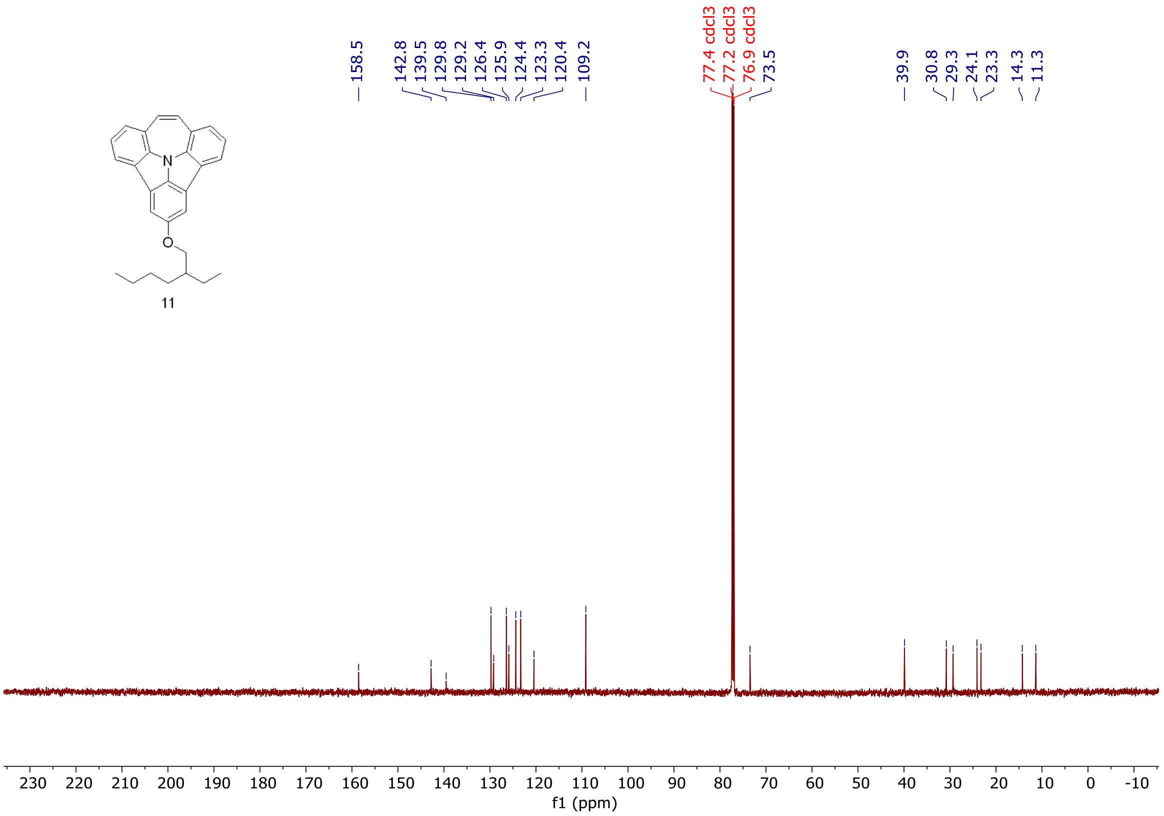

Figure S25. ^{13}C NMR spectrum of **11** (151 MHz, 300K, $CDCl_3$).

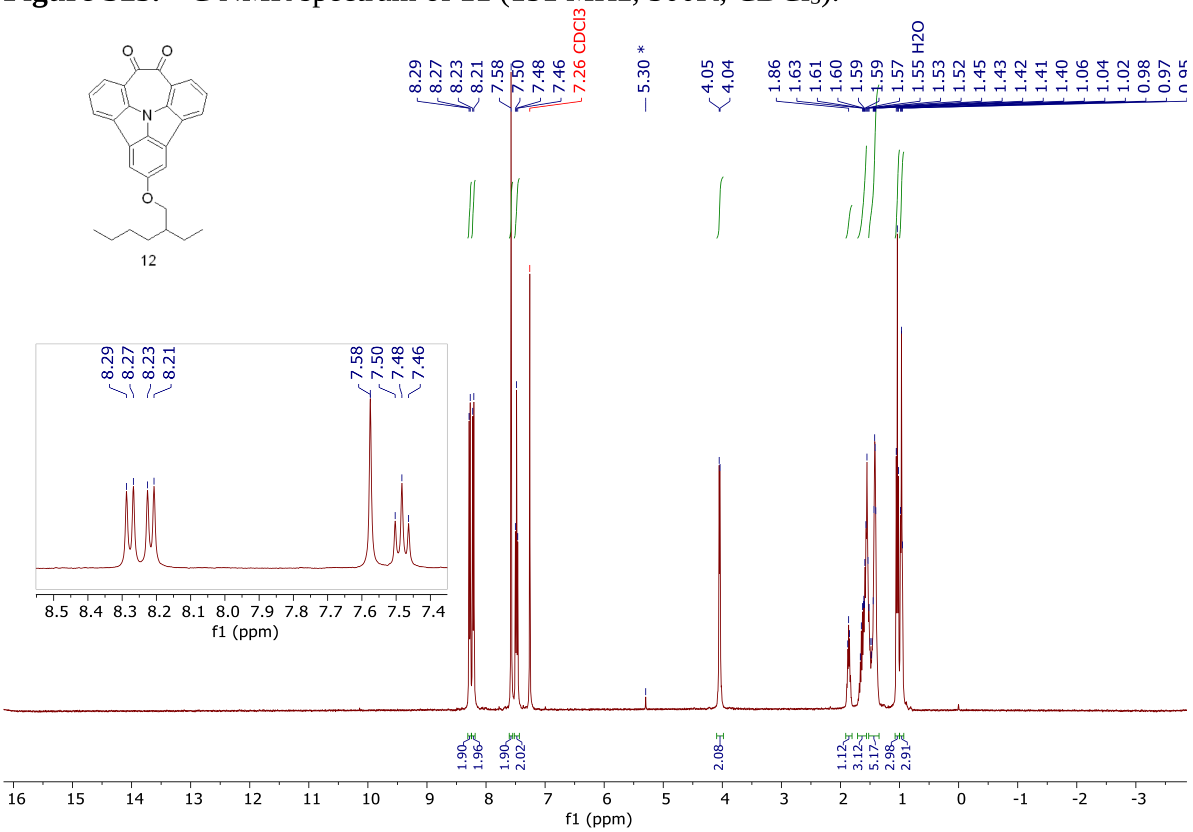

Figure S26. ^{1}H NMR spectrum of **12** (400 MHz, 300K, $CDCl_3$).

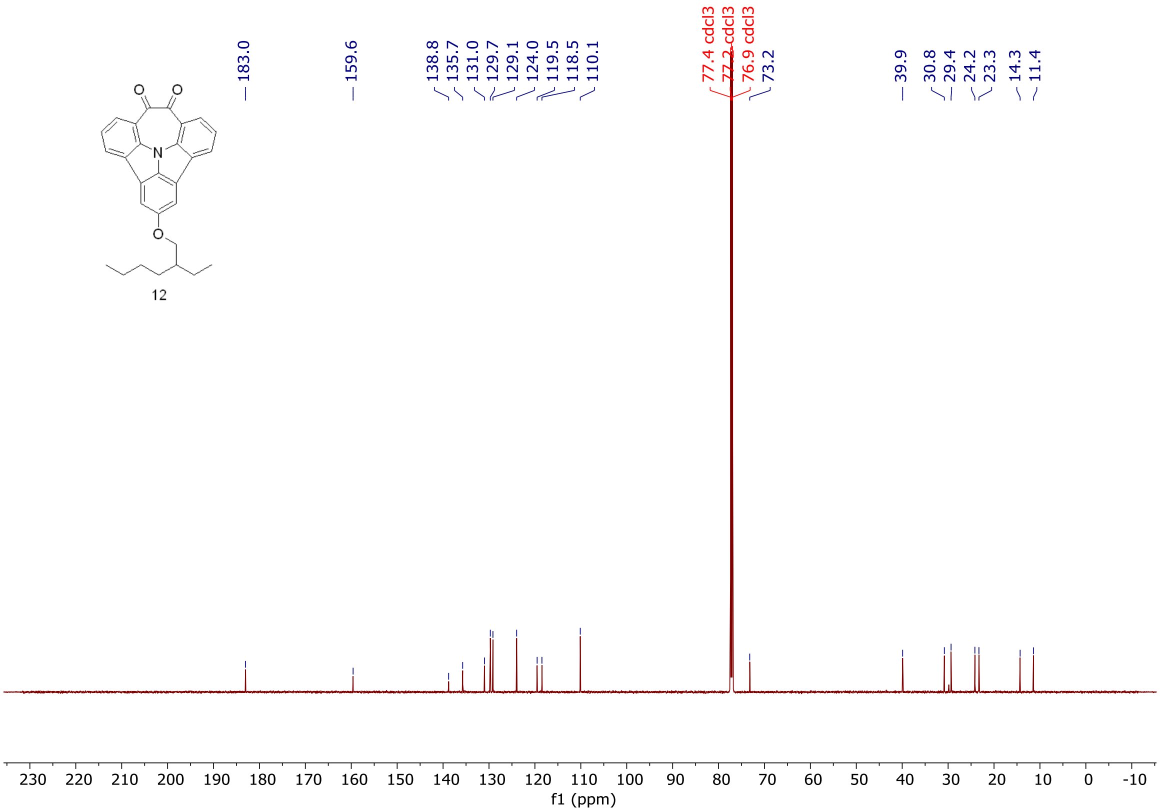

Figure S27. ^{13}C NMR spectrum of **12** (151 MHz, 300K, $CDCl_3$).

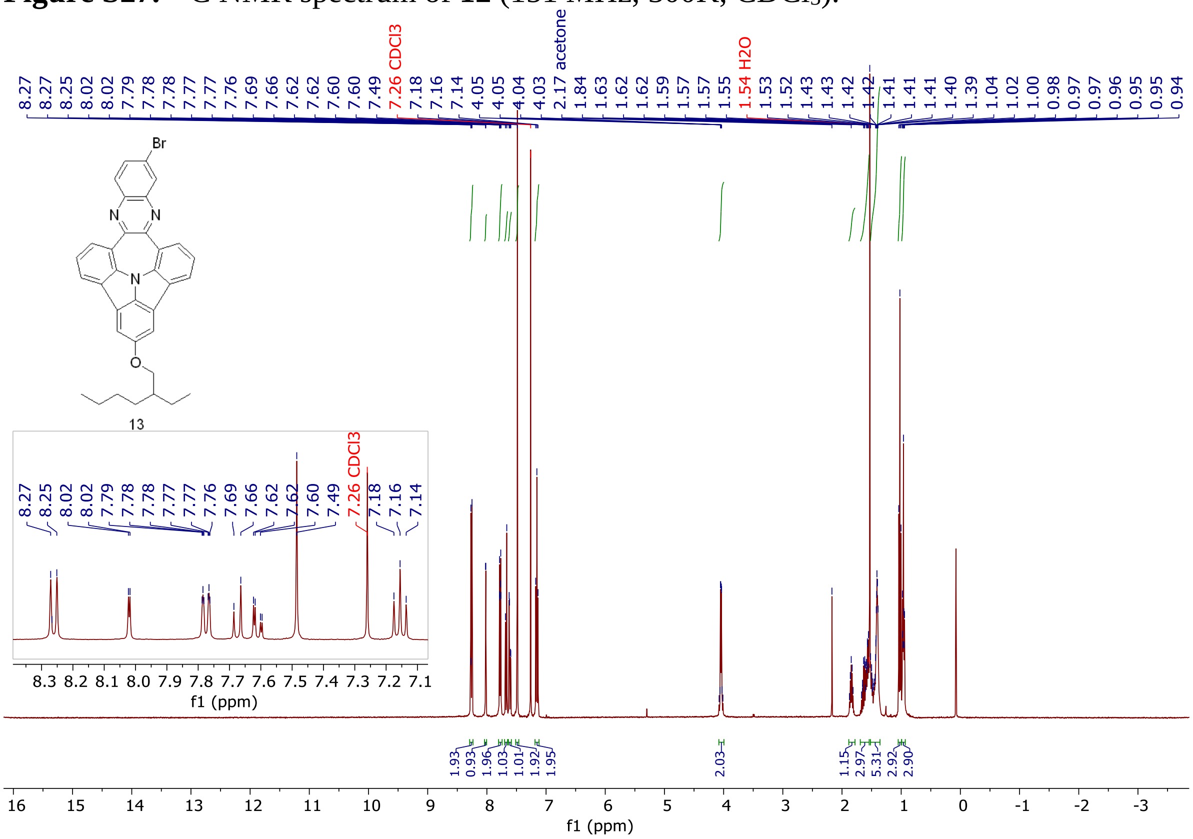

Figure S28. ^{1}H NMR spectrum of **13** (400 MHz, 300K, $CDCl_3$).

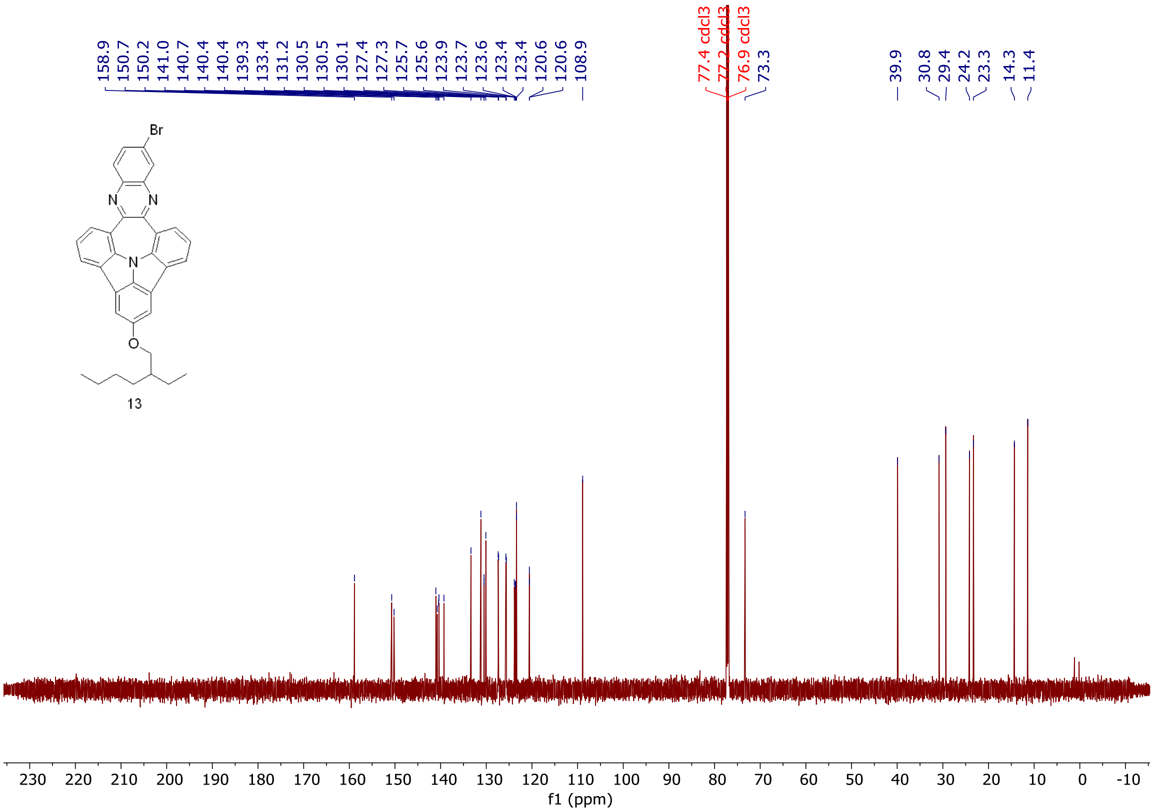

Figure S29. ^{13}C NMR spectrum of **13** (151 MHz, 300K, $CDCl_3$).

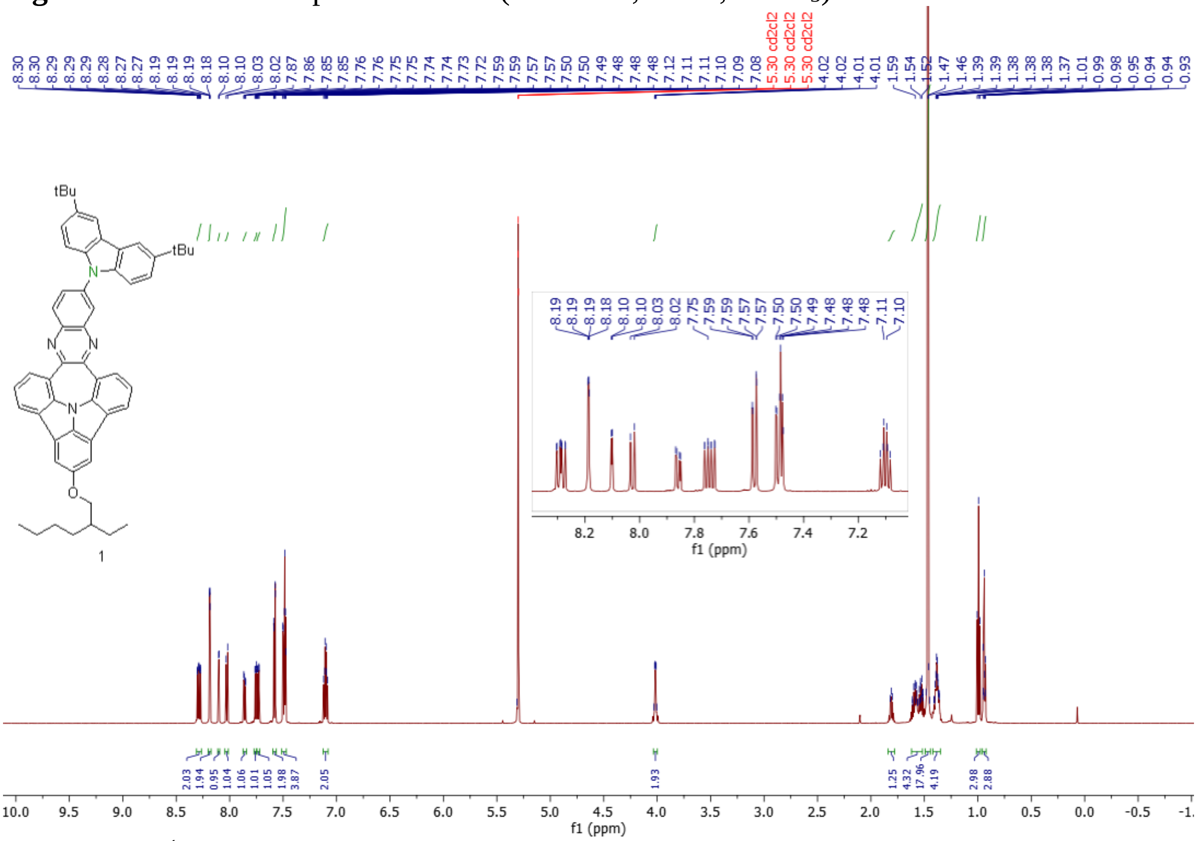

Figure S30. ^{1}H NMR spectrum of **1** (600 MHz, 300K, CD_2Cl_2).

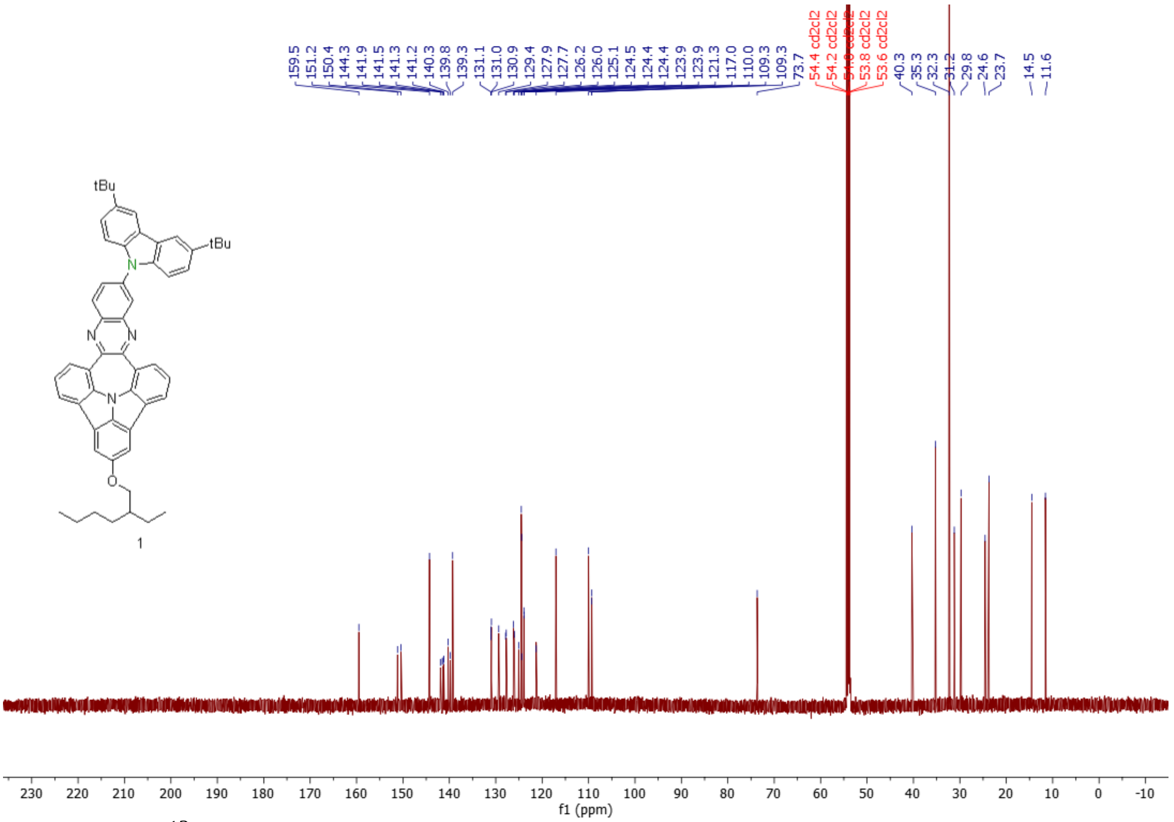

Figure S31. ^{13}C NMR spectrum of **1** (151 MHz, 300K, CD_2Cl_2).

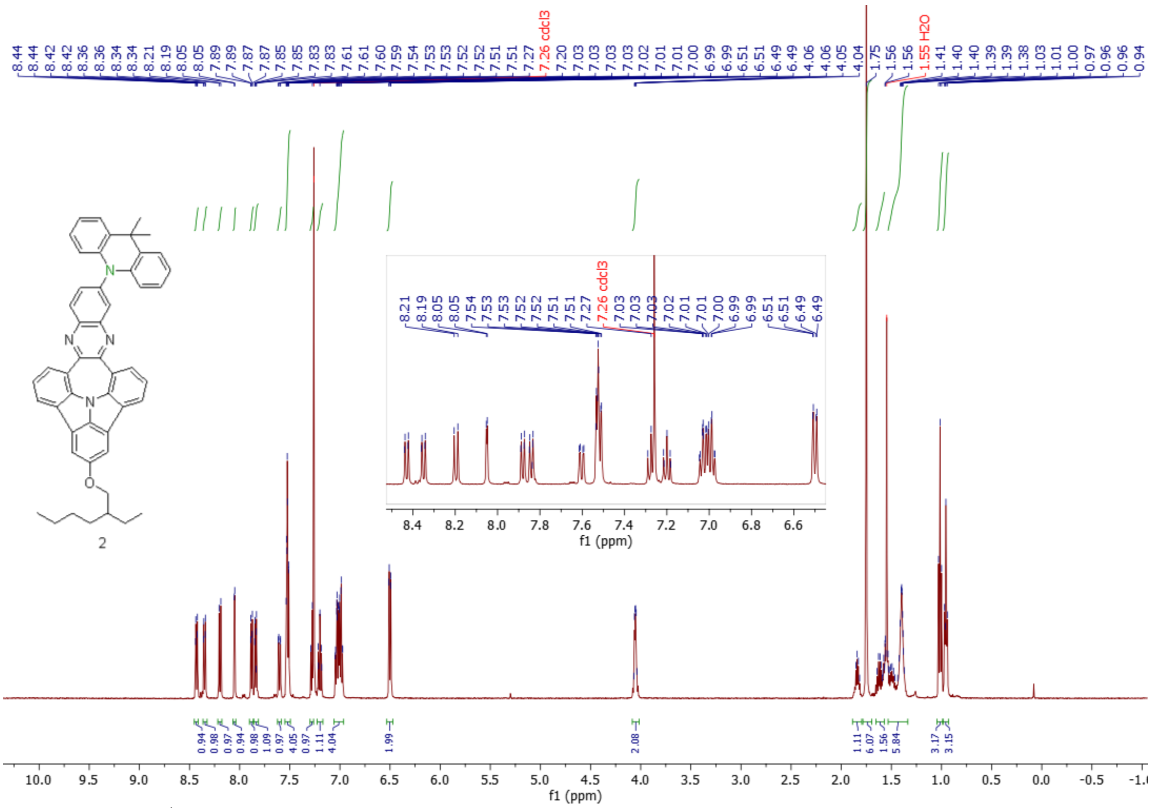

Figure S32. ^{1}H NMR spectrum of **2** (500 MHz, 300K, $CDCl_3$).

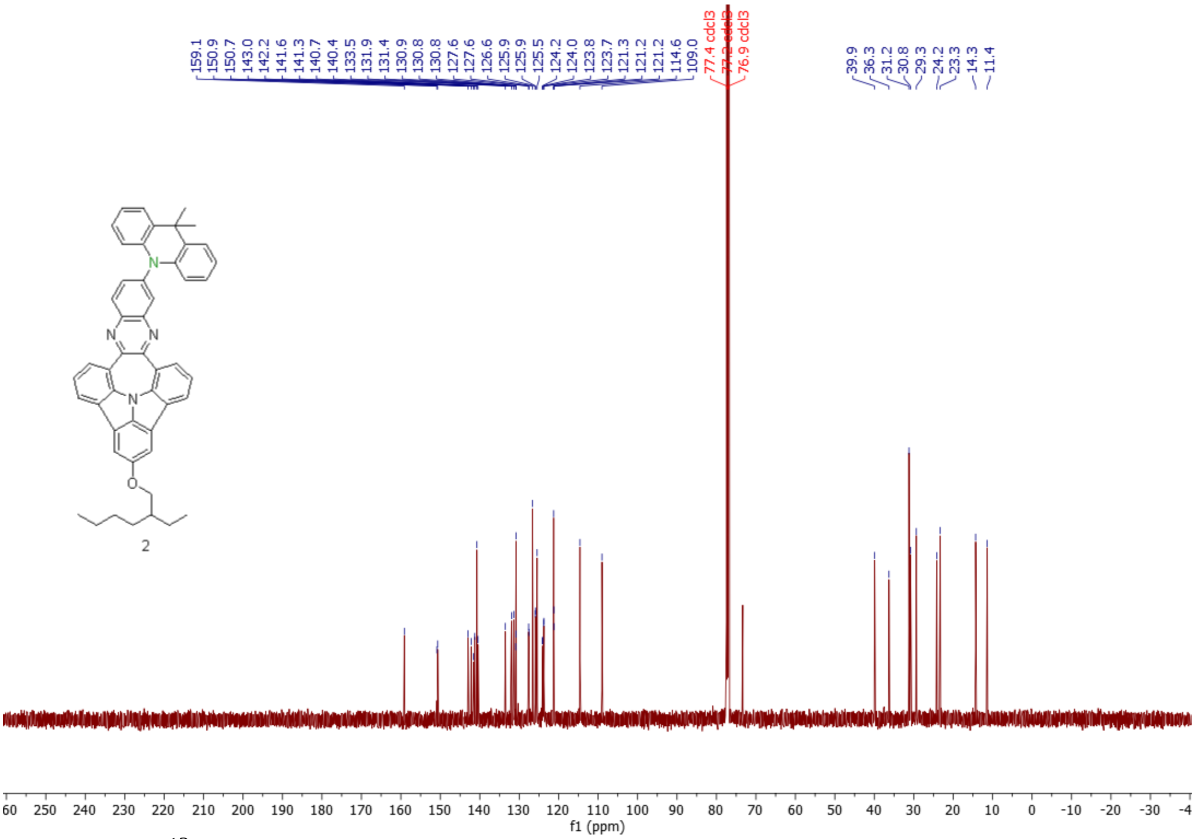

Figure S33. ^{13}C NMR spectrum of **2** (126 MHz, 300K, $CDCl_3$).

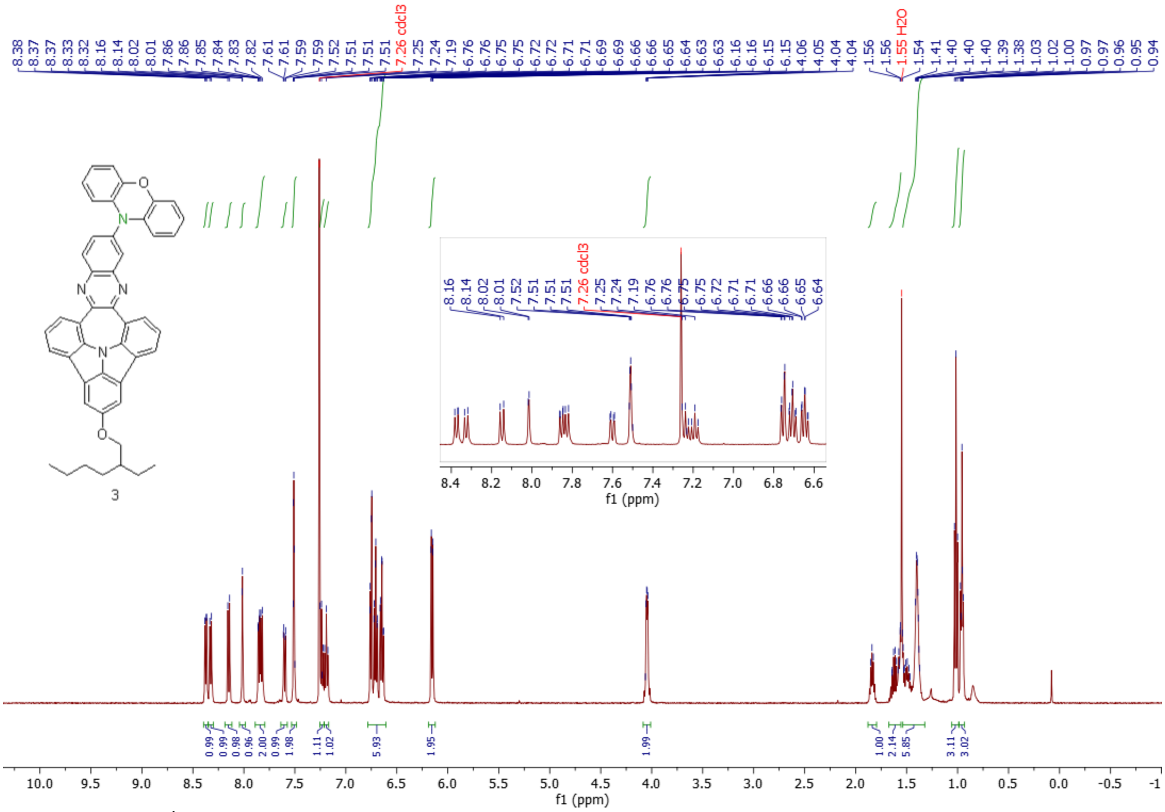

Figure S34. ^{1}H NMR spectrum of **3** (500 MHz, 300K, $CDCl_3$).

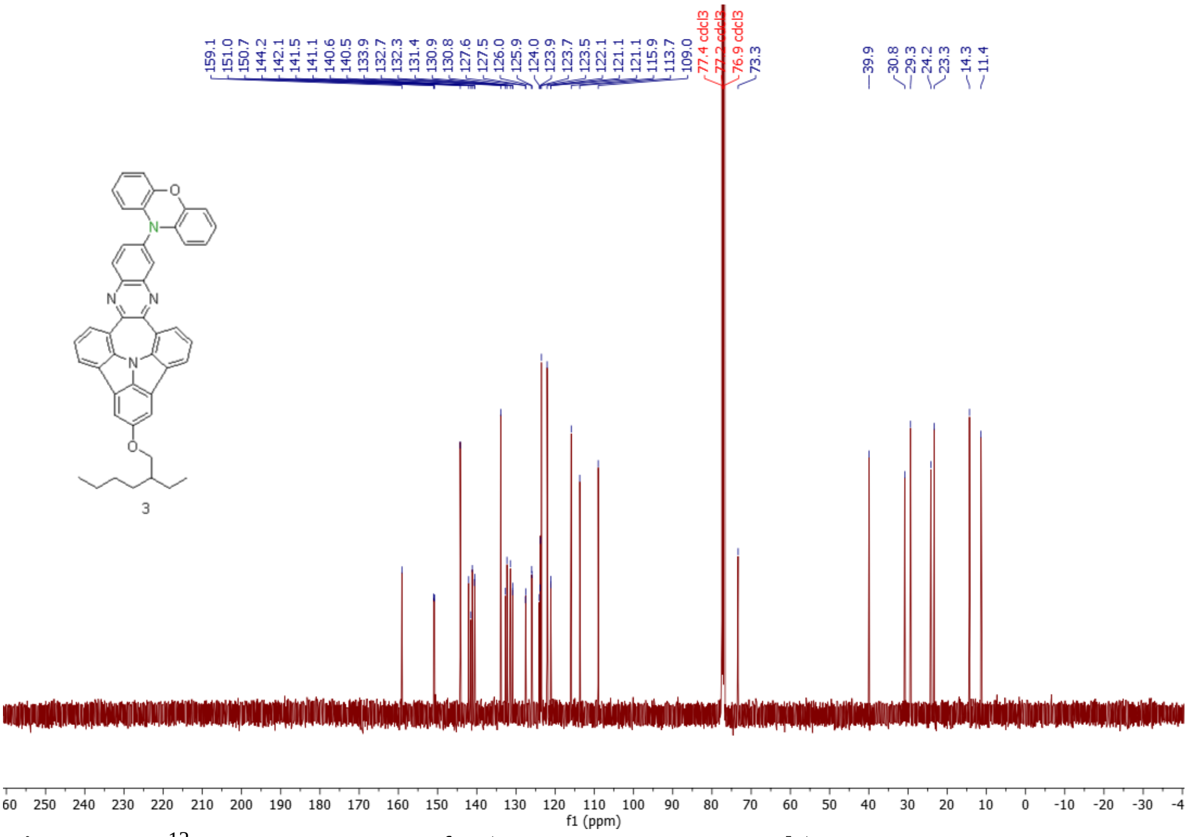

Figure S35. ^{13}C NMR spectrum of **3** (126 MHz, 300K, $CDCl_3$).

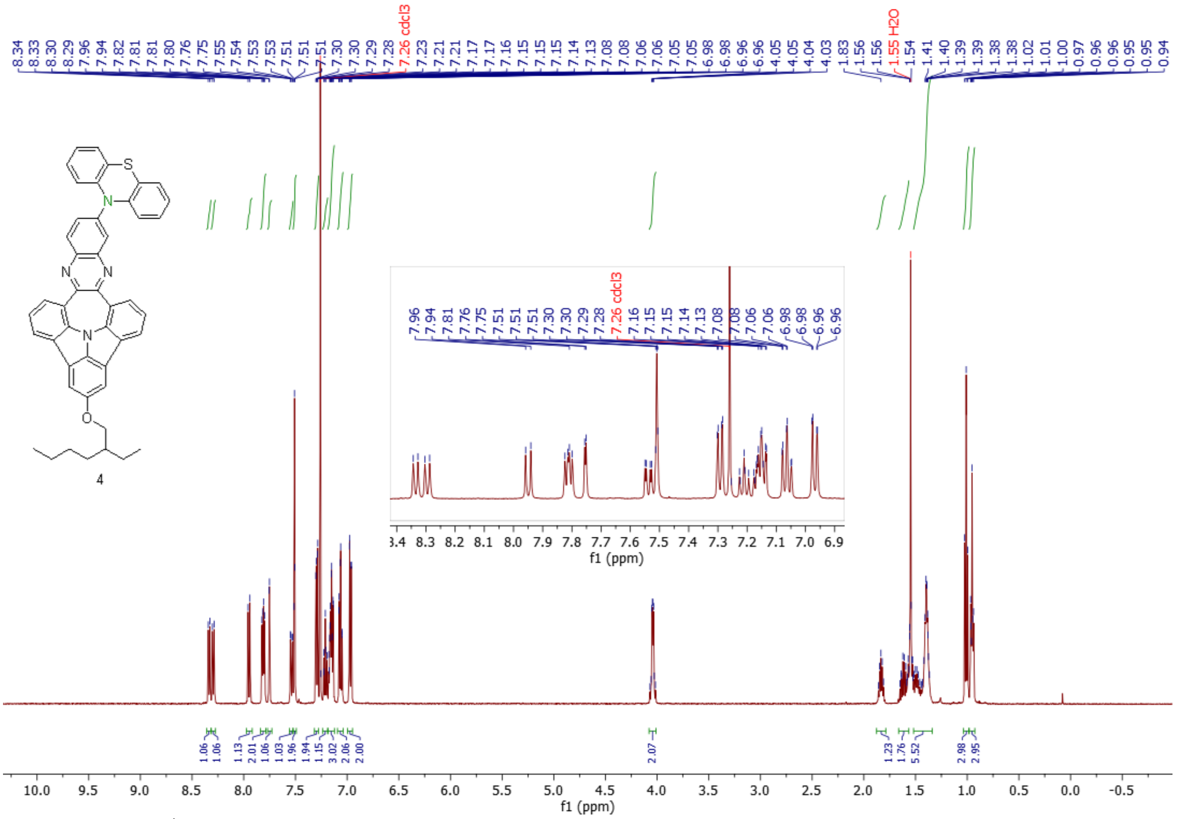

Figure S36. ^{1}H NMR spectrum of **4** (500 MHz, 300K, $CDCl_3$).

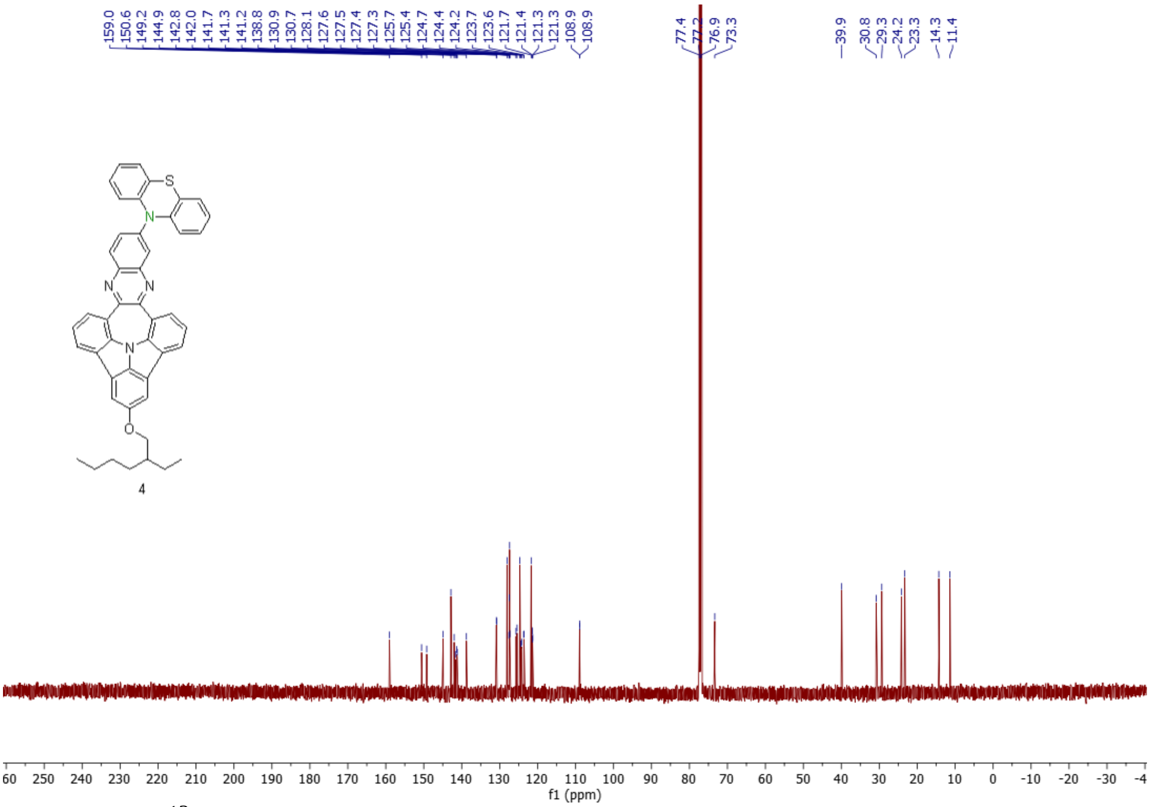

Figure S37. ^{13}C NMR spectrum of **4** (126 MHz, 300K, $CDCl_3$).

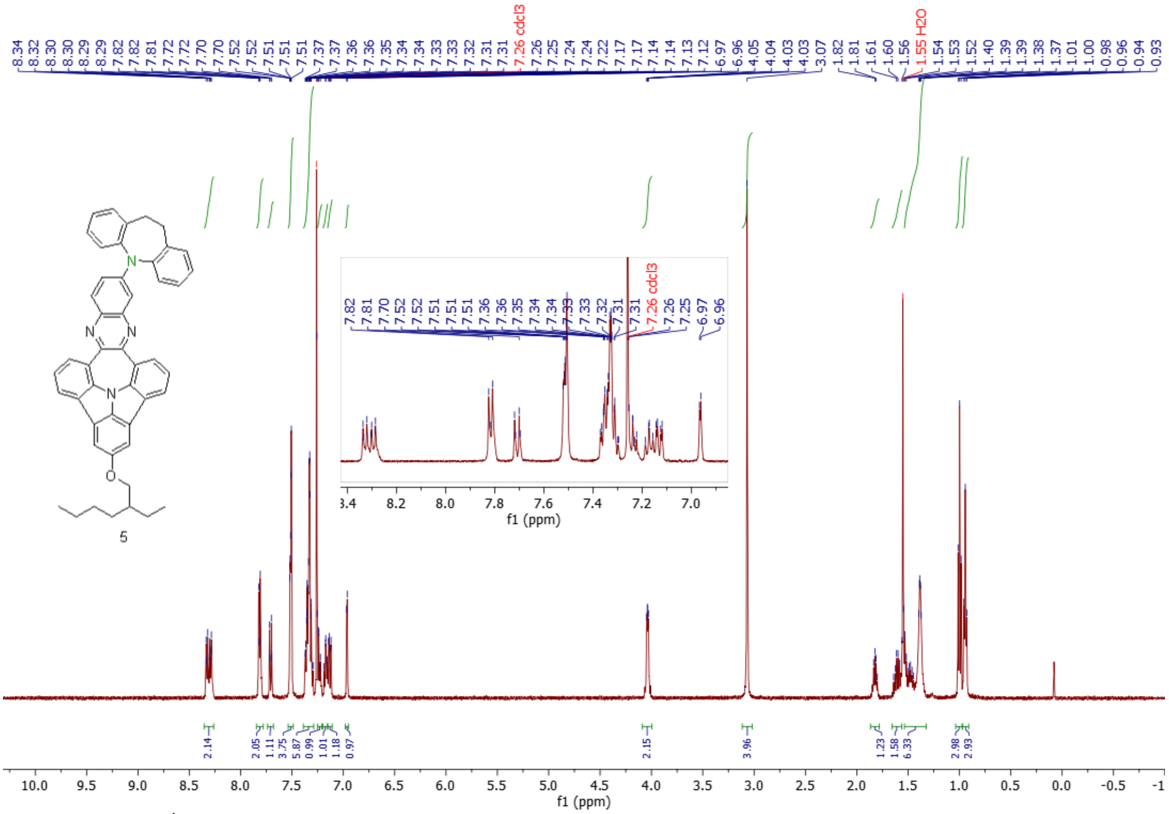

Figure S38. ^{1}H NMR spectrum of **5** (500 MHz, 300K, $CDCl_3$).

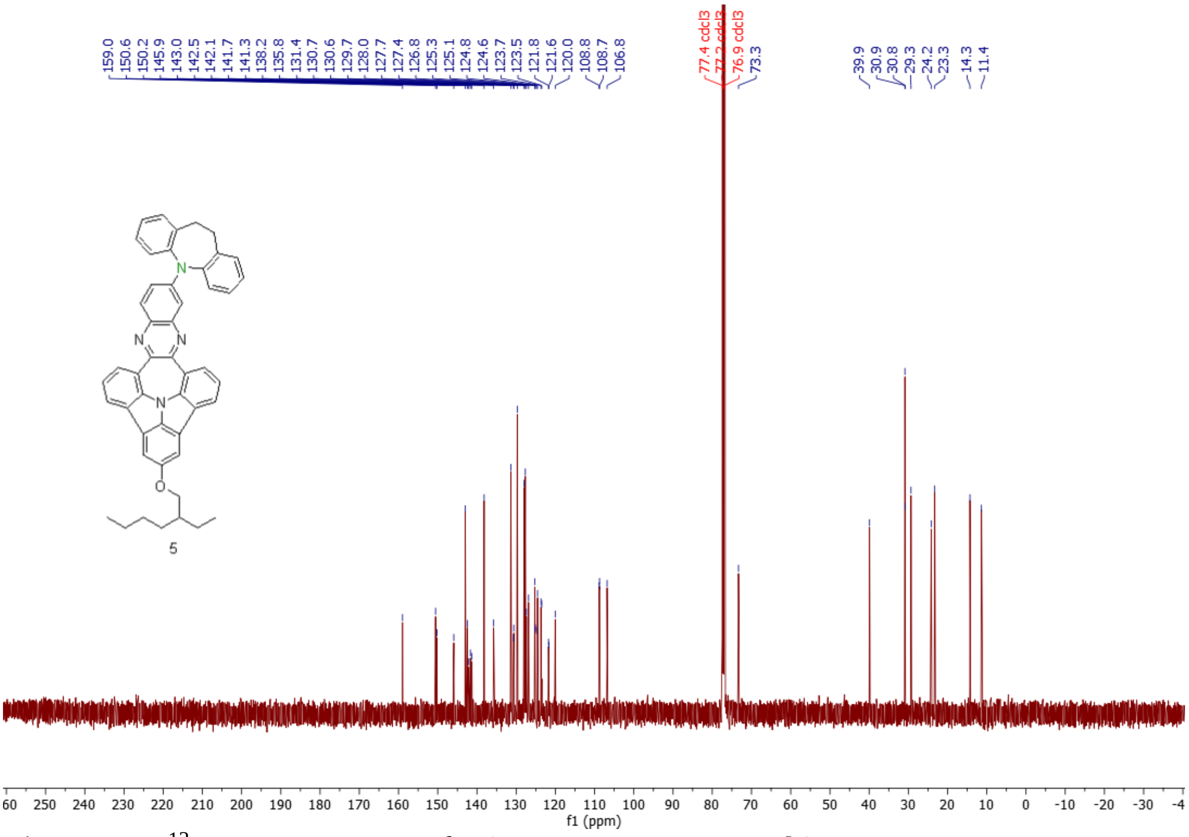

Figure S39. ^{13}C NMR spectrum of **5** (126 MHz, 300K, $CDCl_3$).

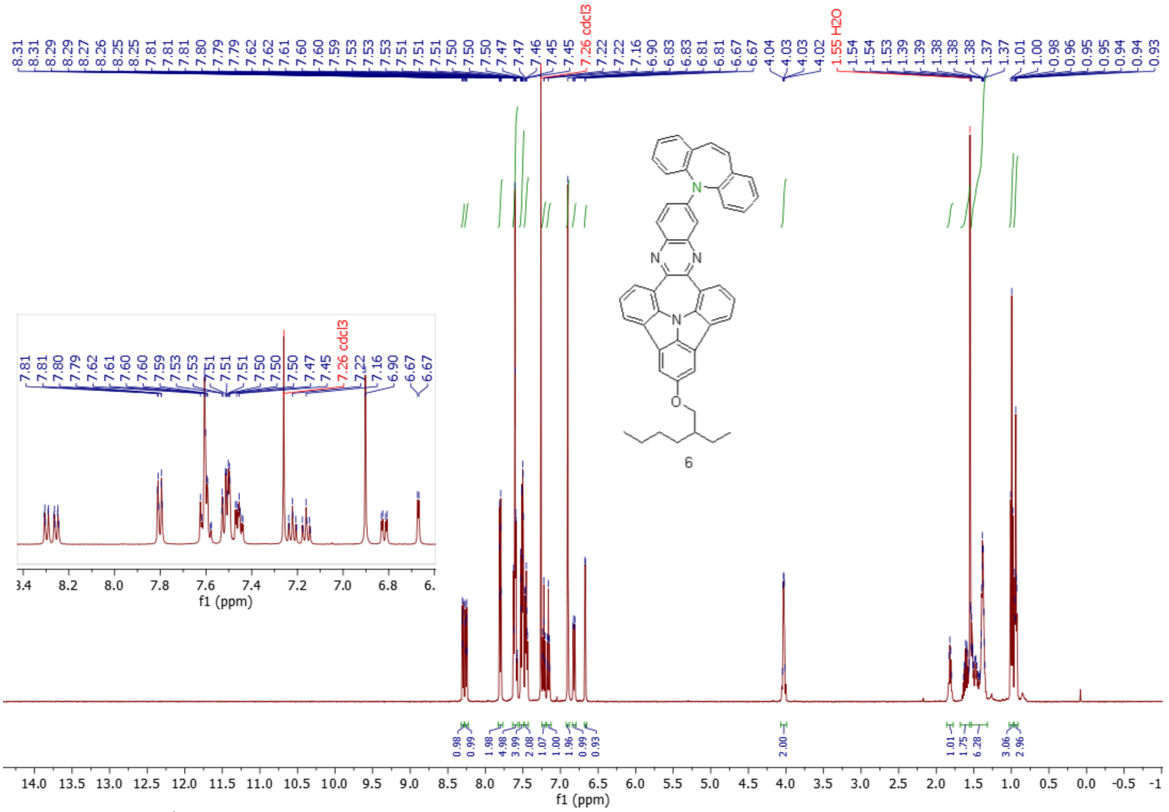

Figure S40. ^{1}H NMR spectrum of **6** (500 MHz, 300K, $CDCl_3$).

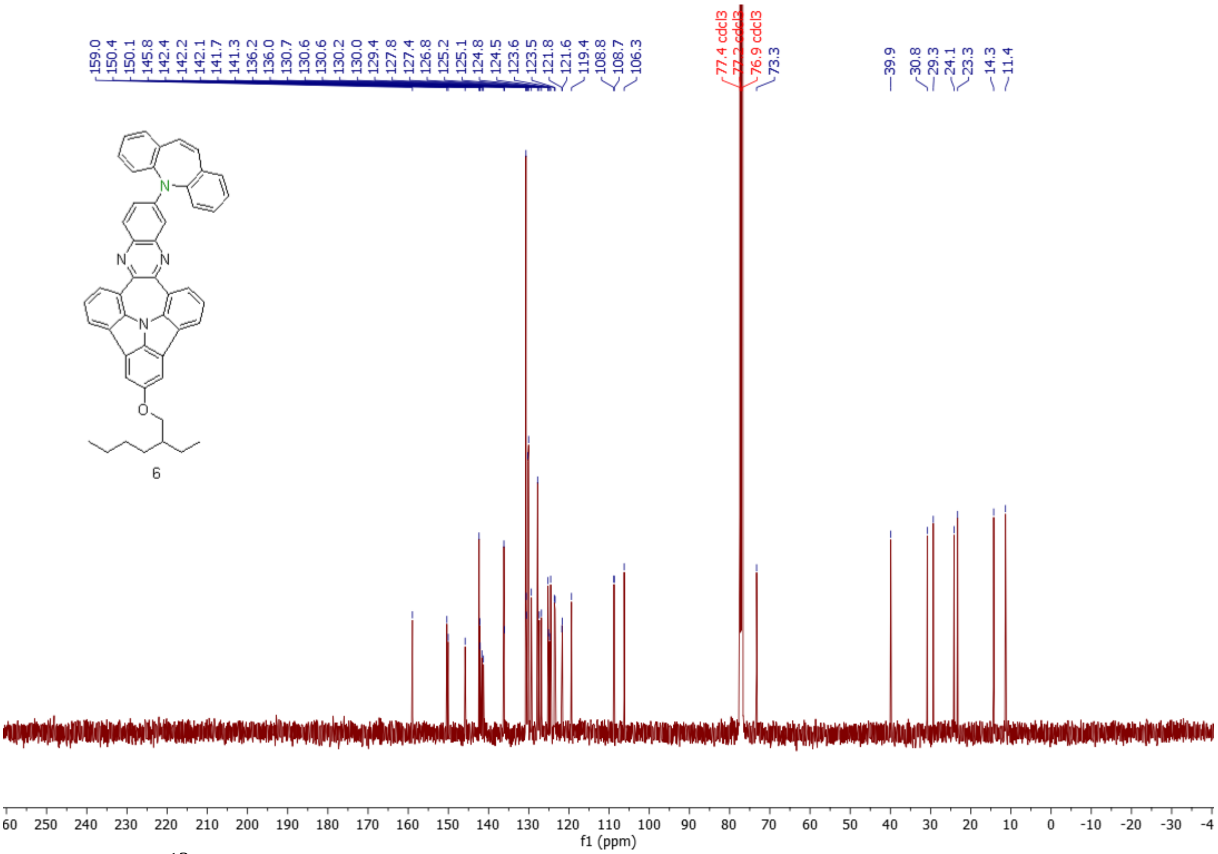

Figure S41. ^{13}C NMR spectrum of **6** (126 MHz, 300K, $CDCl_3$).

SI-9 HPLC spectra of final compounds

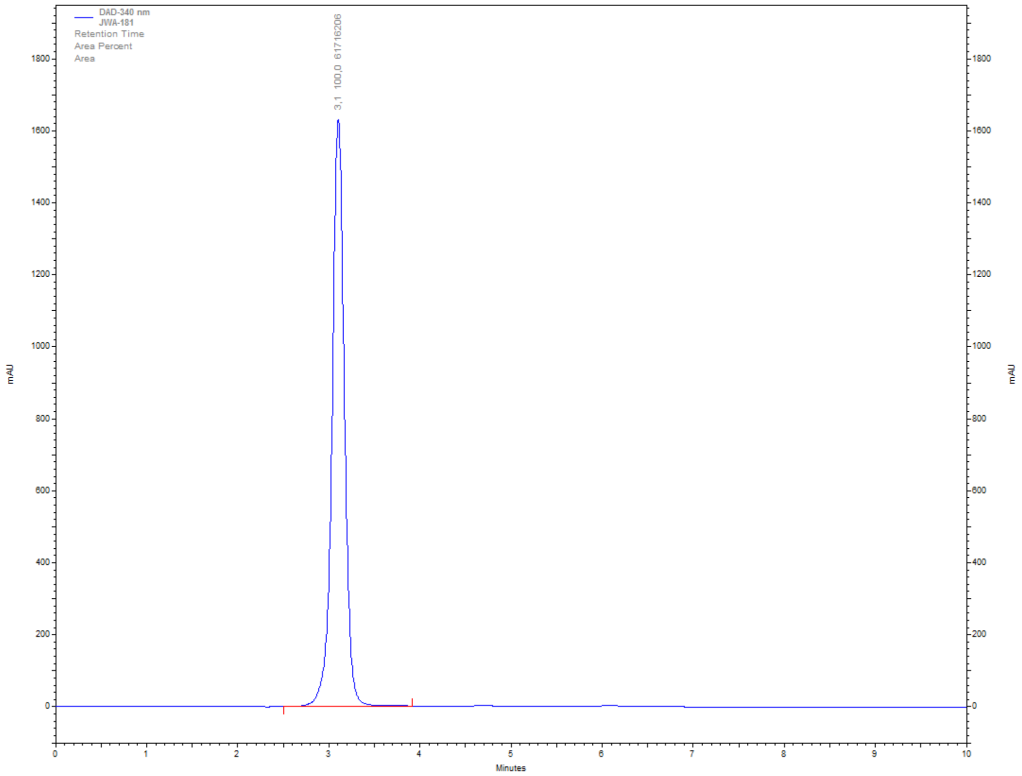

Figure S42. HPLC spectrum of **1**.

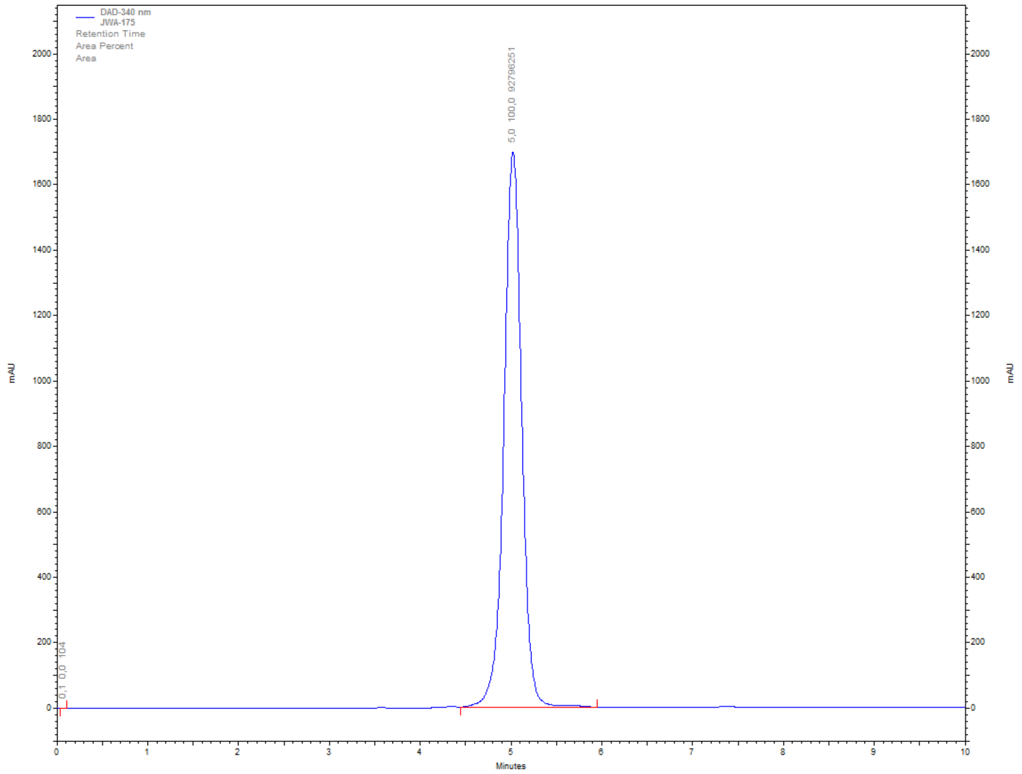

Figure S43. HPLC spectrum of **2**.

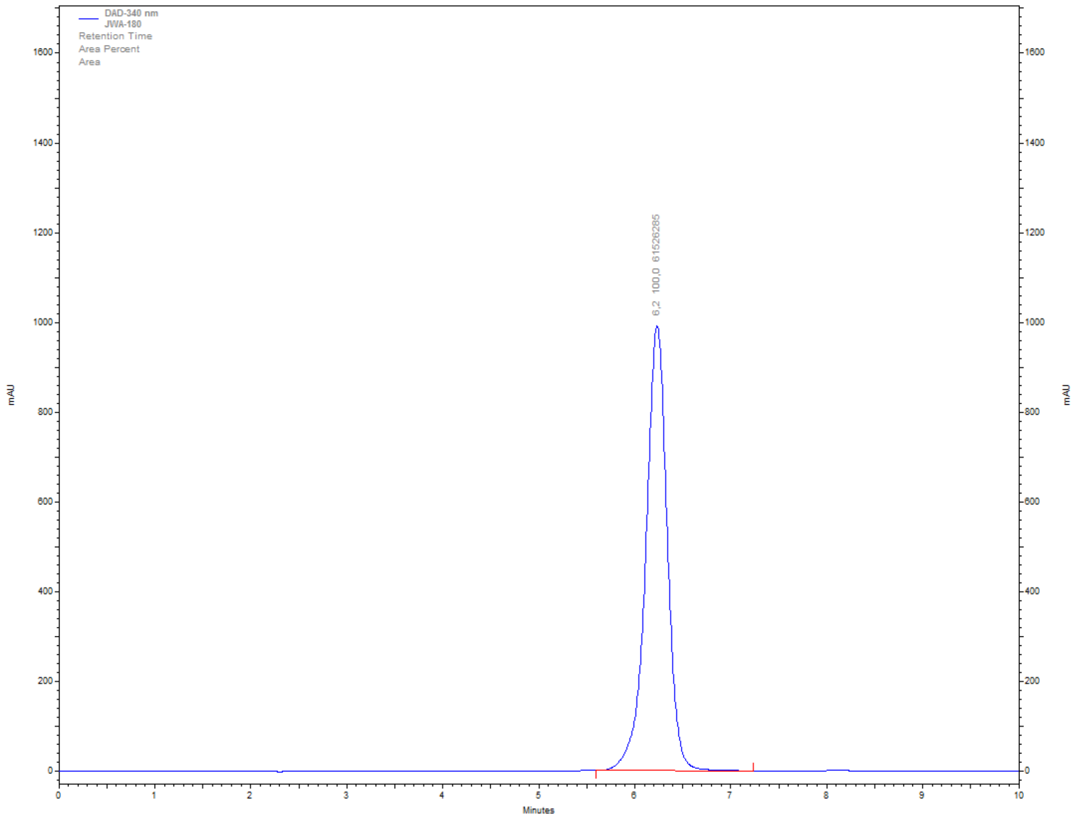

Figure S44. HPLC spectrum of **3**.

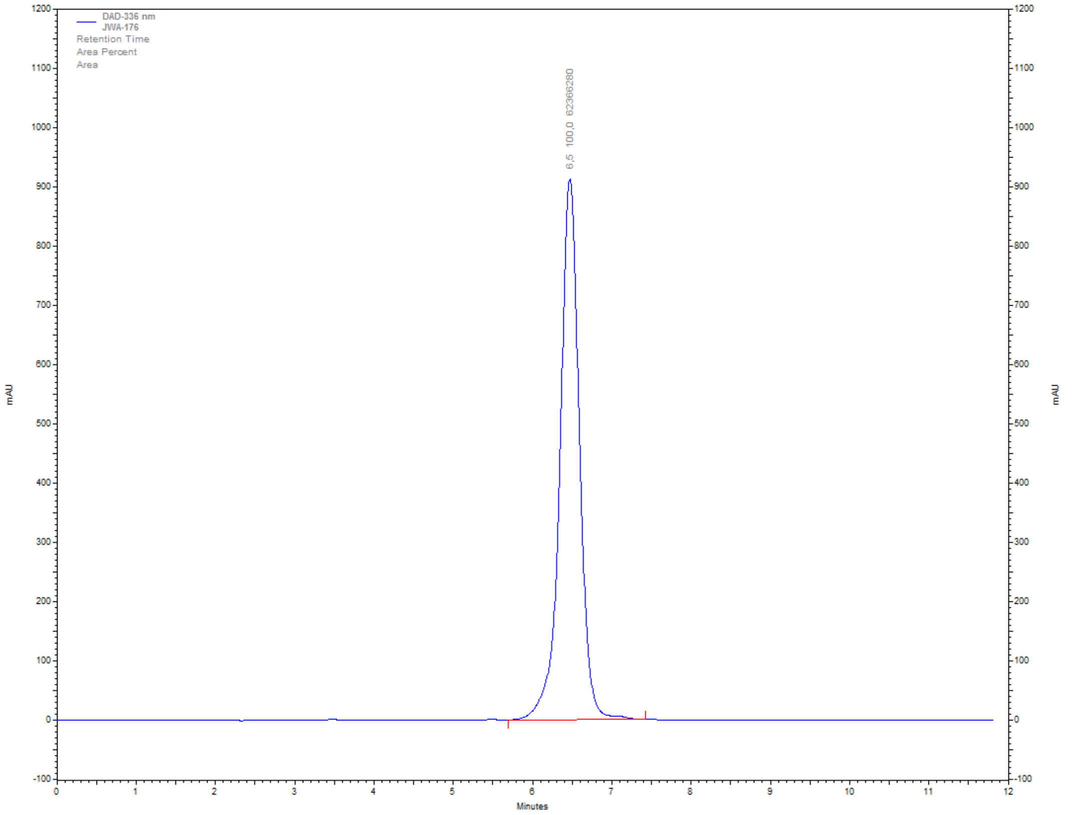

Figure S45. HPLC spectrum of **4**.

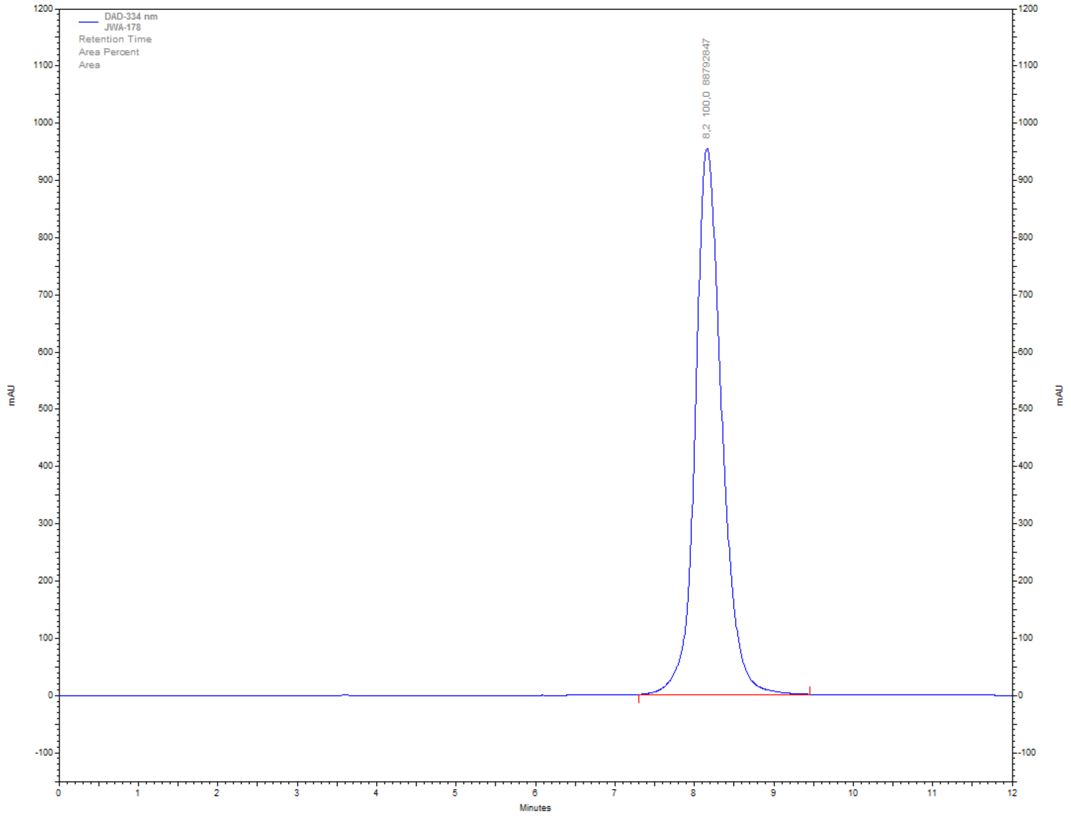

Figure S46. HPLC spectrum of **5**.

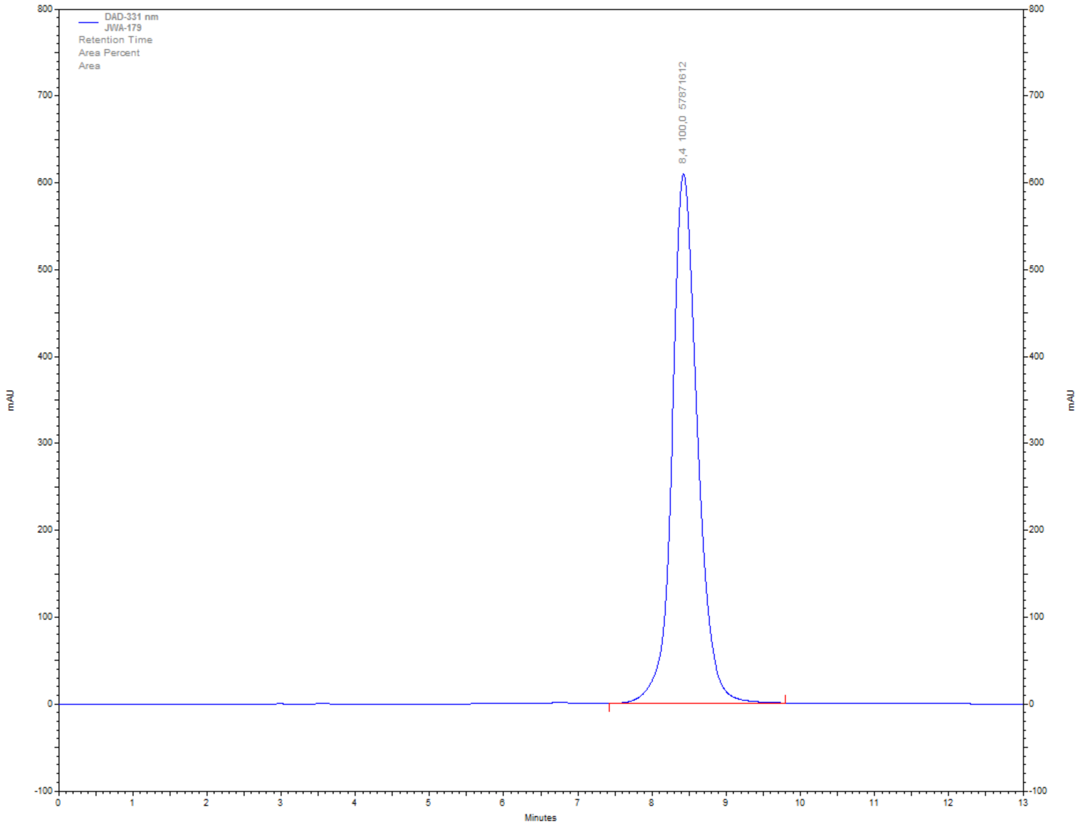

Figure S47. HPLC spectrum of **6**.

SI-10 Molecular geometries

In order to ensure that our results can be reproduced by other researchers, the present section lists selected molecular geometries of the truncated model of compound **1**. All geometries are given in terms of Cartesian coordinates in units of ångström (Å). The ground-state equilibrium geometries were optimized at the SOS-MP2/6-31G(d) level of theory, whereas the excited-state equilibrium geometries were optimized at the SOS-ADC(2)/6-31G(d) level.

S0-min-1

C	4.9158620	7.1111796	16.3031764
H	4.4326537	7.6404540	17.1238574
C	4.2171897	6.7153058	15.1477081
C	5.0155638	6.0635726	14.2063168
C	6.2797564	5.4887723	14.3472875
C	6.9586360	5.8963526	15.5104480
H	7.9597903	5.5428481	15.7551034
C	6.2735543	6.7286849	16.4287694
N	4.2900815	5.5101385	13.1850560
C	2.9443309	5.6820383	13.4810405
C	1.8731076	5.1420928	12.7691422
C	2.0347482	4.3220392	11.5225904
C	3.2375996	3.6107754	11.0536044
C	4.5818951	3.5328066	11.7172983
C	5.0227298	4.4467213	12.6742310
C	5.5209511	2.5399783	11.3707825
H	5.2431761	1.7904700	10.6351342
C	6.8031755	2.5126141	11.9465784
H	7.4926500	1.7264606	11.6427511
C	7.2155976	3.4480199	12.9073364
H	8.2116121	3.3881495	13.3445597
C	6.3084319	4.4398587	13.3015953
C	2.8388913	6.5029884	14.6480484
C	1.5524088	6.8156187	15.1054420
H	1.4025823	7.4376132	15.9871842
C	0.4529043	6.2883823	14.4116749
H	-0.5526109	6.5102686	14.7658866
C	0.5999729	5.4649430	13.2818529
H	-0.2806117	5.0733027	12.7806648
N	3.2013366	2.9245995	9.9131058
N	0.9126391	4.2698479	10.8061983
C	0.8860371	3.5527944	9.6462192
C	2.0435105	2.8784327	9.1896095
C	-0.3115416	3.4980963	8.8781415
H	-1.1841923	4.0402470	9.2370023
C	-0.3327564	2.7883501	7.6963355
H	-1.2272405	2.7636363	7.0771134

C	0.8339764	2.0925702	7.2482825
C	2.0067130	2.1288918	7.9836323
H	2.8951636	1.5868158	7.6671336
N	0.7765719	1.3787806	6.0249165
C	1.6437186	1.5431727	4.9358312
C	1.2459681	0.6637269	3.8992206
C	-0.1730810	0.4036385	5.6949878
C	2.7282022	2.4239835	4.7864585
H	3.0192979	3.1118417	5.5771440
C	3.4115760	2.3969426	3.5714116
H	4.2575157	3.0672111	3.4248750
C	3.0355271	1.5192829	2.5301366
C	1.9506322	0.6588224	2.6820289
H	1.6565834	-0.0105353	1.8739151
C	-0.7315917	-1.0617680	3.8259218
H	-0.5487842	-1.4288948	2.8163046
C	-1.7775100	-1.5772309	4.5884935
C	-2.0074486	-1.1160739	5.9038604
H	-2.8224442	-1.5455989	6.4849852
C	-1.2135048	-0.1241766	6.4785160
H	-1.3859381	0.2086167	7.4998963
H	-2.4187452	-2.3535698	4.1743971
H	3.5955637	1.5260875	1.5965030
C	0.0807970	-0.0562435	4.3797171
H	6.8060941	7.0376789	17.3275443

S0-min-2

C	4.8431373	6.9524001	16.4150859
H	4.3444100	7.3797164	17.2844600
C	4.1434441	6.5851199	15.2508273
C	4.9623085	6.0705360	14.2442905
C	6.2721168	5.5927569	14.3110180
C	6.9514070	5.9696127	15.4842525
H	7.9846676	5.6827774	15.6772028
C	6.2302819	6.6745129	16.4784933
N	4.2526510	5.5344639	13.2028506
C	2.9071091	5.5744912	13.5434427
C	1.8614884	5.0020139	12.8192448
C	2.0507561	4.2927202	11.5102719
C	3.2898065	3.7093938	10.9649177
C	4.6565051	3.6974726	11.5862016
C	5.0517242	4.5737979	12.5968877
C	5.6615200	2.8128332	11.1443466
H	5.4226369	2.0980433	10.3620298
C	6.9590140	2.8504392	11.6840323

H	7.6999456	2.1479893	11.3054663
C	7.3241049	3.7450499	12.7011297
H	8.3347197	3.7365313	13.1077920
C	6.3526498	4.6278357	13.1898980
C	2.7719168	6.2971504	14.7707310
C	1.4788453	6.4700008	15.2808217
H	1.3064550	7.0120784	16.2099223
C	0.4044746	5.9063840	14.5763386
H	-0.6044341	6.0193733	14.9701596
C	0.5825521	5.1822974	13.3845677
H	-0.2787468	4.7577420	12.8766475
N	3.2705775	3.0949226	9.7838677
N	0.9171226	4.2096143	10.8153461
C	0.9119985	3.5766470	9.6070823
C	2.0985162	3.0060954	9.0878118
C	-0.2987402	3.4862061	8.8634910
H	-1.2029499	3.9174488	9.2885370
C	-0.3052861	2.8393868	7.6459299
H	-1.2273068	2.7284378	7.0787221
C	0.8999673	2.2795095	7.1168427
C	2.0894150	2.3642157	7.8207063
H	3.0188687	1.9660055	7.4195507
N	0.8535615	1.6168874	5.8644831
C	1.2803388	0.3058565	5.6120627
C	1.0498007	0.0057918	4.2471089
C	0.3509169	2.1586260	4.6746605
C	1.8249183	-0.6391819	6.4978063
H	1.9823499	-0.4119856	7.5498039
C	2.1406614	-1.8944377	5.9794611
H	2.5650779	-2.6489060	6.6405618
C	1.9273637	-2.2080720	4.6185438
C	1.3745760	-1.2693228	3.7504086
H	1.2021521	-1.5167101	2.7032138
C	0.0236889	1.5006730	2.3481368
H	0.0931986	0.7638083	1.5483082
C	-0.4919181	2.7706641	2.0984809
C	-0.5680790	3.7343409	3.1287149
H	-0.9612175	4.7250774	2.9045001
C	-0.1497865	3.4483132	4.4277692
H	-0.1924727	4.2046951	5.2086494
H	-0.8278783	3.0318617	1.0963538
H	2.1891553	-3.1987639	4.2506381
C	0.4473200	1.1843473	3.6513292
H	6.7635660	6.9604758	17.3844209

S1-min-1

C	4.8546085	6.8747397	16.5377296
H	4.3964878	7.4533748	17.3386734
C	4.1395020	6.4424365	15.4034325
C	4.9383378	5.7324710	14.5176329
C	6.2393134	5.2471698	14.5699061
C	6.9284143	5.6983256	15.7105523
H	7.9626718	5.4284920	15.9197570
C	6.2160165	6.4928210	16.6484450
N	4.2357609	5.2297008	13.4626945
C	2.9222441	5.5937734	13.5753644
C	1.8994665	5.2185407	12.6951935
C	2.1016074	4.4127763	11.4713626
C	3.2857696	3.7128341	11.0156572
C	4.6431006	3.6932299	11.5860582
C	5.0353906	4.3760717	12.7466086
C	5.6847674	2.8871012	11.0400089
H	5.4734882	2.3100808	10.1464423
C	6.9651103	2.8616550	11.6095775
H	7.7209180	2.2309988	11.1430809
C	7.3147648	3.5794417	12.7753934
H	8.3240523	3.5200246	13.1802101
C	6.3358585	4.3857183	13.3592440
C	2.7925115	6.3836713	14.7739050
C	1.5215656	6.8701207	15.0709159
H	1.3356899	7.4764210	15.9564722
C	0.4595301	6.5357091	14.1985147
H	-0.5365356	6.9134545	14.4243587
C	0.6269878	5.7545326	13.0503806
H	-0.2225154	5.5245016	12.4171487
N	3.2546933	2.9752249	9.8385587
N	0.9101242	4.3169015	10.7847469
C	0.8896095	3.5845643	9.6430116
C	2.0861059	2.9141195	9.1577561
C	-0.3184292	3.4723766	8.9090107
H	-1.1950997	3.9990956	9.2828929
C	-0.3687885	2.7301944	7.7320649
H	-1.2795932	2.6902728	7.1380915
C	0.8023722	2.0745039	7.2613585
C	2.0139232	2.1650056	7.9639065
H	2.8977173	1.6246953	7.6312020
N	0.7415608	1.3473325	6.0527070
C	1.6445148	1.4512540	4.9819155
C	1.2342389	0.5722129	3.9500410
C	-0.2396094	0.4032689	5.7135080
C	2.7552892	2.2991397	4.8341522
H	3.0495711	2.9956814	5.6149939

C	3.4624292	2.2238092	3.6342592
H	4.3308442	2.8654173	3.4910575
C	3.0780399	1.3392044	2.6028507
C	1.9600743	0.5194505	2.7480855
H	1.6554898	-0.1507076	1.9445826
C	-0.8040391	-1.0772497	3.8575794
H	-0.6100046	-1.4690839	2.8594331
C	-1.8829529	-1.5456075	4.6059723
C	-2.1218478	-1.0592127	5.9099497
H	-2.9584623	-1.4567726	6.4829771
C	-1.3057404	-0.0863205	6.4871050
H	-1.4804190	0.2611649	7.5023728
H	-2.5411394	-2.3066204	4.1898853
H	3.6561574	1.3087704	1.6807057
C	0.0282348	-0.0913718	4.4138643
H	6.7595237	6.8315255	17.5296429

S1-min-2

C	4.8342937	6.8238479	16.5670409
H	4.3694663	7.3727698	17.3848440
C	4.1157025	6.3823845	15.4382758
C	4.9249218	5.7162578	14.5287894
C	6.2462057	5.2869243	14.5452619
C	6.9377503	5.7460292	15.6811684
H	7.9867306	5.5179498	15.8652224
C	6.2108908	6.4923825	16.6474574
N	4.2264612	5.2151625	13.4705901
C	2.9045754	5.5390959	13.6030646
C	1.8858535	5.1631262	12.7190633
C	2.0996622	4.3910989	11.4746359
C	3.3010339	3.7403515	10.9895813
C	4.6666854	3.7617561	11.5363638
C	5.0492062	4.4203276	12.7147678
C	5.7334173	3.0204879	10.9474679
H	5.5329314	2.4660123	10.0373200
C	7.0230618	3.0324034	11.4963269
H	7.7976796	2.4523310	10.9961869
C	7.3613110	3.7241381	12.6813262
H	8.3791675	3.6960931	13.0675951
C	6.3584048	4.4674673	13.3068230
C	2.7643105	6.2981873	14.8206849
C	1.4831490	6.7404807	15.1402912
H	1.2884744	7.3205692	16.0413806
C	0.4233499	6.4011699	14.2666777
H	-0.5806006	6.7457270	14.5099213

C	0.6023611	5.6548897	13.0975297
H	-0.2453929	5.4206330	12.4634873
N	3.2730668	3.0133636	9.8046173
N	0.9062980	4.2787432	10.7970656
C	0.8925733	3.5676678	9.6414181
C	2.0941689	2.9125957	9.1483513
C	-0.3150421	3.4554960	8.9072645
H	-1.2050839	3.9370584	9.3094279
C	-0.3600416	2.7302990	7.7197149
H	-1.2979802	2.5958682	7.1849136
C	0.8189196	2.0989940	7.2345764
C	2.0293714	2.1877640	7.9384983
H	2.9420620	1.7454531	7.5448346
N	0.7522703	1.3510030	6.0389522
C	1.2546981	0.0532669	5.8483353
C	0.9839589	-0.3517500	4.5184848
C	0.1680307	1.7797293	4.8370856
C	1.8740141	-0.8058874	6.7719051
H	2.0516743	-0.5051324	7.8011822
C	2.2423043	-2.0729576	6.3198564
H	2.7285586	-2.7602024	7.0109319
C	1.9976362	-2.4851232	4.9915850
C	1.3596035	-1.6358350	4.0891319
H	1.1568072	-1.9614905	3.0691664
C	-0.2040567	0.9472911	2.5733301
H	-0.1202793	0.1624434	1.8220129
C	-0.7975368	2.1690494	2.2596000
C	-0.8834008	3.1977099	3.2231520
H	-1.3316247	4.1513398	2.9473934
C	-0.3990317	3.0258311	4.5198587
H	-0.4436060	3.8338568	5.2458304
H	-1.1855938	2.3413216	1.2569963
H	2.3011783	-3.4819793	4.6759263
C	0.2845933	0.7468484	3.8753332
H	6.7569806	6.8374462	17.5245821

T1-min-1

C	5.0395368	7.2524913	16.1393995
H	4.6057394	7.9383694	16.8662180
C	4.3192642	6.7703440	15.0279197
C	5.0749705	5.9211651	14.2221676
C	6.2968841	5.2833065	14.4283384
C	6.9888561	5.7762465	15.5484852
H	7.9633350	5.3945539	15.8509857
C	6.3504598	6.7597316	16.3488925

N	4.3286960	5.3309176	13.2460291
C	3.0187083	5.7186178	13.3992206
C	1.9311211	5.2310945	12.6253504
C	2.0342549	4.2030487	11.6412862
C	3.2292124	3.4879869	11.1183187
C	4.5506947	3.3370019	11.8098125
C	5.0168576	4.2328082	12.7681218
C	5.4556388	2.3024279	11.4859606
H	5.1612389	1.5656004	10.7438476
C	6.7119026	2.2139384	12.1065333
H	7.3714448	1.3929990	11.8295762
C	7.1475274	3.1387323	13.0738634
H	8.1271346	3.0291212	13.5375704
C	6.2873107	4.1809261	13.4314064
C	2.9599279	6.6873310	14.4447230
C	1.7032330	7.2345293	14.7521383
H	1.5810494	7.9750201	15.5410259
C	0.5873042	6.7929858	13.9992251
H	-0.3928621	7.2060867	14.2345294
C	0.6693109	5.8232689	13.0036649
H	-0.2242704	5.5029334	12.4794232
N	3.1870778	2.8249836	9.9796178
N	0.7655677	3.9419310	11.0184798
C	0.7700418	3.2963471	9.8645402
C	1.9865876	2.7861218	9.2657158
C	-0.4740212	3.0728228	9.1676358
H	-1.3840567	3.4574763	9.6242242
C	-0.4902485	2.4561525	7.9309250
H	-1.4223773	2.3490052	7.3800956
C	0.7182131	2.0017914	7.3429809
C	1.9558487	2.1606595	8.0327092
H	2.8721435	1.7381616	7.6257931
N	0.7034179	1.3770176	6.0834244
C	1.5641469	1.6734077	5.0113405
C	1.2376043	0.8368789	3.9176992
C	-0.1736161	0.3559605	5.6765083
C	2.5627541	2.6570717	4.9201401
H	2.7858441	3.3266112	5.7472024
C	3.2517918	2.7586789	3.7113306
H	4.0351658	3.5084543	3.6105283
C	2.9534584	1.9177089	2.6172778
C	1.9409834	0.9641819	2.7077579
H	1.7001563	0.3284442	1.8562342
C	-0.5968596	-1.0264544	3.7109080
H	-0.3770274	-1.3103755	2.6821632
C	-1.5991759	-1.6775644	4.4276322
C	-1.8681867	-1.3291505	5.7690921

H	-2.6423418	-1.8662617	6.3152515
C	-1.1609595	-0.3157558	6.4163781
H	-1.3590502	-0.0759235	7.4580734
H	-2.1722949	-2.4742398	3.9564521
H	3.5128332	2.0276901	1.6898268
C	0.1251380	0.0028272	4.3387777
H	6.8962355	7.1341847	17.2142136

T1-min-2

C	5.0246476	7.1474572	16.2507341
H	4.5873174	7.8177859	16.9898132
C	4.3159057	6.7032698	15.1160695
C	5.0754876	5.8686311	14.2992051
C	6.2893806	5.2155032	14.5023318
C	6.9696971	5.6705002	15.6453465
H	7.9373198	5.2732169	15.9497047
C	6.3275938	6.6359606	16.4649018
N	4.3394529	5.3155753	13.2943466
C	3.0330021	5.7172420	13.4331985
C	1.9572873	5.2769090	12.6148145
C	2.0637245	4.2784375	11.6011478
C	3.2528350	3.5409241	11.0938851
C	4.5666745	3.3676859	11.7965704
C	5.0260861	4.2293091	12.7891383
C	5.4711028	2.3377689	11.4538418
H	5.1828964	1.6265709	10.6851193
C	6.7180622	2.2217635	12.0875277
H	7.3766500	1.4063536	11.7925504
C	7.1456952	3.1115149	13.0909016
H	8.1177946	2.9806585	13.5647471
C	6.2866647	4.1466176	13.4693457
C	2.9672056	6.6563089	14.5051057
C	1.7133105	7.2161170	14.8008585
H	1.5852735	7.9347349	15.6087761
C	0.6084787	6.8206816	14.0056786
H	-0.3693054	7.2454652	14.2298353
C	0.6971967	5.8838165	12.9805388
H	-0.1877980	5.6019458	12.4212418
N	3.2110051	2.8714625	9.9584732
N	0.8093099	4.0871342	10.9279043
C	0.8163932	3.4267307	9.7820095
C	2.0199131	2.8398476	9.2320215
C	-0.4142760	3.2678364	9.0449014
H	-1.3233548	3.6722063	9.4860882
C	-0.4251691	2.6302033	7.8192796

H	-1.3582691	2.4913010	7.2770172
C	0.7741161	2.0974148	7.2787860
C	1.9952873	2.2000957	8.0046337
H	2.9206733	1.8049146	7.5913436
N	0.7593460	1.4525706	6.0281961
C	1.3372757	0.2042101	5.7378873
C	1.1107134	-0.0987175	4.3740064
C	0.1593387	1.9449089	4.8561338
C	1.9892633	-0.6950730	6.5979777
H	2.1251927	-0.4812064	7.6552869
C	2.4410455	-1.8943768	6.0468886
H	2.9540786	-2.6106476	6.6871016
C	2.2437293	-2.2022612	4.6832239
C	1.5691543	-1.3170801	3.8443787
H	1.4012813	-1.5647300	2.7966289
C	-0.1311241	1.2868584	2.5246475
H	0.0116844	0.5753064	1.7118940
C	-0.7974176	2.4908409	2.3042635
C	-0.9618537	3.4255110	3.3497218
H	-1.4688749	4.3680991	3.1482024
C	-0.4847839	3.1737859	4.6361564
H	-0.5943532	3.9126366	5.4261698
H	-1.1833839	2.7232916	1.3132100
H	2.6108284	-3.1486241	4.2897497
C	0.3525305	1.0066624	3.8141239
H	6.8643156	6.9810376	17.3479078

SI-11 References

1. Patel, D. G. “Dan”; Ohnishi, Y.; Yang, Y.; Eom, S.-H.; Farley, R. T.; Graham, R. T.; Xue, J.; Hirata, S.; Schanze, K. S.; Reynolds, J. R.; *JOURNAL OF POLYMER SCIENCE PART B: POLYMER PHYSICS* **2011**, *49*, 557–565.
2. Bender, A. M.; Griggs, N. W.; Gao, C.; Trask, T. J.; Traynor, J. R.; Mosberg, H. I.; *ACS Med. Chem. Lett.* **2015**, *6*, 1199–1203.
3. APEX3 V2019, Bruker Nano, Inc., **2019**.
4. SAINT V8.40A, Bruker Nano, Inc., **2019**.
5. SADABS V2016/2, Bruker Nano, Inc., **2019**.
6. *G. M. Sheldrick*, *Acta Cryst.*, **2015**, *A71*, 3-8.
7. G. M. Sheldrick, *Acta Cryst*., **2015**, *C71*, 3–8.
8. *International Tables for Crystallography*, Ed. A. J. C. Wilson, Kluwer: Dordrecht, **1992**, Vol.C.
9. C. F. Macrae, I. J. Bruno, J. A. Chisholm, P. R. Edgington, P. McCabe, E. Pidcock, L. Rodriguez-Monge, R. Taylor, J. van de Streek and P. A. Wood, “Mercury CSD 2.0 - New Features for the Visualization and Investigation of Crystal Structures”, *J. Appl. Cryst.*, **2008**, 41, 466-470.
10. Wagner, J.; Zimmermann Crocomo, P.; Kochman, M. A.; Kubas, A.; Data, P.; Lindner, M. Modular Nitrogen-Doped Concave Polycyclic Aromatic Hydrocarbons for High-Performance Organic Light-Emitting Diodes with Tunable Emission Mechanisms**. *Angew. Chem. Int. Ed.* **2022**. https://doi.org/10.1002/anie.202202232.
